# Involucratusins A–H: Unusual Cadinane Dimers from *Stahlianthus involucratus* with Multidrug Resistance Reversal Activity

**DOI:** 10.1038/srep29744

**Published:** 2016-07-13

**Authors:** Qiang-Ming Li, Jian-Guang Luo, Rui-Zhi Wang, Xiao-Bing Wang, Ming-Hua Yang, Jun Luo, Ling-Yi Kong

**Affiliations:** 1 State Key Laboratory of Natural Medicines, Department of Natural Medicinal Chemistry, China Pharmaceutical University, 24 Tong Jia Xiang, Nanjing 210009, People’s Republic of China

## Abstract

Three novel cadinane dimers, involucratusins A–C (**1**–**3**), five unique nor-cadinane-dimers, involucratusins D–H (**4**–**8**), together with a known compound (**9**) were isolated from the rhizomes of *Stahlianthus involucratus*. Their challenging structures and absolute configurations were determined by spectroscopic data, CD experimentation, chemical conversions and single-crystal X-ray diffraction. Compounds **1**–**3** are unusual cadinane dimers with new connection and novel cores. Compound **4** is a unique nor-cadinane-dimer, and **5** and **6** are two pairs of hemiketal racemates with novel dinor-cadinane-dimer backbone. Compounds **7** and **8** represent unusual dodecanor-cadinane-dimer and tetradecanor-cadinane-dimer carbon skeletons, respectively. The possible biogenetic pathways of **1**–**8** were proposed, involving nucleophilic addition, S_N_2 nucleophilic displacement, [3 + 3] benzannulation, oxidative cleavage, decarboxylation, and oxidative phenol coupling reactions. Multidrug resistance (MDR) reversal activity assay of the isolates were evaluated in doxorubicin-resistant human breast cancer cells (MCF-7/DOX). The combined use of these novel cadinane dimers at a concentration of 10 *μ*M increased the cytotoxicity of doxorubicin by 2.2–5.8-fold. It is the first report about the MDR reversal activity of cadinane dimers.

Sesquiterpenoid dimers are some naturally occurring metabolites that have C_30_ cores, and originate biosynthetically from two sesquiterpenoid molecules^1^. Cadinane dimers, as one class of sesquiterpenoid dimers, are of considerable interest due to their ability to induce diverse biological activity (e.g. male antifertility, anticancer, antiviral, and anti-inflammatory)^1^. Of these naturally occurring cadinane dimers, most are derived from two units of the corresponding monomers by the free-radical coupling reaction^1^, however, rare example that formed by nucleophilic addition reaction have been reported. The occurrence of nor-cadinane-dimer is also surprisingly low, with the only two, aquatidial^2^ and parviflorene I^3^. Recently, we have reported three unprecedented cadinane dimers, involucratustones A–C^4^, from *Stahlianthus involucratus*, a folklore medicine to treat inflammation, pain, and fever^5^, as part of a continued program searching for novel terpenoids from Zingiberaceae plants^6^,^7^,^8^,^9^. These prompt us to perform a further phytochemical investigation on *S. involucratus*. As a result, three unusual cadinane dimers, involucratusins A–C (**1**–**3**) (Fig. 1), five novel nor-cadinane-dimers, involucratusins D–H (**4**–**8**), and a biosynthetically related known compound parviflorene D (**9**)^3^,^10^ were isolated and elucidated. Structurally, the architectures of **1**–**8** are different from the previously isolated cadinane dimers and nor-cadinane-dimers and represent five new carbon skeletons. Moreover, from the viewpoint of biosynthesis, the key biosynthesis reactions of cadinane dimers **1**–**3**, nucleophilic addition, and nor-cadinane-dimers **5**–**8**, oxidative cleavage, are extremely rare in the biosynthesis of cadinane dimers and norterpenoids, respectively. Herein are described the isolation, structure elucidation and possible biosynthesis of new metabolites **1**–**8**, as well as MDR reversal activities of the compounds obtained.

## Results and Discussion

### Structure elucidation

Involucratusin A (**1**) was isolated as white columnar crystals (MeOH:H_2_O = 10:1). Its molecular formula C_30_H_38_O_5_, corresponding to 12 degrees of unsaturation, was established by HRESIMS data (*m/z* 501.2613 [M+Na]^+^, calcd for C_30_H_38_O_5_Na, 501.2611). The ^1^H NMR spectrum of **1** showed the presence of two 1,2,3,5-tetrasubstituted benzene rings [*δ*_H_ 6.40 (1H, br s), 6.55 (1H, br s), 6.66 (1H, br s), 6.69 (1H, br s)], two isopropyl groups [*δ*_H_ 1.95 (1H, m), 1.22 (1H, d, 6.5 Hz), 0.79 (1H, d, 6.5 Hz), 1.84 (1H, m), 0.87 (1H, d, 6.5 Hz), 0.77 (1H, d, 6.5 Hz)] and three tertiary methyls [*δ*_H_ 2.29 (3H, s), 2.27 (3H, s), 1.92 (3H, s)]. Its ^13^C NMR spectrum displayed a total of thirty carbons that, with the aid of HSQC spectrum, were deduced to contain a ketonic (*δ*_C_ 207.3), twelve aromatic (*δ*_C_ 117.1, 117.2, 119.9, 121.6, 123.2, 123.4, 136.4, 137.7, 139.8, 140.4, 155.3, 155.8), and seven methylic carbons (Supplementary Table S1). According to the data above, compound **1** should contain two aromatic cadinane sesquiterpenoid moieties, which was further confirmed by 2D NMR spectra analysis (^1^H-^1^H COSY correlations: H-2/H-4/H_3_-15, H-8/H_2_-7/H-6/H-11/H_3_-12/H_3_-13, H-2′/H-4′/H_3_-15′, H_2_-7′/H-6′/H-11′/H_3_-12′/H_3_-13′; and HMBC correlations: H-2 with C-4/C-10, H-4 with C-6/C-10, H_3_-14 with C-8/C-9/C-10, H_3_-15 with C-2/C-3/C-4, H-2′ with C-4′/C-10′, H-4′ with C-6′/C-10′, H_2_-14′ with C-8′/C-9′/C-10′, H_3_-15′ with C-2′/C-3′/C-4′) (Fig. 2A).

**Figure 1:**
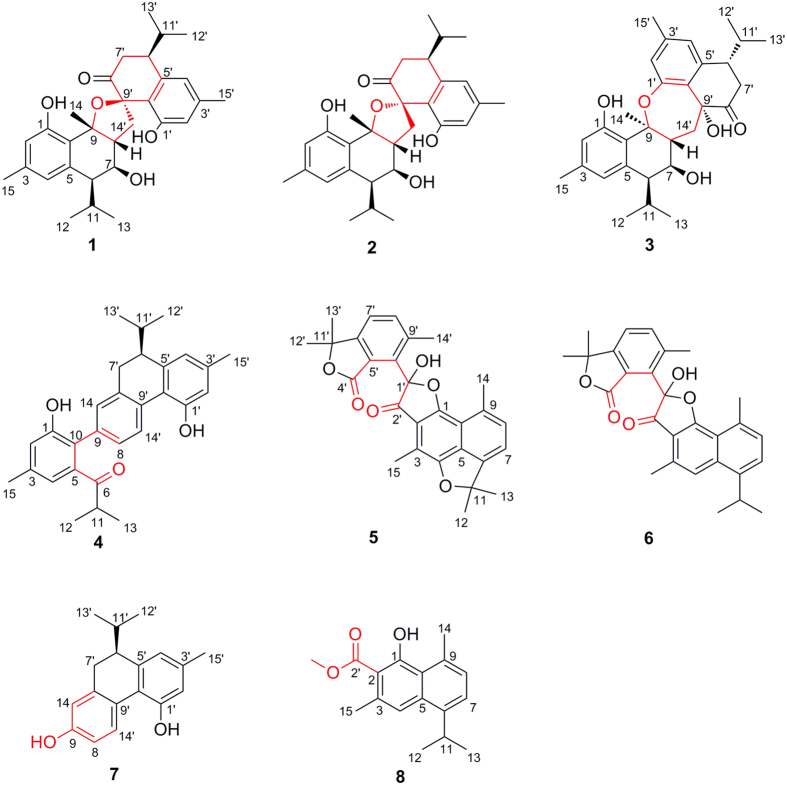
Structures of compounds 1–8.

**Figure 2:**
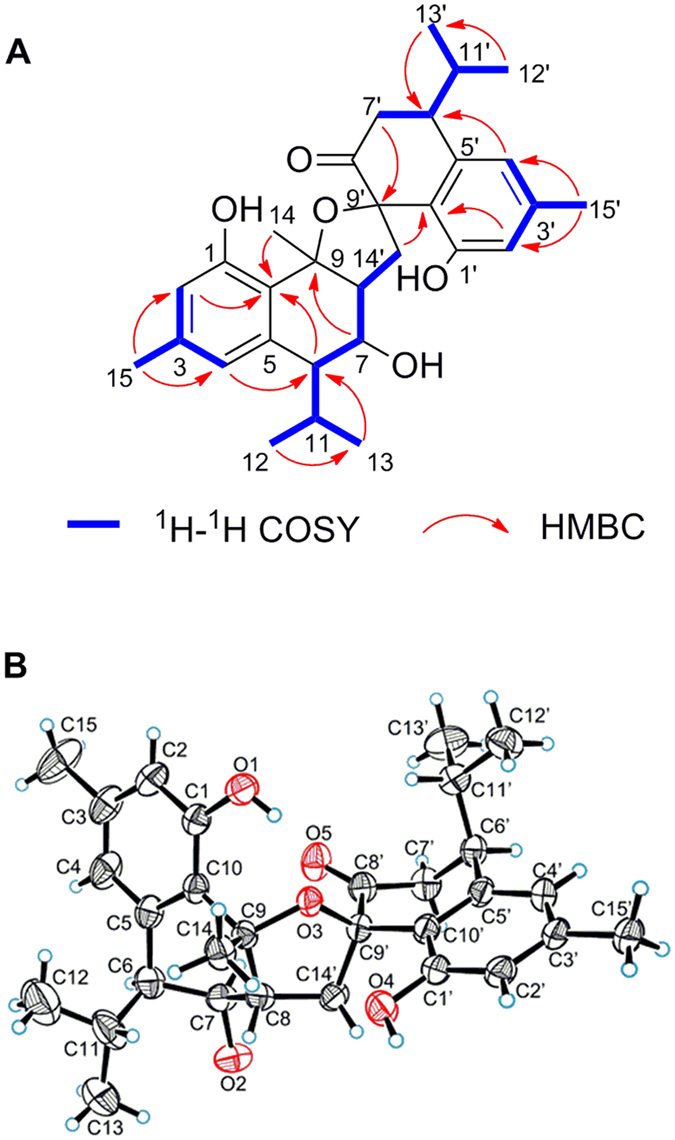
Key HMBC, ^1^H-^1^H COSY correlations (**A**) and X-ray structure (**B**) of compound **1**.

Further analysis, the carbon-carbon connectivity of two determined cadinane sesquiterpenoid moieties was established by the HMBC correlations from H-8 to C-9′ and C-14′; and from H_2_-14′ to C-7, C-8 and C-9, which was further confirmed by the ^1^H-^1^H COSY correlations of H-8 with H_2_-14′. The remaining four oxygen and three hydrogen atoms in molecular formula were incorporated with the five oxygenated carbons C-1 (*δ*_C_ 155.3), C-7 (*δ*_C_ 73.4), C-9 (*δ*_C_ 90.4), C-1′ (*δ*_C_ 155.8) and C-9′ (*δ*_C_ 91.4) to deduce the presence of three hydroxyl groups and one ether bond. However, due to the absence of the key HMBC correlations, the locations of the hydroxyl groups and ether bond could not be determined to completely establish the planar structure of **1**. After numerous attempts, a single crystal was obtained from MeOH/H_2_O (10:1) and subjected to an X-ray diffraction experiment using a mirror Cu K*α* radiation. Thus, as shown in Fig. 2B, compound **1** was established as a novel cadinane dimer with an unusual 1-oxaspiro[4.5]decane ring and a new manner of bond connection (C-8 and C-14′, C-9 and oxygen on C-9′) (Fig. 1).

In the ROESY spectrum of **1** (Supplementary Fig. S2), the ROE correlations of H-8 (*δ*_H_ 2.81) with H-11/H_3_-13/H_3_-14; H_3_-14 (*δ*_H_ 1.92) with H-11/H_3_-13; and H-7 with H-6/H-14′b indicated the relative configurations of C-6, C-7, C-8 and C-9 were R^*^, R^*^, R^*^ and R^*^, respectively. However, the relative configurations of C-6′ and spiro C-9′ were still unable to be established by ROESY spectrum due that both C-8′ and C-10′ are quarternary carbons and the absence of key correlation of H_2_-14′ with H-6′ or H-11′. The completely relative configuration of **1** was finally established by the successful X-ray diffraction experiment (Fig. 2B). Moreover, the absolute configuration of **1** was also assigned as 6*R*, 7*R*, 8*R*, 9*R*, 6′*S*, 9′*R* by Flack absolute structure parameter −0.03(9).

On the basis of analysis of the MS, 1D, and 2D NMR data (Supplementary Table S1, Fig. S1), involucratusin B (**2**) was shown to possess the same planar structure as **1**. The slight differences in the ^13^C NMR chemical shifts of C-8′, C-9′ and C-10′ suggested that **2** might be a stereoisomer of **1** at C-9′^11^,^12^,^13^. In the ROESY spectrum of **2** (Supplementary Fig. S2), the cross-peaks of H-11 with H-8/H_3_-14; H-7 with H-6/H-14′b suggested the relative configurations of C-6, C-7, C-8 and C-9 were R^*^, R^*^, R^*^ and R^*^, respectively. Although the ROE correlations of H-14′a with H-11′/H_3_-12′/H_3_-13′ deduced the isopropyl at C-6′ and CH_2_-14′ hold same orientation, the relative configurations of the C-6′ and spiro C-9′ could not be established due to the C-8′ and C-10′ were quaternary carbons.

**Figure 3:**
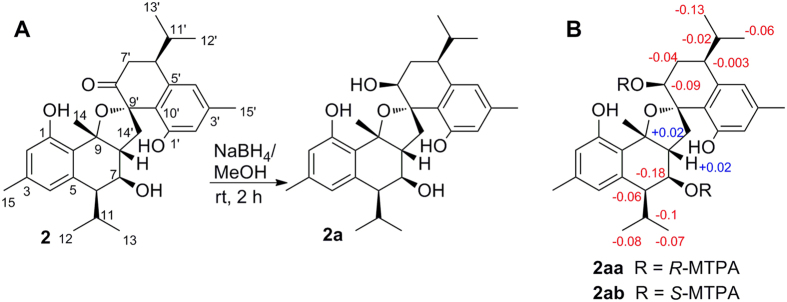
(**A**) Preparation of compound **2a** from compound **2;** (**B**) ^1^H NMR chemical shift differences of the MTPA ester derivatives of compound **2a**.

To completely determine the relative and absolute configurations of **2**, the ketone at C-8′ was reduced to the secondary alcohol to afford the major products **2a** (Fig. 3A and Supplementary Fig. S1, NMR data in Supplementary Table S1). Firstly, the relative configuration of **2a** was established as 6R^*^, 7R^*^, 8R^*^, 9R^*^, 6′S^*^, 8′S^*^, 9′R^*^ by the ROE correlations of H-11 with H-8/H_3_-14; H-7 with H-6/H-14′b; H-14′a with H-11′/H_3_-13′; and H-8′ with H-8/H_3_-14/H-6′ (Supplementary Fig. S2). Subsequently, using the modified Mosher’s method^14^,^15^, the absolute configurations of C-7 and C-8′ of **2a** were determined as *R* and *S*, respectively, on the basis of the analysis of the diagnostic proton chemical shift values (positive *Δδ*_*S-R*_ values for H-8 and H-14′a, and negative *Δδ*_*S-R*_ values for H-6, H-7, H-11, H_3_-12, H_3_-13, H-6′, H-7′b, H-8′, H-11′, H_3_-12′ and H_3_-13′) between protons of the C-7 and C-8′ bis-(*S*)- and bis-(*R*)- MTPA esters of **2a** (**2ab** and **2aa**) (Fig. 3B). Thus, the absolute configurations of the chiral centers in **2a** were assigned as 6*R*, 7*R*, 8*R*, 9*R*, 6′*S*, 8′*S*, 9′*R*, respectively. Finally, according to the absolute configuration of **2a** determined above, the absolute configuration of **2** was undoubtedly established as 6*R*, 7*R*, 8*R*, 9*R*, 6′*S*, 9′*S*, which unambiguously confirmed **2** was a stereoisomer of **1** at C-9′.

Involucratusin C (**3**) was assigned to share the same molecular formula C_30_H_38_O_5_ as **1** and **2** by its HRESIMS data (*m/z* 501.2610 [M+Na]^+^, calcd for C_30_H_38_O_5_Na, 501.2611), which deduced **3** might also be a cadinane dimer. The 1D NMR (Supplementary Table S1) of **3** was incorporated with the HMBC spectrum (Supplementary Fig. S1) to show the presence of two cadinane sesquiterpenoid units, which further confirmed that **3** was a cadinane dimer. Further analysis, the HMBC correlations of H_2_-14′ with C-7/C-8/C-9, and H-8 with C-9′/C-14′ established the carbon-carbon connectivity of two cadinane sesquiterpenoid moieties of **3**. According to the molecular formula C_30_H_38_O_5_ and the five oxygenated carbons (C-1: *δ*_C_ 155.8; C-7: *δ*_C_ 70.7; C-9: *δ*_C_ 87.4; C-1′: *δ*_C_ 152.2; C-9′: *δ*_C_ 76.1), the remaining four oxygen atoms and three hydrogen atoms indicated the presence of three hydroxyl groups and an ether bond. To determine the position of the hydroxyl groups and ether bond, 1D and 2D NMR spectra of **2** were recorded in DMSO-*d*_6_. The HMBC correlations of the hydroxyl proton at *δ*_H_ 8.90 with C-1/C-2/C-10; the hydroxyl proton at *δ*_H_ 4.95 with C-6/C-7/C-8; and the hydroxyl proton at *δ*_H_ 5.26 with C-8′/C-9′/C-10′/C-14′ suggested the hydroxyl groups located at C-1, C-7 and C-9′, respectively, and the ether bond linked with C-9 and C-1′ to form a novel seven-membered cyclic ether. Thus, the planar structure of compound **3** was deduced as a novel seven-membered cyclic ether cadinane dimer with new manner of bond connection (C-8 and C-14′, C-9 and oxygen on C-1′) (Fig. 1).

**Figure 4:**
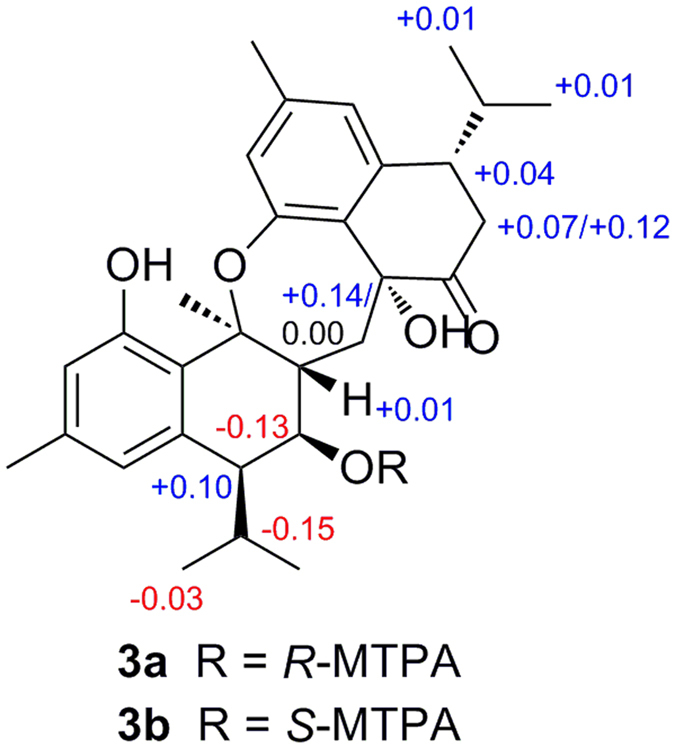
^1^H NMR chemical shift differences of the MTPA ester derivatives of compound 3.

In the ROESY spectrum (Supplementary Fig. S2), the cross-peaks of H-8 with OH-7/H-11/H_3_-12/H_3_-13; OH-7 with H-11; and H-7 with H-6/H_3_-14/H-14′b indicated the relative configurations of the chiral centers C-6, C-7, C-8 and C-9 were R^*^, R^*^, R^*^ and S^*^, respectively. The relative configurations of C-6′ and C-9′ were deduced as S^*^ and R^*^ by the ROE correlations of OH-9′ with H_3_-14/H-11′/H_3_-13′/H-14′b; H-7′b with H-11′/H_3_-13′, and H-7′a with H-14′a. Having assigned the relative configuration of **3**, we next attempted to determine the absolute configuration via a modified Mosher’s method^14^,^15^. The absolute configuration of C-7 was established to be *R* by the analysis of the diagnostic proton chemical shift values (positive *Δδ*_*S-R*_ values for H-8, H-6′, H_2_-7′, H_3_-12′, H_3_-13′ and H-14′a, and negative *Δδ*_*S-R*_ values for H-7, H-11 and H_3_-12) between protons of the C-7 (*S*)- and (*R*)- MTPA esters of **3** (**3b** and **3a**) (Fig. 4), although an anomalous positive *Δδ*_*S-R*_ value for H-6 appeared. The reason of the irregularity of H-6 would be the conformation of the MTPA moieties slightly different from the ideal one, which might be owing to the compression of other groups in **3a** and **3b**^16^,^17^,^18^. Combining with the relative configuration established above, the absolute stereochemistry of **3** was determined as 6*R*, 7*R*, 8*R*, 9*S*, 6′*S*, 9′*R*.

Involucratusin D (**4**) was obtained as a yellowish oil, and was deduced to hold the molecular formula C_29_H_32_O_3_ by its HRESIMS data (*m/z* 451.2245 [M+Na]^+^, calcd for C_29_H_32_O_3_Na, 451.2244), revealing **4** might be a nor-cadinane-dimer. From the 1D NMR data (Supplementary Table S2), the presence of two 1,2,3,5-tetrasubstituted and one 1,2,4-trisubstituted benzene rings were deduced by three groups of signals at *δ*_H_ 6.91 (br s) and 6.81 (br s), and at 6.59 (br s) and 6.62 (br s), and at 7.20 (br d, 8.0), 8.28 (br d, 8.0) and 7.15 (br s) in the ^1^H NMR incorporating with the eighteen aromatic carbons in the ^13^C NMR. Interpretation of the ^1^H-^1^H COSY and HSQC spectra led to the assignment of an isopropyl group (H-11/H_3_-12/H_3_-13) and an isopentane group (H_2_-7′/H-6′/H-11′/H_3_-12′/H_3_-13′) (Supplementary Fig. S1). These five substructures combining with the remaining two methyl groups [*δ*_H_ 2.31 (s), *δ*_C_ 21.3; *δ*_H_ 2.37 (s), *δ*_C_ 21.4] and one carbonyl group (*δ*_C_ 211.7) were connected by the key HMBC correlations of H-4 with C-6; H-11 with C-5/C-6; H_3_-15 with C-2/C-3/C-4; H-8 with C-10; H-14 with C-10/C-7′; H-6′ with C-4′/C-5′/C-8′/C-10′; H_2_-7′ with C-14/C-5′/C-8′/C-9′; H-14′ with C-10′; H_3_-15′ with C-2′/C-3′/C-4′ to establish the novel aromatic 6,7-seco-7-nor-cadinane-dimer carbon skeleton. The oxygenated aromatic carbons C-1 (*δ*_C_ 152.8) and C-1′ (*δ*_C_ 153.2) were incorporated with the remaining two oxygen and two hydrogen atoms in molecular formula to indicate the presence of OH-1 and OH-1′. Thus, the unusual planar structure of **4** was established as shown in Fig. 1.

**Figure 5:**
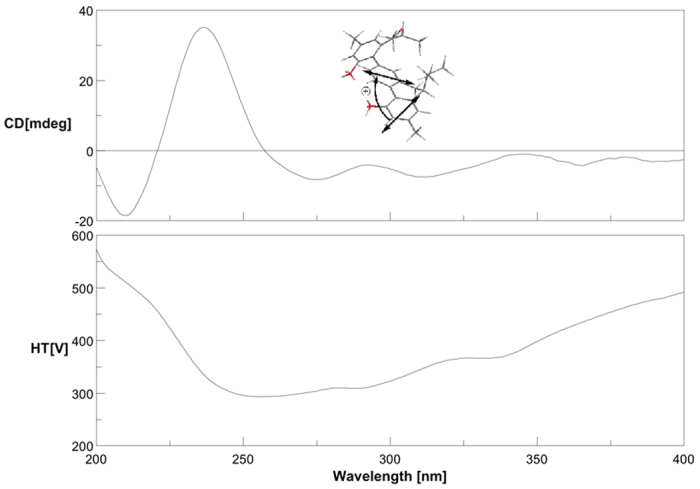
CD and exciton chirality sign of compound 4.

The absolute configuration at C-6′ of **4** was determined by application of the exciton chirality method^19^,^20^,^21^. The positive split [Δ*ε* +35.1 (237 nm), −18.5 (210 nm)] in CD spectrum of **4** (Fig. 5) indicated the clockwise screw sense between the two long axes of the phenyl chromophores, leading to the establishment of the 6′*S* configuration in **4**.

**Figure 6:**
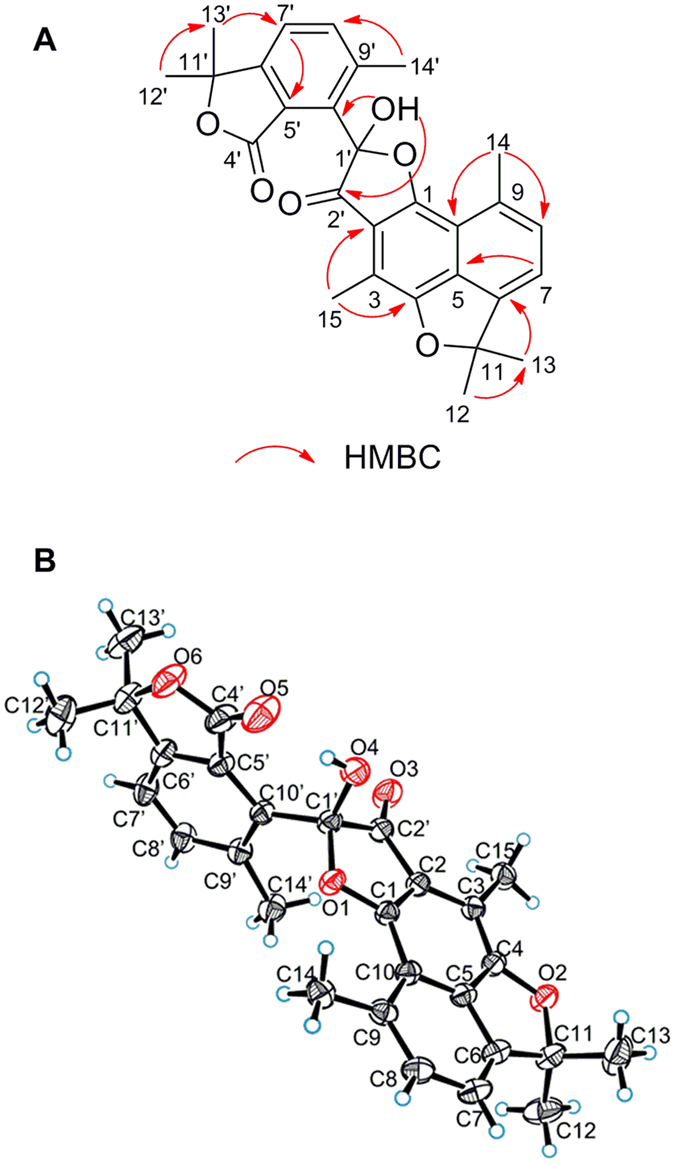
Key HMBC correlations (**A**) and X-ray structure (**B**) of compound **5**.

Involucratusin E (**5**) was obtained as lamellar crystals (MeOH:H_2_O = 10:1). The pseudomolecular ion at *m/z* 459.1804 [M+H]^+^ (calcd for C_28_H_27_O_6_, 459.1802) in the HRESIMS of compound **5** determined that it possessed a molecular formula of C_28_H_26_O_6_, which indicated **5** might be a dinor-cadinane-dimer. The ^1^H NMR spectrum of **5** showed seven singlet methyls, and two pairs of *ortho*-coupled aromatic doublets [*δ*_H_ = 7.23, 7.15 (each 1H, d, *J* = 7.0 Hz)] and [*δ*_H_ = 7.48, 7.31 (each 1H, d, *J* = 8.0 Hz)] (Supplementary Table S2). On the basis of ^13^C NMR and HSQC spectra, compound **5** showed 28 carbons, including two carbonyl carbons (*δ*_C_ 173.2, 198.7) and sixteen aromatic carbons. From the 1D NMR data above, the presence of 1,2,3,4,5,8-hexasubstituted naphthalene and 1,2,3,4-tetrasubstituted benzene moieties were established. Further analysis, the HMBC correlations of H_3_-12/H_3_-13 with C-6/C-11; H_3_-14 with C-8/C-9/C-10; H_3_-15 with C-2/C-3/C-4, H_3_-12′/H_3_-13′ with C-6′/C-11′; H_3_-14′ with C-8′/C-9′/C-10′; and OH-1′ with C-1′/C-2′/C-10′ (Fig. 6A) indicated the presence of an aromatic cadinane and a dinorcadinane sesquiterpenoid unit, which further confirmed **5** was an aromatic dinor-cadinane-dimer. However, due to the crowded quaternary carbons, the connection of the aromatic cadinane and dinorcadinane sesquiterpenoid units could not be determined by NMR spectra to establish the complete structure of **5**. Finally, a single crystal suitable for X-ray analysis was obtained after careful recrystallization and subjected to an X-ray diffraction experiment to determine the structure of **5** as an unique 3′,4′-seco-3′,15′-dinor-cadinane-dimer (Fig. 6B). However, the optical rotation value of **5** was found to be zero indicating **5** was a racemic mixture, which was further confirmed by crystallographic data analysis^22^,^23^. Subsequent separation on a chiral preparative HPLC for two enantiomers was not successful, due to the reversible nature of hemiketal at the only chiral center C-1′^24^,^25^.

Involucratusin F (**6**) was isolated as yellowish amorphous powder. HRESIMS data (*m/z* 445.2011 [M+H]^+^, calcd for C_28_H_29_O_5_, 445.2010) gave the molecular formula C_28_H_28_O_5_ deducing **6** has a similar dinor-cadinane-dimer structure. This deduction was corroborated by the semblable NMR data of **5** and **6** (Supplementary Table S2). However, the C-4 [*δ*_H_ 7.50 (1H, s), *δ*_C_ 118.9] and C-11 [*δ*_H_ 3.64 (1H, m), *δ*_C_ 29.1] of **6** were different from the oxygenated quaternary carbons C-4 (*δ*_C_ 152.2) and C-11 (*δ*_C_ 92.5) of **5** indicating the ether bond between C-4 and C-11 of **5** have disappeared in **6**, which was supported by the HMBC correlations: H-4 with C-2/C-6/C-10/C-15; and H-11 with C-5/C-6/C-7/C-12/C-13 (Supplementary Fig. S1). Thus, the structure of **6** was also determined to be a novel 3′,4′-seco-3′,15′-dinor-cadinane-dimer (Fig. 1). The zero optical rotation value revealed **6** also was a racemic mixture.

Involucratusin G (**7**), with a molecular formula C_18_H_20_O_2_ deduced by its HRESIMS data (*m/z* 267.1390 [M−H]^−^, calcd for C_18_H_19_O_2_, 267.1391), was obtained as a yellowish oil. The 1D NMR spectra (Supplementary Table S3), with the help of HSQC spectrum, revealed **7** contained a 1,2,3,5-tetrasubstituted benzene ring [*δ*_H_ 6.57 (br s), 6.59 (br s); *δ*_C_ 115.9, 118.3, 123.2, 136.8, 143.0, 152.4] and a 1,2,4-trisubstituted benzene ring [*δ*_H_ 6.73 (br d, 9.0), 6.74 (br s), 7.97 (d, 9.0); *δ*_C_ 113.2, 116.0, 125.8, 127.9, 139.1, 154.4]. The presence of an isopropyl group were deduced by the characteristic ^1^H NMR signals [*δ*_H_ 1.41 (H-11, m); 0.77 (H_3_-12, d, 6.5); 0.87 (H_3_-13, d, 6.5)]. Further analysis, the three aforementioned substructures were incorporated with one methyl, one methylene and one methine groups to form the unique dodecanor-cadinane-dimer carbon skeleton of **7** according to the HMBC correlations of H-4′/H_3_-12′/H_3_-13′ with C-6′; H_2_-7′ with C-14/C-5′/C-8′/C-9′; H-14′ with C-10′; and H_3_-15′ with C-2′/C-3′/C-4′ (Supplementary Fig. S1). The oxygenated aromatic carbons C-9 (*δ*_C_ 154.4) and C-1′ (*δ*_C_ 152.4) and the remaining two oxygen and two hydrogen atoms in molecular formula indicated the presence of OH-9 and OH-1′. Thus, the planar structure of **7** was established as a novel dodecanor-cadinane-dimer (Fig. 1).

**Figure 7:**
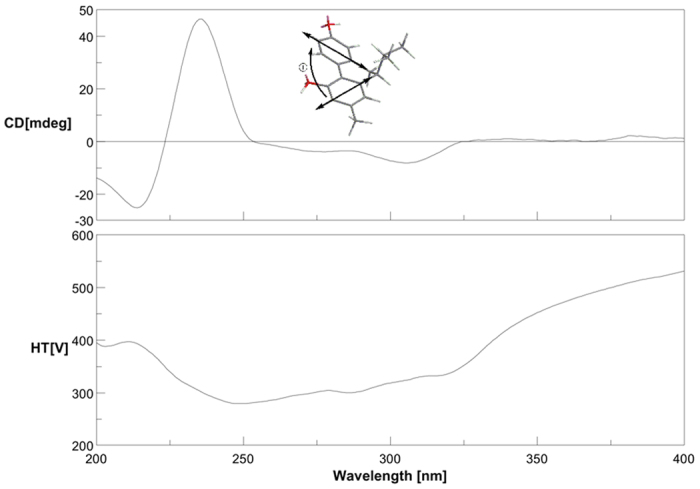
CD and exciton chirality sign of compound 7.

The absolute configuration of **7** was assigned by a CD exciton chirality method^19^,^20^,^21^. In the CD spectrum of **7** (Fig. 7), a positive Cotton effect at 235 nm (Δ*ε* +46.4) and a negative Cotton effect at 214 nm (Δ*ε* −25.2) due to the transition interaction between two identical phenyl chromophores indicated a positive chirality for **7**, which suggested the transition dipole moments of the two chromophores were oriented in a clockwise manner. Thus, the absolute configuration of **7** was established as 6′*S*.

Involucratusin H (**8**), which of molecular formula C_17_H_20_O_3_ was deduced by its HRESIMS data (*m/z* 273.1486 [M+H]^+^, calcd for C_17_H_21_O_3_, 273.1485), was obtained as yellowish needle crystals (MeOH:H_2_O = 10:1). The ^1^H NMR spectrum of **8** (Supplementary Table S3) gave altogether four methyls, one methoxyl, one phenolic hydroxyl, one aliphatic methine, and three aromatic methines [*δ*_H_ 7.14 (d, 7.5), 7.32 (s), 7.34 (d, 7.5)]. Its ^13^C NMR spectrum revealed a total of seventeen carbons including one ester carbonyl (*δ*_C_ 173.9) and ten aromatic carbons. In this molecule, the presence of 1,2,3,5,8-pentasubstituted naphthalene moiety was deduced based on the aforementioned three aromatic protons and ten aromatic carbons. From the HMBC spectrum (Supplementary Fig. S1), the correlations of H_3_-12/H_3_-13 with C-6/C-11; H_3_-14 with C-8/C-9/C-10; and H_3_-15 with C-2/C-3/C-4 were incorporated the naphthalene moiety deduced above to established the aromatic cadinane sesquiterpenoid unit. Furthermore, the HMBC correlations from the phenolic hydroxyl (*δ*_H_ 13.11) to C-2, C-10 and C-2′ (*δ*_C_ 173.9); and from the methoxyl (*δ*_H_ 3.99) to C-2′ deduced the phenolic hydroxyl linking at C-1 and the methyl ester group linking at C-2. To further confirm the deduced above, as showed in Supplementary Fig. S3, the single crystals of **8** were obtained and subjected to an X-ray diffraction experiment. Thus, the structure of **8** was doubtless determined as a novel tetradecanor-cadinane-dimer (Fig. 1).

### Biosynthetic pathway

**Figure 8:**
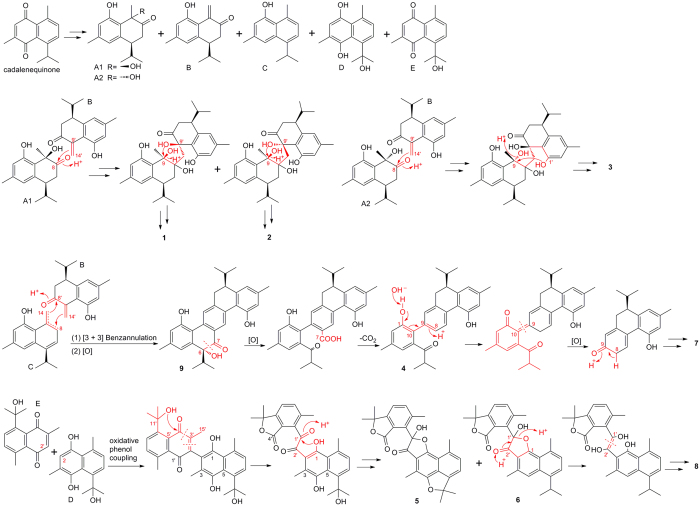
Plausible Biosynthetic Pathways for compounds 1–8.

Comparing with the reported cadinane dimers^1^,^4^, involucratusins A–H (**1**–**8**) represent five new carbon skeletons. The skeleton of **1**–**3** is corresponded to novel cadinane sesquiterpenoid dimer with unusual 1-oxaspiro[4.5]decane ring (**1** and **2**) or seven-membered cyclic ether ring (**3**), and new manners of bond connection (**1** and **2**: C-8 and C-14′, C-9 and oxygen on C-9′; **3**: C-8 and C-14′, C-9 and oxygen on C-1′). Although a series of cadinane dimers have been reported from natural sources, hitherto, only two nor-cadinane-dimers have been isolated^1^,^2^,^3^. Nor-cadinane-dimer (**4**), dinor-cadinane-dimer (**5** and **6**), dodecanor-cadinane-dimer (**7**), and tetradecanor-cadinane-dimer (**8**) represent four novel nor-cadinane-dimer carbon skeletons, which greatly enrich the structure of nor-cadinane-dimer. The novel and various backbones of **1**–**8** prompted us to speculate their biosynthetic pathways. As shown in Fig. 8, the pathways of **1**–**8** originating from cadalenequinone, which was considered as the biosynthetic monomeric precursor of involucratustones A–C^4^, were presumed, in which nucleophilic addition^9^,^26^, S_N_2 nucleophilic displacement^27^, [3 + 3] benzannulation^28^, oxidative cleavage^29^, decarboxylation^30^,^31^, oxidative phenol coupling^32^ were included.

From the viewpoint of biosynthesis, the most reported cadinane dimers are formed by the free-radical coupling reaction of two units of the corresponding monomers^1^. However, the backbone of involucratusins A–C (**1**–**3**) was proposed to be generated by the key nucleophilic addition reaction, which indicated **1**–**3** would provide new insight into the mechanisms of cadinane dimer biosynthesis. In the Fig. 8, it is revealed that the formation of nor-cadinane-dimer **4** is attributed to the decarboxylation reaction, which is the most common biogenetic pathway to form norterpenoids^2^,^9^,^33^,^34^,^35^. However, interesting, different from the most norterpeniods, the carbon skeletons of dinor-cadinane-dimers **5** and **6**, dodecanor-cadinane-dimer **7**, and tetradecanor-cadinane-dimer **8** were speculated to be produced by the key oxidative cleavage reaction. This indicated the elucidation of **5**–**8** would be a useful inspiration for the biosynthesis and total synthesis investigation of norterpenoids.

### MDR reversal activity screening

To search for effective MDR reversal agents, the MDR reversal activities of compound **1**, **2**, **4**, **5**, **7**–**9** were evaluated in MCF-7/DOX cells using the MTT method. The cytotoxicity assay exhibited that they were noncytotoxic to MCF-7/DOX cells, while they enhanced the cytotoxicity of DOX by 2.2–5.8-fold when incorporated at 10 *μ*M (Supplementary Table S4). Among them, compound **1** and **2** exhibited higher reversal fold (5.8 and 4.1, respectively), and **1** slightly higher than **2**. These results indicated that the characteristic 1-oxaspiro[4.5]decane ring of **1** and **2** might be a beneficial group for their MDR reversal activities, and the different configuration at spiro C-9′ of 1-oxaspiro[4.5]decane ring in **1** and **2** should slightly affect their activities. Cadinane dimers were reported to have a broad spectrum of biological activities such as male antifertility, anticancer, antiviral, and anti-inflammatory^1^. However, it is the first time that the MDR reversal activities of cadinane dimers have been reported. Therefore, the MDR reversal activities of the isolates would exploit new dimension for the bioactivity research of cadinane dimer.

In conclusion, the challenging structures of eight novel cadinane dimers and nor-cadinane-dimers, involucratusins A–H (**1**–**8**), from the rhizomes of *S. Involucratus* were established by a combination of spectroscopic data, CD experimentation, chemical reactions and single-crystal X-ray diffraction. Compounds **1**–**3** represent novel cadinane dimer backbone with new connection and unusual cores. Compounds **4**–**8** are a series of novel nor-cadinane-dimers representing four new carbon skeletons. Moreover, the key biogenetic reactions of cadinane dimers **1**–**3**, nucleophilic addition, and nor-cadinane-dimers **5**–**8**, oxidative cleavage, are very rare during the naturally formative process of cadinane dimers and norterpenoids, respectively. Therefore, the isolation of **1**–**8** has yielded advantageous illuminations for the research of cadinane dimer and norterpenoid biosynthesis. Biologically, the MDR reversal activities of **1**, **2**, **4**, **5**, **7**–**9** were evaluated in MCF-7/DOX cells. The results indicated that they enhanced the cytotoxicity of DOX by 2.2–5.8-fold when incorporated at 10 *μ*M. It is the first report about the MDR reversal activity of cadinane dimers. Thus, the MDR reversal activities of the isolates would expand new dimension for the bioactivity research of cadinane dimer, and provide valuable inspiration for the MDR reversal drug discovery.

## Methods

### General experimental procedures

Melting point was measured on an X-4 digital display micro-melting apparatus, uncorrected. Optical rotations were determined with a JASCO P-1020 polarimeter. UV spectra were performed on a Shimadzu UV-2450 spectrophotometer. CD spectrum was measured on a JASCO 810 spectropolarimeter. 1D and 2D NMR spectra were acquired on a Bruker AV-500 NMR instrument at 500 MHz (^1^H) and 125 MHz (^13^C) in CDCl_3_ and DMSO-*d*_6_. ESI and HRESI mass spectra were recorded on an Agilent 1100 series LC-MSD-Trap-SL mass analyzer and an Agilent 6520B Q-TOF mass instrument, respectively. Column chromatography (CC) was done with Silica gel (Qingdao Haiyang Chemical Co., Ltd., Qingdao, China), ODS (40–63 *μ*m, FuJi, Japan), Sephadex LH-20 (Pharmacia, Sweden). Preparative HPLC was carried out using an SHMADZU LC-6AD series instrument with a Shim-park RP-C18 column (20 × 200 mm) and a SHMADZU SPD-20A detector.

### Plant material

The rhizomes of *S. involucratus* were collected from Guangxi province of China in November 2012, and were authenticated by Professor Min-Jian Qin, Department of Medicinal Plants, China Pharmaceutical University. A voucher specimen (No. 201211-SI) is deposited in the Department of Natural Medicinal Chemistry, China Pharmaceutical University.

### Extraction and isolation

The air-dried rhizomes of *S. involucratus* (5 kg) were exhaustively extracted with 95% EtOH at room temperature (5 × 1 d). The residue (200 g) obtained by concentrating the EtOH extract under reduced pressure was suspended in H_2_O (1.5 L), then partitioned successively with petroleum ether (PE) (4 × 2 L), dichloromethane (CH_2_Cl_2_) (4 × 2 L) and ethyl acetate (EtOAc) (4 × 2 L). The PE, CH_2_Cl_2_ and EtOAc extracts yielded 120 g, 30 g and 4 g after removal of the solvent, respectively. The CH_2_Cl_2_ extract was chromatographed on a silica gel column using a gradient of PE-acetone (10:1, 5:1, 2:1, 1:1) to afford four fractions (Fr. 1–4) collected and pooled on the basis of TLC analysis. Fr. 1 (4.7 g) was run on ODS column chromatography with MeOH-H_2_O (50–100%), to obtain eight fractions (Fr. 1.1–Fr. 1.8). Fr. 1.3 was then chromatographed on an ODS column using a step gradient of MeOH-H_2_O (60–100%), to give sixteen fractions (Fr. 1.3.1–1.3.16). Fr. 1.3.8 was further purified on preparative HPLC using MeOH-H_2_O (80:20, 10 mL/min) to obtain **2** (31 mg) and **6** (2 mg). Fr. 1.3.13 was recrystallized with MeOH/H_2_O (10:1), yielding **8** (2 mg). Fr. 2 (9.3 g) was run on ODS column chromatography with MeOH-H_2_O (30–100%), to obtain five fractions (Fr. 2.1–Fr. 2.5). Fr. 2.2 was performed on a silica gel column chromatography using a gradient of PE-acetone (10:1, 5:1, 2:1) to afford seven fractions (Fr. 2.2.1–2.2.7). Fr. 2.2.2 was then chromatographed on an ODS column using a step gradient of MeOH-H_2_O (50–100%), to give twelve fractions (Fr. 2.2.2.1–2.2.2.12). Fr. 2.2.2.5 was run on Sephadex LH-20 with MeOH, to obtain eight subfractions (Fr. 2.2.2.5.1–2.2.2.5.8). Fr. 2.2.2.5.1 was finally purified on preparative HPLC using MeOH-H_2_O (85:15, 10 mL/min) to obtain **3** (4 mg). Fr. 2.2.2.5.2 was recrystallized with MeOH/H_2_O (10:1) to yield **1** (50 mg). Fr. 2.2.2.6 was purified by Sephadex LH-20 column chromatography and recrystallized with MeOH/H_2_O (10:1) to obtain **5** (18 mg). After chromatographed on an ODS column using a step gradient of MeOH-H_2_O (65–100%), Fr. 2.2.4 was separated to give twelve fractions (Fr. 2.2.4.1–2.2.4.12). Fr. 2.2.4.2 was purified by Sephadex LH-20 column chromatography and preparative HPLC (MeOH/H_2_O = 80:20, v/v) to obtain **4** (6 mg), **7** (2 mg) and **9** (100 mg).

### Spectroscopic data

Involucratusin A (**1**): colorless needle crystals (MeOH/H_2_O, 10:1); mp 187–190 °C; [*α*]_D_^25^ +82.0 (*c* 0.2, MeOH); UV (MeOH) (log *ɛ*) *λ*_max_: 207 (4.71) nm, 283 (3.64) nm; CD (MeOH): *λ* (*ɛ*) = 207 (2.26), 219 (13.88), 277 (−3.48), 301 (−3.40) nm; for ^1^H and ^13^C NMR data, see Supplementary Table S1; ESIMS *m/z* 513.2 [M+Cl]^−^; HRESIMS *m/z* 501.2613 [M+Na]^+^ (calcd for C_30_H_38_O_5_Na, 501.2611).

Involucratusin B (**2**): white amorphous powder; [*α*]_D_^25^−5.4 (*c* 0.1, MeOH); UV (MeOH) (log *ɛ*) *λ*_max_: 205 (4.49) nm, 281 (3.68) nm; CD (MeOH): *λ* (*ɛ*) = 200 (8.38), 218 (−0.01), 257 (0.33), 283 (−1.16) nm; for ^1^H and ^13^C NMR data, see Supplementary Table S1; ESIMS *m/z* 477.2 [M−H]^−^; HRESIMS *m/z* 501.2609 [M+Na]^+^ (calcd for C_30_H_38_O_5_Na, 501.2611).

Involucratusin C (**3**): yellowish oil; [*α*]_D_^25^ +138.0 (*c* 0.2, MeOH); UV (MeOH) (log *ɛ*) *λ*_max_: 205 (4.27) nm, 283 (3.39) nm; CD (MeOH): *λ* (*ɛ*) = 209 (29.72), 230 (15.52), 254 (0.33), 282 (3.96) nm; for ^1^H and ^13^C NMR data, see Supplementary Table S1; ESIMS *m/z* 513.3 [M+Cl]^−^; HRESIMS *m/z* 501.2610 [M+Na]^+^ (calcd for C_30_H_38_O_5_Na, 501.2611).

Involucratusin D (**4**): yellowish oil; [*α*]_D_^25^ −15.3 (*c* 0.18, MeOH); UV (MeOH) (log *ɛ*) *λ*_max_: 213 (4.35) nm, 260 (3.84) nm, 282 (3.88) nm, 316 (3.98) nm; CD (MeOH): *λ* (*ɛ*) = 210 (−18.57), 237 (35.11), 275 (−8.30), 312 (−7.52) nm; for ^1^H and ^13^C NMR data, see Supplementary Table S2; ESIMS *m/z* 427.4 [M−H]^−^; HRESIMS *m/z* 451.2245 [M+Na]^+^ (calcd for C_29_H_32_O_3_Na, 451.2244).

Involucratusin E (**5**): yellowish needle crystals (MeOH/H_2_O, 10:1); mp 256–260 °C; [*α*]_D_^25^ 0 (*c* 0.2, MeOH); UV (MeOH) (log *ɛ*) *λ*_max_: 206 (4.59) nm, 222 (4.61) nm, 280 (4.38) nm, 329 (3.51) nm, 434 (3.73) nm; for ^1^H and ^13^C NMR data, see Supplementary Table S2; ESIMS *m/z* 457.1 [M−H]^−^; HRESIMS *m/z* 459.1804 [M+H]^+^ (calcd for C_28_H_27_O_6_, 459.1802).

Involucratusin F (**6**): yellowish amorphous powder; [*α*]_D_^25^ 0 (*c* 0.1, MeOH); UV (MeOH) (log *ɛ*) *λ*_max_: 206 (4.48) nm, 266 (4.45) nm, 290 (3.85) nm, 397 (3.52) nm; for ^1^H and ^13^C NMR data, see Supplementary Table S2; ESIMS *m/z* 445.2 [M+H]^+^; HRESIMS *m/z* 445.2011 [M+H]^+^ (calcd for C_28_H_29_O_5_, 445.2010).

Involucratusin G (**7**): yellowish oil; [*α*]_D_^25^ 37.8 (*c* 0.1, MeOH); UV (MeOH) (log *ɛ*) *λ*_max_: 213 (3.97) nm, 278 (3.67) nm, 298 (3.55) nm; CD (MeOH): *λ* (*ɛ*) = 214 (−25.28), 235 (46.47), 305 (−8.15) nm; for ^1^H and ^13^C NMR data, see Supplementary Table S3; HRESIMS *m/z* 267.1390 [M−H]^−^ (calcd for C_18_H_19_O_2_, 267.1391).

Involucratusin H (**8**): yellowish needle crystals (MeOH/H_2_O, 10:1); mp 132–134 °C; UV (MeOH) (log *ɛ*) *λ*_max_: 219 (4.04) nm, 252 (4.22) nm, 285 (3.35) nm, 296 (3.32) nm, 364 (3.37) nm; for ^1^H and ^13^C NMR data, see Supplementary Table S3; HRESIMS *m/z* 273.1486 [M+H]^+^ (calcd for C_17_H_21_O_3_, 273.1485).

### X-ray crystallographic analysis

Crystal data were obtained on a Bruker Smart-1000 CCD with a graphite monochromator with Cu K*α* radiation (*λ* = 1.54184 Å) at 291(2) K. The structures were solved by direct methods using the SHELXS-97 and expanded using difference Fourier techniques, refined with the SHELXL-97. Colorless needle crystals of **1** were obtained from MeOH/H_2_O (10:1), two molecules of H_2_O was resolved together with **1** in the crystal structure. Crystal data of **1**: C_30_H_42_O_7_ (M = 514.63); orthorhombic crystal (0.36 × 0.32 × 0.25 mm); space group C2; unit cell dimensions *a* = 25.8671(4) Å, *b* = 8.8460(2) Å, *c* = 13.4719(2) Å, *V* = 3082.13(10) Å3; *Z* = 4; *D*_calcd_ = 1.109 mg/mm^3^; *μ* = 0.630 mm^−1^; 11127 reflections measured (6.836 ≤ 2*Θ* ≤ 139.05); 4849 unique (R_int_ = 0.0226) which were used in all calculations; the final refinement gave R_1_ = 0.0502 (>2 *σ(I)*) and wR2 = 0.1435 (all data); Flack parameter = −0.03(9). Crystallographic data for **1** have been deposited in the Cambridge Crystallographic Data Centre (deposition number: CDCC 1455606).

Yellowish needle crystals of **5** were obtained from MeOH/H_2_O (10:1). Crystal data of **5**: C_28_H_26_O_6_ (M = 458.49); orthorhombic crystal (0.35 × 0.3 × 0.26 mm); space group Pbca; unit cell dimensions *a* = 11.94950(10) Å, *b* = 13.38660(10) Å, *c* = 30.6047(2) Å, *V* = 4895.62(6) Å3; *Z* = 8; *D*_calcd_ = 1.244 mg/mm^3^; *μ* = 0.712 mm^−1^; 37529 reflections measured (10.366 ≤ 2*Θ* ≤ 139.804); 4609 unique (R_int_ = 0.0227) which were used in all calculations; the final refinement gave R_1_ = 0.0376 (>2 *σ(I)*) and wR2 = 0.1082 (all data). Crystallographic data for **5** have been deposited in the Cambridge Crystallographic Data Centre (deposition number: CDCC 1455607).

Yellowish needle crystals of **8** were obtained from MeOH/H_2_O (10:1). Crystal data of **8**: C_17_H_20_O_3_ (M = 272.33); orthorhombic crystal (0.36 × 0.32 × 0.3 mm); space group Pna2_1_; unit cell dimensions *a* = 19.9103(7) Å, *b* = 10.4660(3) Å, *c* = 7.1348(3) Å, *V* = 1486.76(9) Å3; *Z* = 4; *D*_calcd_ = 1.217 mg/mm^3^; *μ* = 0.660 mm^−1^; 9807 reflections measured (8.882 ≤ 2*Θ* ≤ 138.982); 2441 unique (R_int_ = 0.0236) which were used in all calculations; the final refinement gave R_1_ = 0.0547 (>2 *σ(I)*) and wR2 = 0.1710 (all data); Flack parameter = −0.3(8). Crystallographic data for **8** have been deposited in the Cambridge Crystallographic Data Centre (deposition number: CDCC 1455608).

### Reduction of compound 2 to yield 2a

Approximately 10.0 mg **2** and 10.0 mg NaBH_4_ were mixed in 4 ml MeOH at room temperature for 1 h. Further 0.5 ml 0.1 mol/L HCl was added to the reaction mixture for 20 min. The reaction mixture was partitioned with EtOAc (3 × 10 mL), and the organic phase was evaporated and purified by Pre-HPLC using ACN-H_2_O (75:25, 10 mL/min) to afford **2a** (8 mg).

### *Bis*-MTPA esters of compound 2a

A 6 *μ*L portion of (*S*)-MTPACl was added to a solution of **2a** (2 mg) in freshly distilled dry pyridine (0.2 mL), and the mixture was kept at room temperature for 2 h. The reaction mixture was diluted with MeOH, and purified by preparative HPLC using MeOH-H_2_O (90:10, 10 mL/min) to afford the bis-(*R*)-MTPA ester **2aa** (1.7 mg). In the same manner, compound **2a** (2 mg) was treated with (*R*)-MTPACl to give the bis-(*S*)-MTPA ester **2ab** (1.6 mg).

### *Bis*-MTPA esters of compound 3

Compound **3** (1.5 mg) was dissolved in freshly distilled dry pyridine (0.2 mL), and 6 *μ*L portion of (*S*)-MTPACl was added. After kept for 2 h at room temperature, the reaction mixture was diluted with MeOH, and purified by preparative HPLC using MeOH-H_2_O (90:10, 10 mL/min) to afford the (*R*)-MTPA ester **3a** (1.2 mg). *S*-MTPA ester **3b** (1.2 mg) was prepared with (*R*)-MTPACl in the same manner.

### Cytotoxicity assays

MCF-7/DOX cells were cultured in DMEM medium with 10% fetal bovine serum, harvested with trypsin, and resuspended in a final concentration of 4.5 × 10^4^ cells/mL. Aliquots (0.1 mL) of cell suspension were seeded evenly into 96-well culture multiplates and incubated in a 37 °C incubator containing 5% CO_2_ for 24 h. A series of concentrations for the isolates in DMSO were added to designated wells. After 48 h, an MTT assay was performed as described previously^36^.

### MDR reversal assays

MCF-7/DOX cells were distributed into 96-well culture plates at 4.5 × 10^3^ cells per well. A full range of concentrations of DOX with or without 10 *μ*M samples or 10 *μ*M Verapamil (positive control) were added to the cells. After 48 h, the MTT assay was performed as described above. IC_50_ values of DOX were calculated from plotted results using untreated cells as 100%. The reversal fold, in terms of potency of reversal, was calculated using the following formula: reversal fold (RF) = IC_50_ (MCF-7/DOX cells)/IC_50_ (MCF-7/DOX cells combined with sample treatment). All assays were performed in triplicate.

## Additional Information

**How to cite this article**: Li, Q.-M. *et al*. Involucratusins A–H: Unusual Cadinane Dimers from *Stahlianthus involucratus* with Multidrug Resistance Reversal Activity. *Sci. Rep.* **6**, 29744; doi: 10.1038/srep29744 (2016).

## Supplementary Material

Supplementary Information
