# Supplementary material for: Involucratusins A–H: Unusual Cadinane Dimers from Stahlianthus involucratus with Multidrug Resistance Reversal Activity
Source: Sci Rep. 2016 Jul 13;6:29744. doi: 10.1038/srep29744 (PMC4942826; doi:10.1038/srep29744)
Supplement: Supplementary Information [file srep29744-s1.pdf]

Supporting Information

Involucratusins A-H: Unusual Cadinane Dimers from *Stahlianthus involucratus* with Multidrug Resistance Reversal Activity

Qiang-Ming Li[+], Jian-Guang Luo[+], Rui-Zhi Wang, Xiao-Bing Wang, Ming-Hua Yang, Jun Luo & Ling-Yi Kong[*]

State Key Laboratory of Natural Medicines, Department of Natural Medicinal Chemistry, China Pharmaceutical University, 24 Tong Jia Xiang, Nanjing 210009, People's Republic of China

* Corresponding author. Tel/Fax: +86-25-83271405. E-mail: cpu_lykong@126.com

[+] These authors contributed equally to this work.

Table of Contents

Table S1. NMR Data of compounds **1**, **2**, **2a** and **3** S5
Table S2. NMR Data of compounds **4**, **5** and **6**.......... S6
Table S3. NMR Data of compounds **7** and **8** S7
Table S4. Multidrug resistance reversal effects of the isolates on MCF-7/DOX S8
Figure S1. Key HMBC and ^1H-^1H COSY correlations of compounds **1**, **2**, **2a**, **3-8** S9
Figure S2. Key ROESY correlations of compounds **1**, **2**, **2a** and **3** S10
Figure S3. X-ray structure of compound **8** S11

Figure S4. ^1H NMR spectrum of involucratusin A (**1**) in $CDCl_3$ S12
Figure S5. ^{13}C NMR spectrum of involucratusin A (**1**) in $CDCl_3$ S13
Figure S6. COSY spectrum of involucratusin A (**1**) in $CDCl_3$ S14
Figure S7. HSQC spectrum of involucratusin A (**1**) in $CDCl_3$ S15
Figure S8. HMBC spectrum of involucratusin A (**1**) in $CDCl_3$ S16
Figure S9. ROESY spectrum of involucratusin A (**1**) in $CDCl_3$ S17
Figure S10. ESIMS spectrum of involucratusin A (**1**) in CH_3OH S18
Figure S11. HRESIMS spectrum of involucratusin A (**1**) in CH_3OH S19
Figure S12. UV spectrum of involucratusin A (**1**) in CH_3OH S20

Figure S13. ^1H NMR spectrum of involucratusin B (**2**) in $CDCl_3$ S21
Figure S14. ^{13}C NMR spectrum of involucratusin B (**2**) in $CDCl_3$ S22
Figure S15. HSQC spectrum of involucratusin B (**2**) in $CDCl_3$ S23
Figure S16. HMBC spectrum of involucratusin B (**2**) in $CDCl_3$ S24
Figure S17. ROESY spectrum of involucratusin B (**2**) in $CDCl_3$ S25
Figure S18. ESIMS spectrum of involucratusin B (**2**) in CH_3OH S26
Figure S19. HRESIMS spectrum of involucratusin B (**2**) in CH_3OH S27
Figure S20. UV spectrum of involucratusin B (**2**) in CH_3OH S28

Figure S21. ^1H NMR spectrum of compound **2a** in $CDCl_3$ S29
Figure S22. ^{13}C NMR spectrum of compound **2a** in $CDCl_3$ S30
Figure S23. HSQC spectrum of compound **2a** in $CDCl_3$ S31
Figure S24. HMBC spectrum of compound **2a** in $CDCl_3$ S32
Figure S25. ROESY spectrum of compound **2a** in $CDCl_3$ S33
Figure S26. ESIMS spectrum of compound **2a** in CH_3OH S34
Figure S27. HRESIMS spectrum of compound **2a** in CH_3OH S35
Figure S28. ^1H NMR spectrum of compound **2aa** in $CDCl_3$ S36
Figure S29. ESIMS spectrum of compound **2aa** in CH_3OH S37
Figure S30. HRESIMS spectrum of compound **2aa** in CH_3OH S38
Figure S31. ^1H NMR spectrum of compound **2ab** in $CDCl_3$ S39
Figure S32. ESIMS spectrum of compound **2ab** in CH_3OH S40
Figure S33. HRESIMS spectrum of compound **2ab** in CH_3OH S41

Figure S34. ^{1}H NMR spectrum of involucratusin C (**3**) in $CDCl_3$ S42
Figure S35. ^{13}C NMR spectrum of involucratusin C (**3**) in $CDCl_3$ S43
Figure S36. HSQC spectrum of involucratusin C (**3**) in $CDCl_3$ S44
Figure S37. HMBC spectrum of involucratusin C (**3**) in $CDCl_3$ S45
Figure S38. ROESY spectrum of involucratusin C (**3**) in $CDCl_3$ S46
Figure S39. ^{1}H NMR spectrum of involucratusin C (**3**) in DMSO-d_6 .... S47
Figure S40. ^{13}C NMR spectrum of involucratusin C (**3**) in DMSO-d_6 .... S48
Figure S41. HSQC spectrum of involucratusin C (**3**) in DMSO-d_6 .... S49
Figure S42. HMBC spectrum of involucratusin C (**3**) in DMSO-d_6 .... S50
Figure S43. ROESY spectrum of involucratusin C (**3**) in DMSO-d_6 .... S51
Figure S44. ESIMS spectrum of involucratusin C (**3**) in CH_3OH S52
Figure S45. HRESIMS spectrum of involucratusin C (**3**) in CH_3OH S53
Figure S46. UV spectrum of involucratusin C (**3**) in CH_3OH S54
Figure S47. ^{1}H NMR spectrum of compound **3a** in $CDCl_3$ S55
Figure S48. ESIMS spectrum of compound **3a** in CH_3OH S56
Figure S49. HRESIMS spectrum of compound **3a** in CH_3OH S57
Figure S50. ^{1}H NMR spectrum of compound **3b** in $CDCl_3$ S58
Figure S51. ESIMS spectrum of compound **3b** in CH_3OH S59
Figure S52. HRESIMS spectrum of compound **3b** in CH_3OH S60

Figure S53. ^{1}H NMR spectrum of involucratusin D (**4**) in $CDCl_3$ S61
Figure S54. ^{13}C NMR spectrum of involucratusin D (**4**) in $CDCl_3$ S62
Figure S55. DEPT spectrum of involucratusin D (**4**) in $CDCl_3$ S63
Figure S56. COSY spectrum of involucratusin D (**4**) in $CDCl_3$ S64
Figure S57. HSQC spectrum of involucratusin D (**4**) in $CDCl_3$ S65
Figure S58. HMBC spectrum of involucratusin D (**4**) in $CDCl_3$ S66
Figure S59. ROESY spectrum of involucratusin D (**4**) in $CDCl_3$ S67
Figure S60. ESIMS spectrum of involucratusin D (**4**) in CH_3OH S68
Figure S61. HRESIMS spectrum of involucratusin D (**4**) in CH_3OH S69
Figure S62. UV spectrum of involucratusin D (**4**) in CH_3OH S70

Figure S63. ^{1}H NMR spectrum of involucratusin E (**5**) in $CDCl_3$ S71
Figure S64. ^{13}C NMR spectrum of involucratusin E (**5**) in $CDCl_3$ S72
Figure S65. HSQC spectrum of involucratusin E (**5**) in $CDCl_3$ S73
Figure S66. HMBC spectrum of involucratusin E (**5**) in $CDCl_3$ S74
Figure S67. ROESY spectrum of involucratusin E (**5**) in $CDCl_3$ S75
Figure S68. ESIMS spectrum of involucratusin E (**5**) in CH_3OH S76
Figure S69. HRESIMS spectrum of involucratusin E (**5**) in CH_3OH S77
Figure S70. UV spectrum of involucratusin E (**5**) in CH_3OH S78

Figure S71. ^{1}H NMR spectrum of involucratusin F (**6**) in $CDCl_3$ S79
Figure S72. ^{13}C NMR spectrum of involucratusin F (**6**) in $CDCl_3$ S80
Figure S73. HSQC spectrum of involucratusin F (**6**) in $CDCl_3$ S81
Figure S74. HMBC spectrum of involucratusin F (**6**) in $CDCl_3$ S82

Figure S75. ROESY spectrum of involucratusin F (**6**) in $CDCl_3$ S83
Figure S76. ESIMS spectrum of involucratusin F (**6**) in CH_3OH S84
Figure S77. HRESIMS spectrum of involucratusin F (**6**) in CH_3OH S85
Figure S78. UV spectrum of involucratusin F (**6**) in CH_3OH S86

Figure S79. ^{1}H NMR spectrum of involucratusin G (**7**) in $CDCl_3$ S87
Figure S80. ^{13}C NMR spectrum of involucratusin G (**7**) in $CDCl_3$ S88
Figure S81. HSQC spectrum of involucratusin G (**7**) in $CDCl_3$ S89
Figure S82. HMBC spectrum of involucratusin G (**7**) in $CDCl_3$ S90
Figure S83. HRESIMS spectrum of involucratusin G (**7**) in CH_3OH S91
Figure S84. UV spectrum of involucratusin G (**7**) in CH_3OH S92

Figure S85. ^{1}H NMR spectrum of involucratusin H (**8**) in $CDCl_3$ S93
Figure S86. ^{13}C NMR spectrum of involucratusin H (**8**) in $CDCl_3$ S94
Figure S87. HSQC spectrum of involucratusin H (**8**) in $CDCl_3$ S95
Figure S88. HMBC spectrum of involucratusin H (**8**) in $CDCl_3$ S96
Figure S89. HRESIMS spectrum of involucratusin H (**8**) in CH_3OH S97
Figure S90. UV spectrum of involucratusin H (**8**) in CH_3OH S98

Table S1. NMR Data of compounds 1, 2, 2a and 3

No.	**1**		**2**		**2a**		**3**			
	δ_H[ab]	δ_C[b]	δ_H[ab]	δ_C[b]	δ_H[ab]	δ_C[b]	δ_H[ab]	δ_C[b]	δ_H[ac]	δ_C[c]
1		155.3		154.7		154.6		155.8		155.1
2	6.69 (br s)	117.1	6.65 (br s)	117.4	6.69 (br s)	117.8	6.69 (br s)	116.8	6.56 (br s)	115.5
3		137.7		138.9		139.2		138.7		137.1
4	6.40 (br s)	123.4	6.46 (br s)	123.7	6.47 (br s)	124.0	6.38 (br s)	122.8	6.34 (br s)	122.1
5		136.4		138.6		138.6		136.9		137.2
6	2.45 (dd, 9.0, 4.0)	52.9	2.55 (dd, 9.0, 4.0)	53.6	2.55 (dd, 9.0, 4.0)	53.6	2.79 (dd, 5.5 2.0)	51.7	2.65 (br d, 5.5)	50.8
7	3.79 (dd, 11.0, 4.0)	73.4	4.17 (dd, 10.0, 4.0)	76.8	4.14 (ddd, 10.0, 4.0, 4.0)	77.1	4.09 (dd, 11.0, 5.5)	70.7	3.81 (ddd, 11.0, 5.5, 5.5)	68.4
8	2.81 (br dd, 11.0, 10.0)	50.0	3.10 (ddd, 10.0, 10.0, 6.5)	51.6	2.90 (ddd, 10.0, 9.5, 7.0)	50.5	2.24 (ddd, 11.5, 11.0, 3.5)	40.9	2.00 (ddd, 12.5, 11.0, 3.5)	40.3
9		90.4		90.1		87.2		87.4		86.0
10		123.2		122.3		121.5		122.8		122.9
11	1.95 (m)	27.3	2.01 (m)	27.7	1.98 (m)	27.9	2.00 (m)	26.7	1.95 (m)	25.4
12	1.22 (d, 6.5)	24.5	1.25 (d, 6.5)	24.6	1.26 (d, 6.5)	24.6	0.98 (d, 7.0)	26.2	0.90 (d, 7.0)	25.7
13	0.79 (d, 6.5)	23.4	0.74 (d, 6.5)	23.3	0.75 (d, 6.5)	23.3	0.13 (d, 7.0)	20.8	0.01 (d, 7.0)	20.3
14	1.92 (s)	31.1	1.70 (s)	29.9	1.87 (s)	32.2	1.87 (s)	23.7	1.70 (s)	23.1
15	2.27 (s)	21.3	2.25 (s)	21.3	2.26 (s)	21.4	2.27 (s)	21.3	2.20 (s)	20.5
1'		155.8		155.5		155.9		152.2		152.0
2'	6.66 (br s)	117.2	6.50 (br s)	117.7	6.45 (br s)	116.6	6.61 (br s)	122.3	6.39 (br s)	120.9
3'		139.8		139.4		139.3		139.7		137.7
4'	6.55 (br s)	121.6	6.54 (br s)	122.9	6.70 (br s)	120.0	6.73 (br s)	127.0	6.79 (br s)	126.0
5'		140.4		140.8		140.9		139.4		139.7
6'	2.96[d]	47.0	2.79 (ddd, 9.0, 6.0, 4.0)	47.4	3.09 (ddd, 10.5, 5.5, 5.0)	36.3	2.89 (ddd, 7.5, 6.0, 2.0)	48.9	2.84 (ddd, 8.0, 5.5, 2.5)	47.1
7'a	2.95[d]	38.1	2.92 (dd, 13.5, 6.0)	40.2	2.17 (br dd, 12.5, 5.0)	25.0	3.06 (dd, 13.5, 6.0)	38.8	2.93 (dd, 13.5, 5.5)	39.1
7'b	2.51 (dd, 13.0, 3.0)		2.86 (dd, 13.5, 3.5)		1.72 (br dd, 12.5, 5.5)		2.83 (dd, 13.5, 2.0)		2.61 (dd, 13.5, 2.5)	
8'		207.3		209.6	4.23 (br d, 3.0)	71.3		211.6		210.6
9'		91.4		85.9		89.5		76.1		75.0
10'		119.9		121.2		119.4		128.8		129.6
11'	1.84 (m)	32.8	1.58 (m)	33.9	2.52 (m)	30.7	1.62 (m)	34.6	1.50 (m)	33.1
12'	0.87 (d, 6.5)	21.5	0.90 (d, 7.0)	21.7	1.05 (d, 7.0)	20.7	0.86 (d, 7.0)	21.4	0.80 (d, 6.5)	20.9
13'	0.77 (d, 6.5)	18.6	0.93 (d, 7.0)	19.7	0.58 (d, 7.0)	15.6	0.88 (d, 7.0)	19.7	0.84 (d, 6.5)	19.1
14'a	2.85 (dd, 13.5, 10.0)	40.7	3.00 (dd, 13.0, 10.0)	43.1	2.72 (dd, 14.0, 9.5)	44.0	2.27[b]	34.9	2.10 (dd, 15.5, 3.5)	33.2
14'b	2.67 (dd, 13.5, 1.5)		2.40 (dd, 13.5, 6.5)		2.34 (dd, 14.0, 7.0)		1.91 (dd, 15.0, 11.5)		1.75 (dd, 15.5, 12.5)	
15'	2.29 (s)	21.4	2.23 (s)	21.2	2.21 (s)	21.3	2.18 (s)	21.1	2.13 (s)	20.2
OH-1					6.97 (br s)				8.90 (br s)	
OH-7									4.95 (d, 5.5)	
OH-1'					6.91 (br s)					
OH-9'									5.26 (s)	

[a] Figures in parentheses are coupling constants in hertz. [b] Measured in $CDCl_3$. [c] Measured in DMSO-d_6. [d] Multiplicity patterns were unclear due to signal overlapping.

Table S2. NMR Data of compounds 4, 5 and 6 (in $CDCl_3$)

No.	4		5		6	
	δ_H[a]	δ_C	δ_H[a]	δ_C	δ_H[a]	δ_C
1		152.8		166.4		174.1
2	6.91 (br s)	118.1		116.4		113.4
3		139.5		107.4		133.4
4	6.81 (br s)	120.7		152.2	7.50 (s)	118.9
5		142.2		133.8		138.7
6		211.7		144.6		142.3
7			7.15 (d, 7.0)	119.8	7.44 (d, 7.5)	126.8
8	7.20 (br d, 8.0)	128.3	7.23 (d, 7.0)	129.4	7.18 (d, 7.5)	128.1
9		132.4		134.1		134.6
10		122.6		117.9		120.7
11	2.48 (m)	40.1		92.5	3.64 (m)	29.1
12	0.86 (d, 7.0)	18.8	1.71 (s)	28.9	1.38 (d, 7.0)	23.7
13	0.81 (d, 7,0)	18.5	1.68 (s)	28.7	1.37 (d, 7.0)	23.6
14	7.15 (br s)	130.2	2.80 (s)	20.9	2.83 (s)	23.5
15	2.37 (s)	21.4	2.55 (s)	10.4	2.74 (s)	18.8
1'		153.2		103.6		102.9
2'	6.59 (br s)	116.1		198.7		197.8
3'		138.3				
4'	6.62 (br s)	123.4		173.2		173.2
5'		143.8		125.2		125.3
6'	2.34 (m)	46.8		155.7		155.7
7'	2.95 (dd, 15.0, 4.5)<br>2.90 (dd, 15.0, 3.0)	33.1	7.31 (d, 8.0)	121.2	7.32 (d, 8.0)	121.3
8'		138.2	7.48 (d, 8.0)	139.2	7.48 (d, 8.0)	139.2
9'		133.4		138.8		138.6
10'		117.7		137.9		137.8
11'	1.38 (m)	28.6		86.6		86.7
12'	0.78 (d, 6.5)	22.0	1.72 (s)	27.5	1.73 (s)	27.6
13'	0.85 (d, 6.5)	20.8	1.71 (s)	27.4	1.72 (s)	27.4
14'	8.28 (br d, 8.0)	128.2	2.25 (s)	22.6	2.20 (s)	22.6
15'	2.31 (s)	21.3				
1'-OH			10.19 (s)		10.25 (s)	

[a] Figures in parentheses are coupling constants in hertz. [b] Multiplicity patterns were unclear due to signal overlapping

Table S3. NMR Data of compounds 7 and 8 (in $CDCl_3$)

No.	**7**		**8**	
	δ_H[a]	δ_C	δ_H[a]	δ_C
1				166.4
2				106.3
3				134.3
4			7.32 (s)	116.9
5				135.7
6				141.3
7			7.34 (d, 7.5)	125.5
8	6.73 (br d, 9.0)	113.2	7.14 (d, 7.5)	128.4
9		154.4		136.1
10				123.7
11			3.59 (m)	28.5
12			1.34 (d, 7.0)	23.7
13			1.34 (d, 7.0)	23.7
14	6.74 (br s)	116.0	2.92 (s)	25.7
15			2.64 (s)	25.2
1'		152.4		
2'	6.57 (br s)	115.9		173.9
3'		136.8		
4'	6.59 (br s)	123.2		
5'		143.0		
6'		47.0		
7'a	2.88 (dd, 15.0, 4.5)	33.3		
7'b	2.83 (dd, 15.0, 3.0)			
8'		139.1		
9'		125.8		
10'		118.3		
11'	1,41 (m)	28.3		
12'	0.77 (d, 6.5)	22.0		
13'	0.87 (d, 6.5)	20.8		
14'	7.97 (d, 9.0)	127.9		
15'	2.29 (s)	21.3		
1-OH			13.1 (s)	
2'-OMe			3.99 (s)	52.2

[a] Figures in parentheses are coupling constants in hertz.

Table S4. Multidrug resistance reversal effects of the isolates on MCF-7/DOX

Sample	IC_{50} (μM)	RF Value
DOX + **1**	10.35 ± 0.24	5.8
DOX + **2**	14.65 ± 0.37	4.1
DOX + **4**	26.10 ± 0.61	2.3
DOX + **5**	27.68 ± 0.52	2.2
DOX + **7**	23.93 ± 0.47	2.5
DOX + **8**	20.05 ± 0.33	3.0
DOX + **9**	15.80 ± 0.31	3.8
DOX + Ver	4.32 ± 0.29	13.9

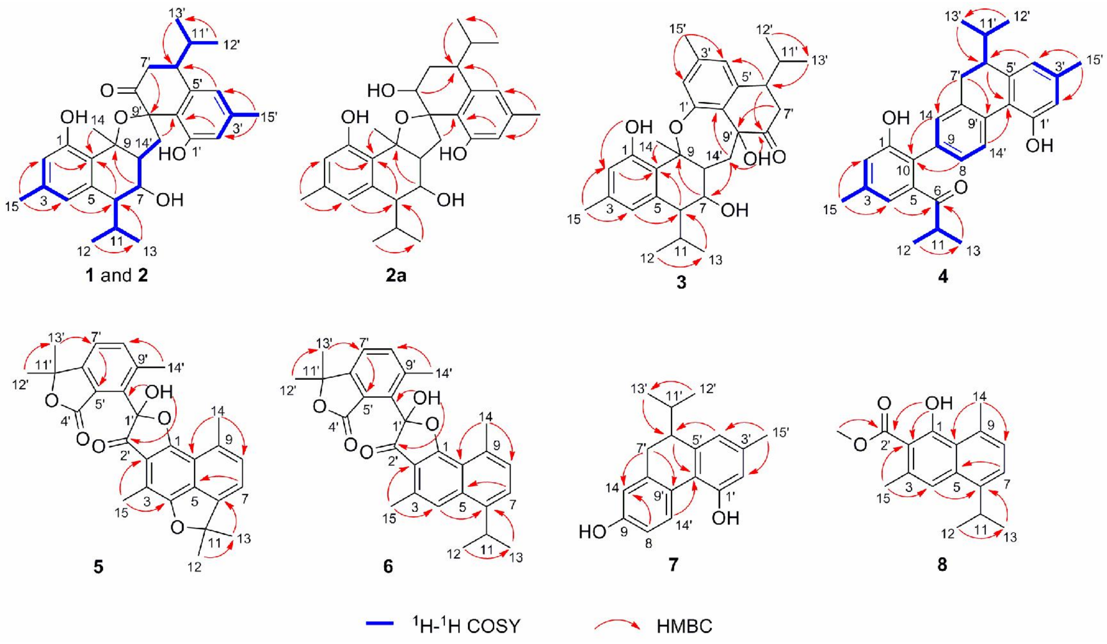

Figure S1. Key HMBC and ^{1}H-^{1}H COSY correlations of compounds **1**, **2**, **2a**, **3-8**.

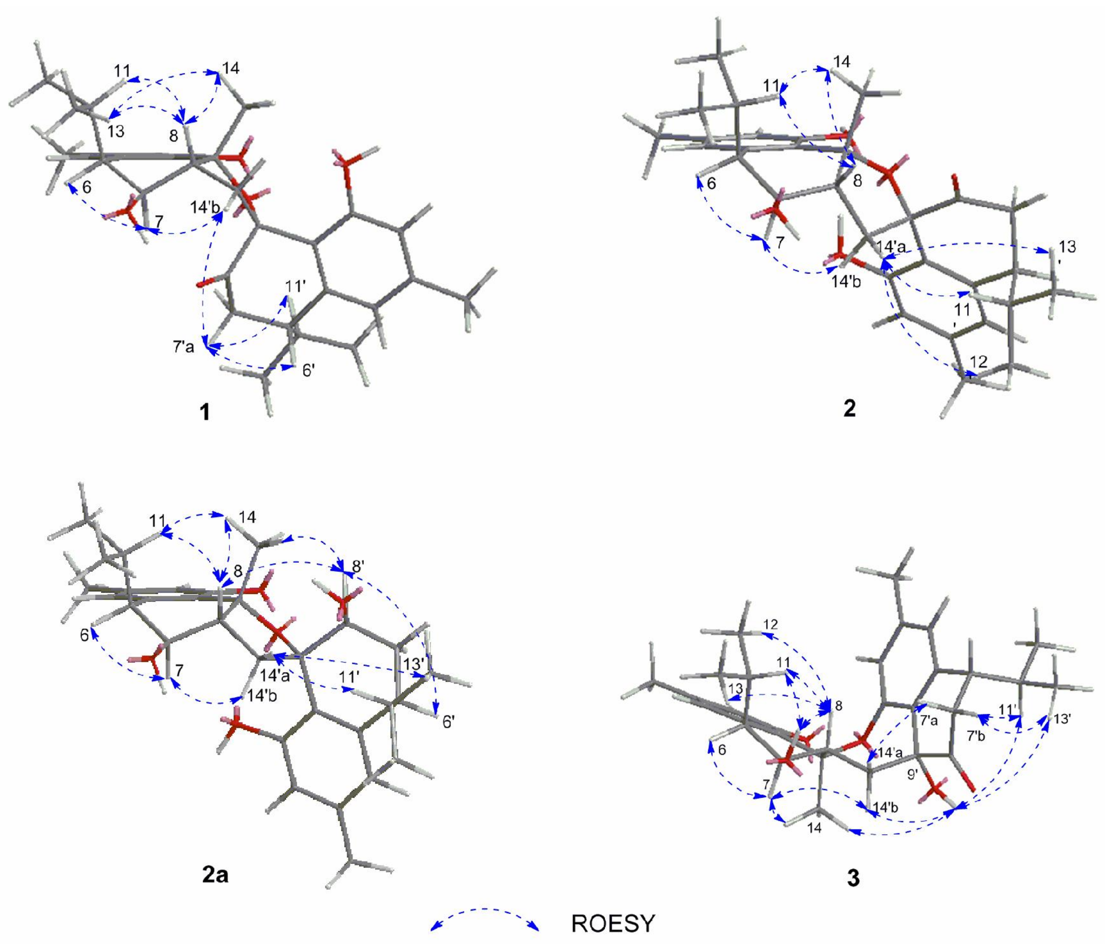

Figure S2. Key ROESY correlations of compounds **1**, **2**, **2a** and **3**.

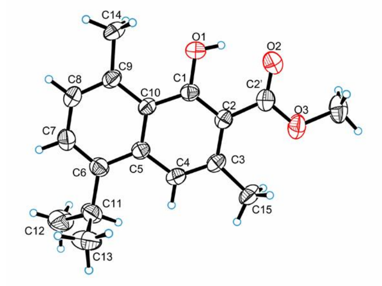

Figure S3. X-ray structure of compound **8**.

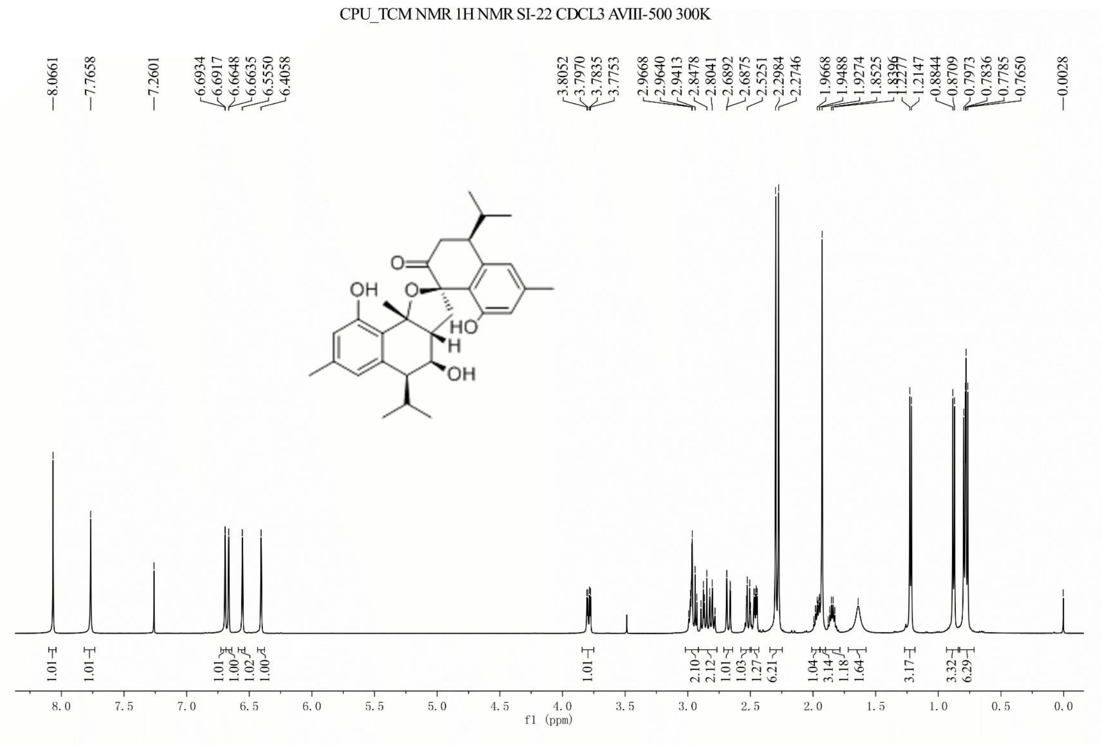

Figure S4. ^{1}H NMR spectrum of involucratusin A (**1**) in $CDCl_3$

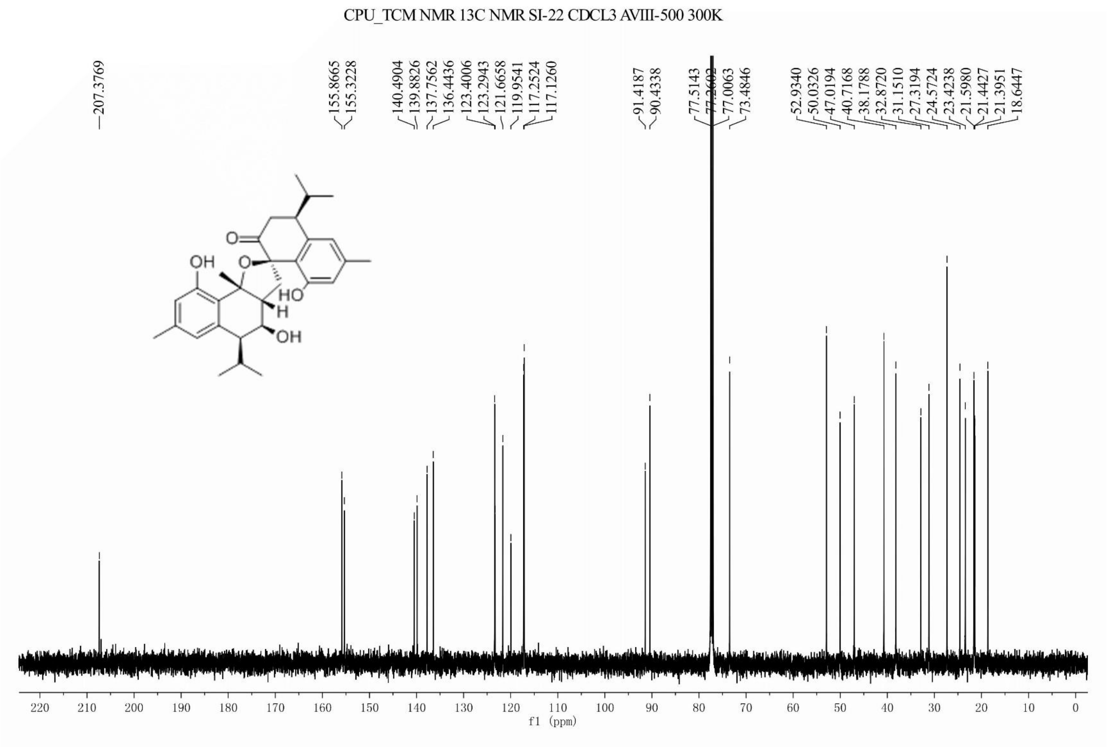

Figure S5. ^{13}C NMR spectrum of involucratusin A (**1**) in $CDCl_3$

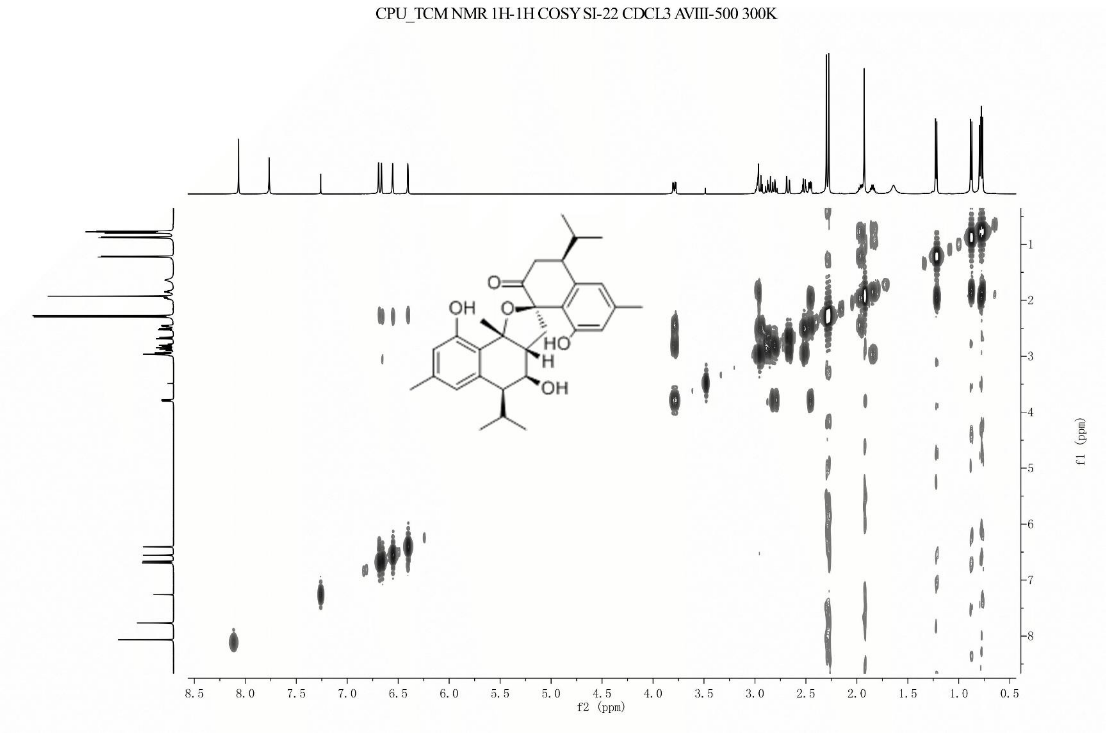

Figure S6. COSY spectrum of involucratusin A (**1**) in $CDCl_3$

CPU_TCM NMR HSQC SI-22 CDCL3 AVIII-500 300K

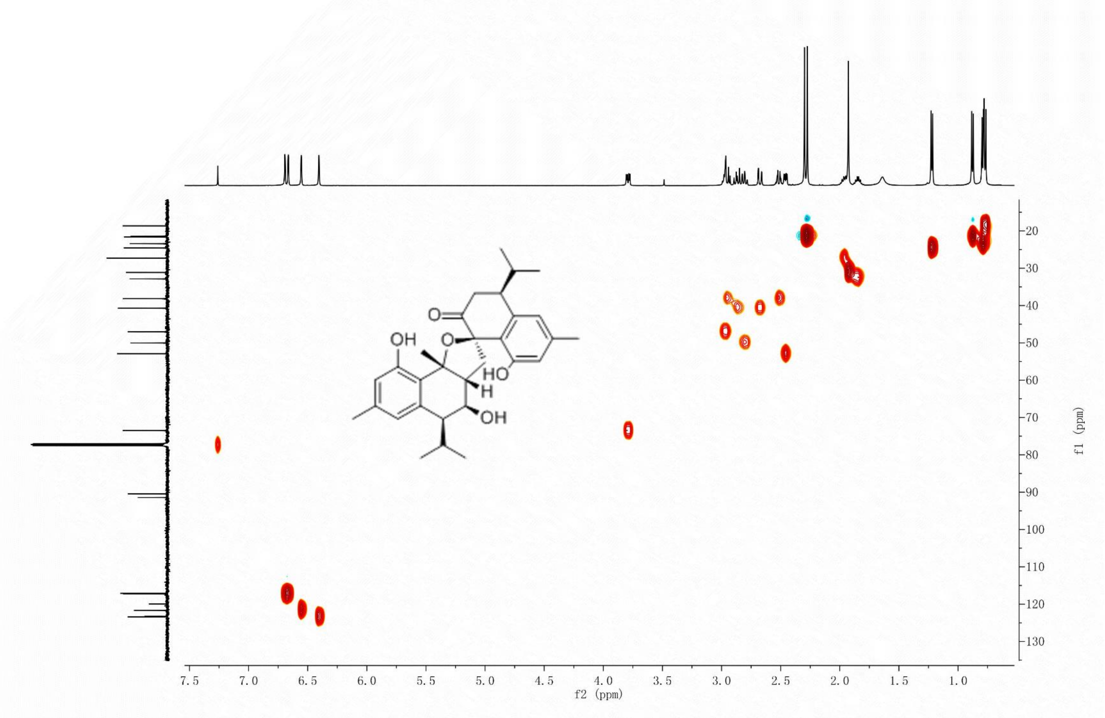

Figure S7. HSQC spectrum of involucratusin A (**1**) in $CDCl_3$

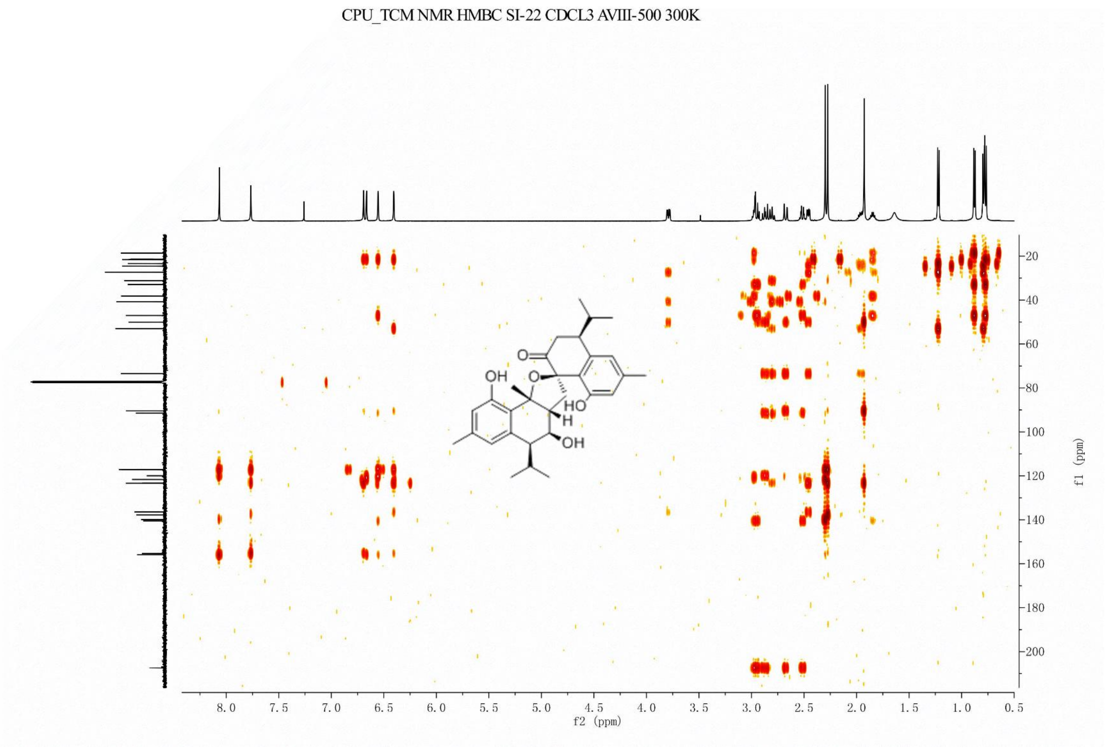

Figure S8. HMBC spectrum of involucratusin A (**1**) in $CDCl_3$

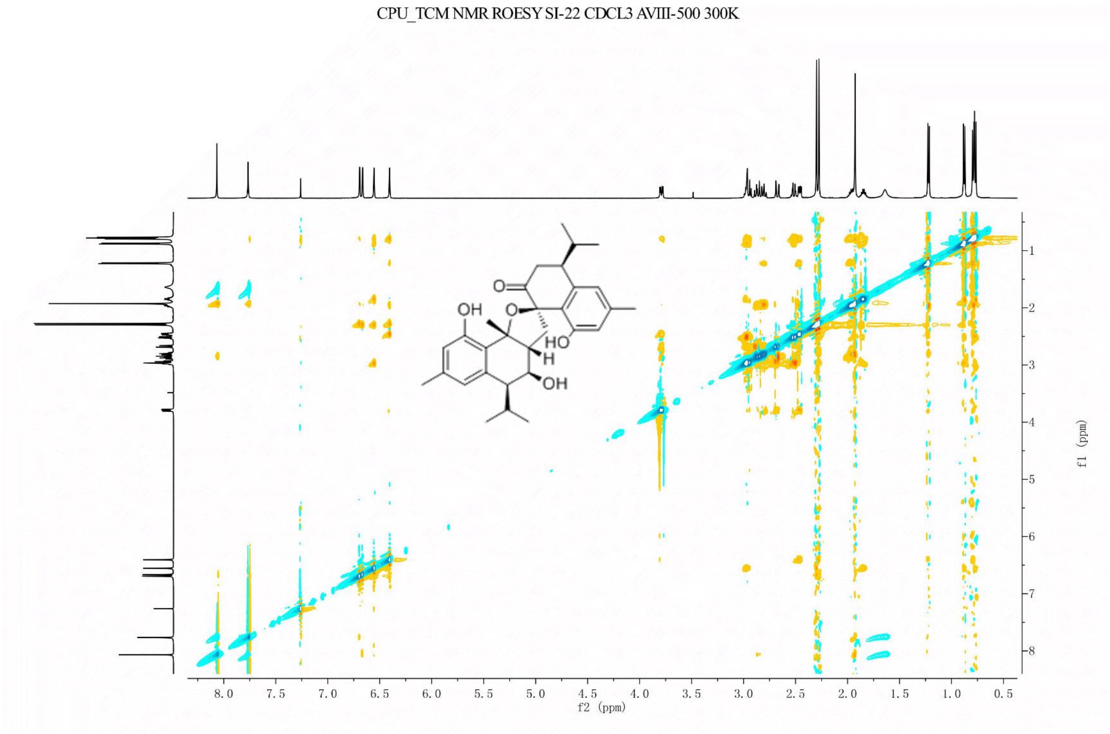

Figure S9. ROESY spectrum of involucratusin A (**1**) in $CDCl_3$

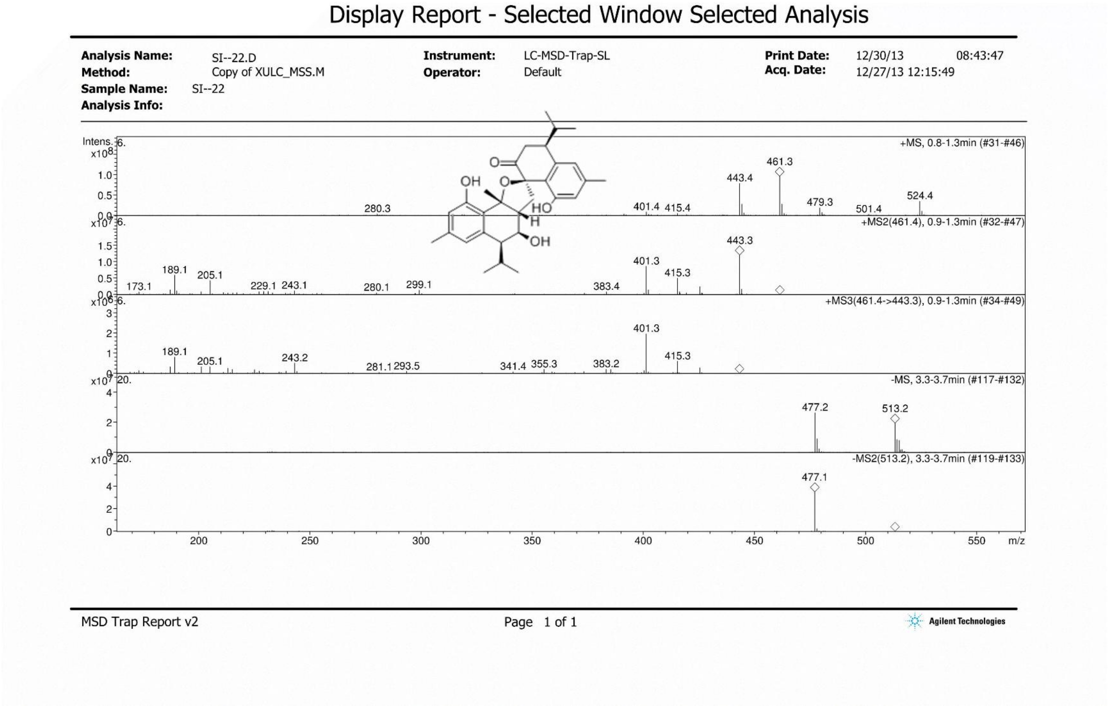

Figure S10. ESIMS spectrum of involucratusin A (**1**) in CH_3OH

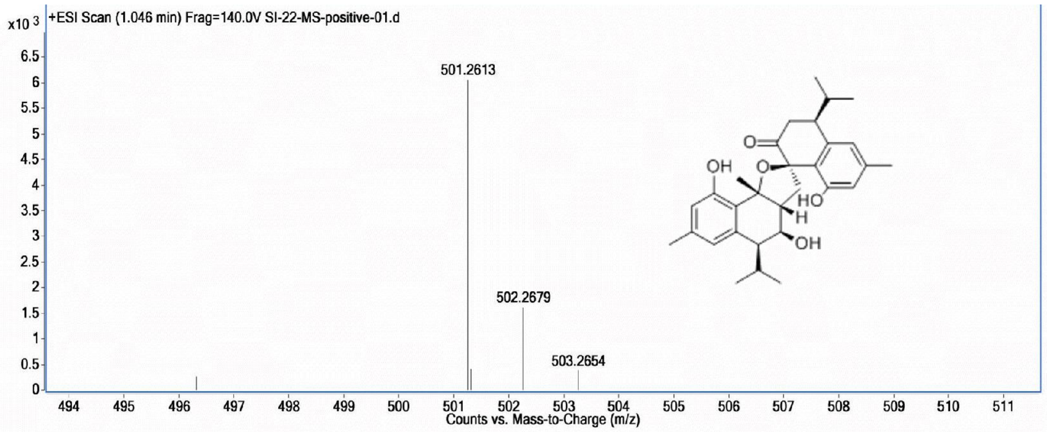

Elemental Composition Calculator

Target m/z:	501.2613	Result type:	Positive ions	Species:	$[M+Na]^+$
Elements:		C (0-80); H (0-120); O (0-30); N(0-10); Na (0-5); S (0-5)			
Ion Formula		Calculated m/z		PPM Error	
C30H38NaO5		501.2611		-0.28	

Agilent Technologies

Figure S11. HRESIMS spectrum of involucratusin A (**1**) in CH_3OH

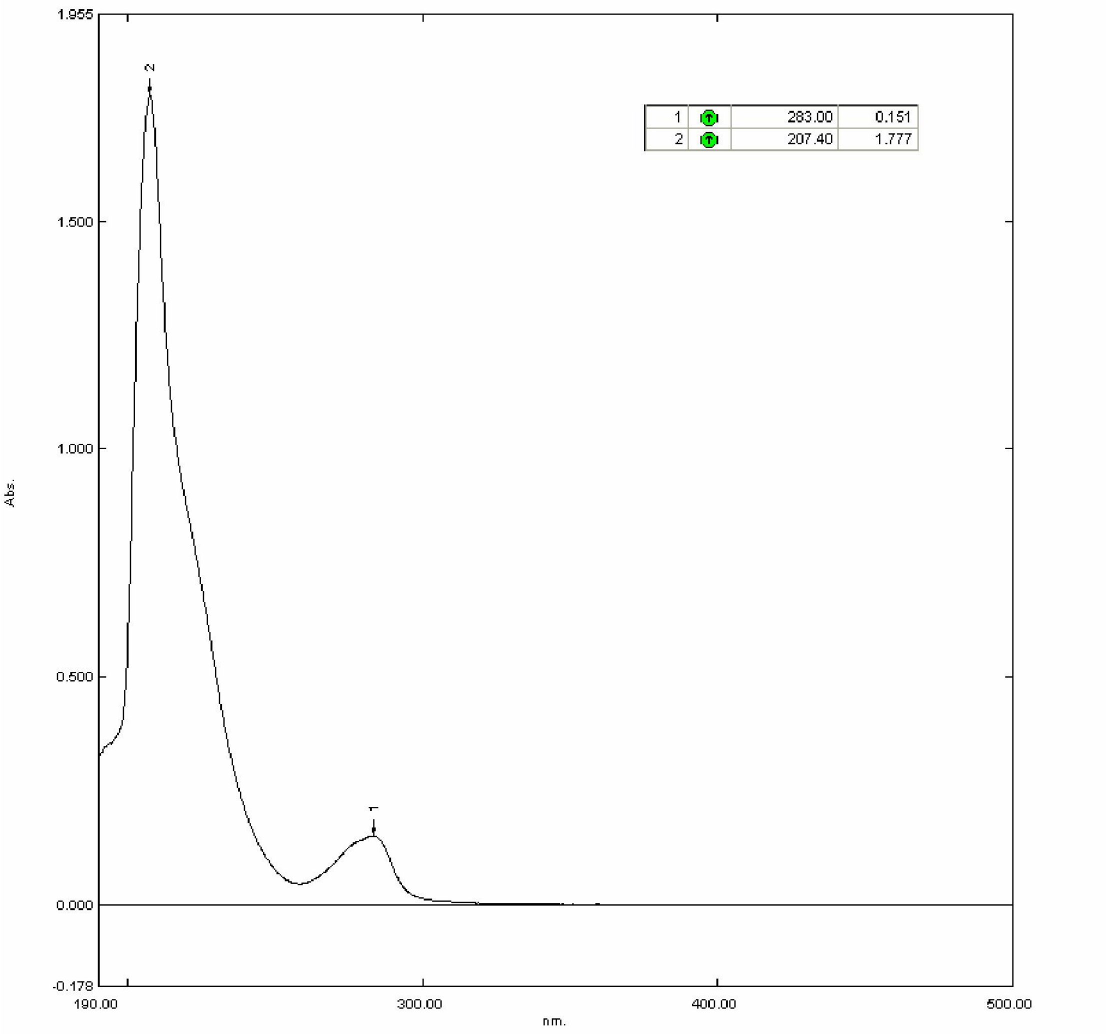

Figure S12. UV spectrum of involucratusin A (**1**) in CH_3OH

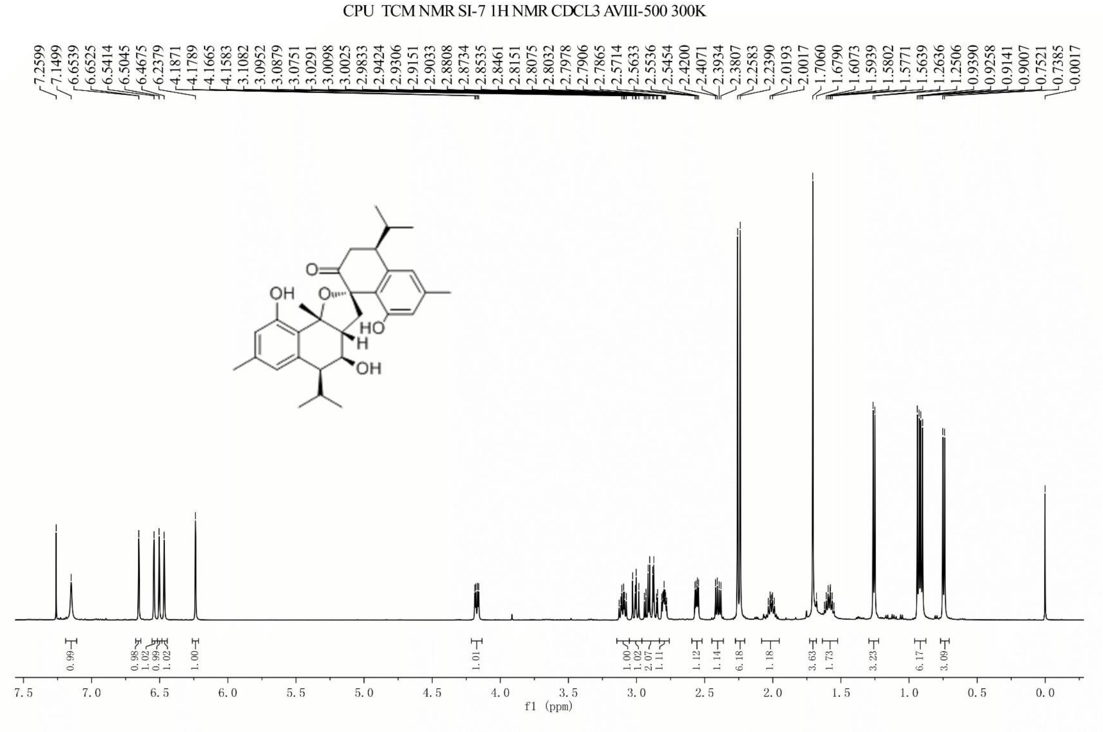

Figure S13. ^{1}H NMR spectrum of involucratusin B (**2**) in $CDCl_3$

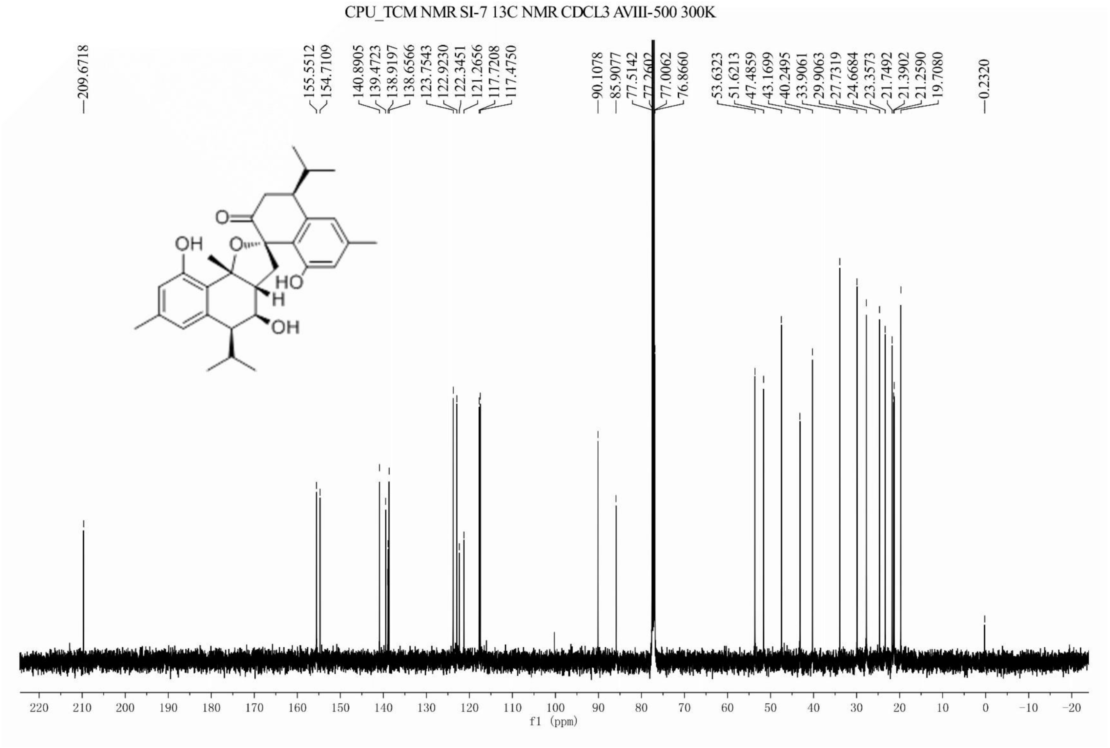

Figure S14. ^{13}C NMR spectrum of involucratusin B (**2**) in $CDCl_3$

CPU_TCM NMR SI-7 HSQC CDCL3 AVIII-500 300K

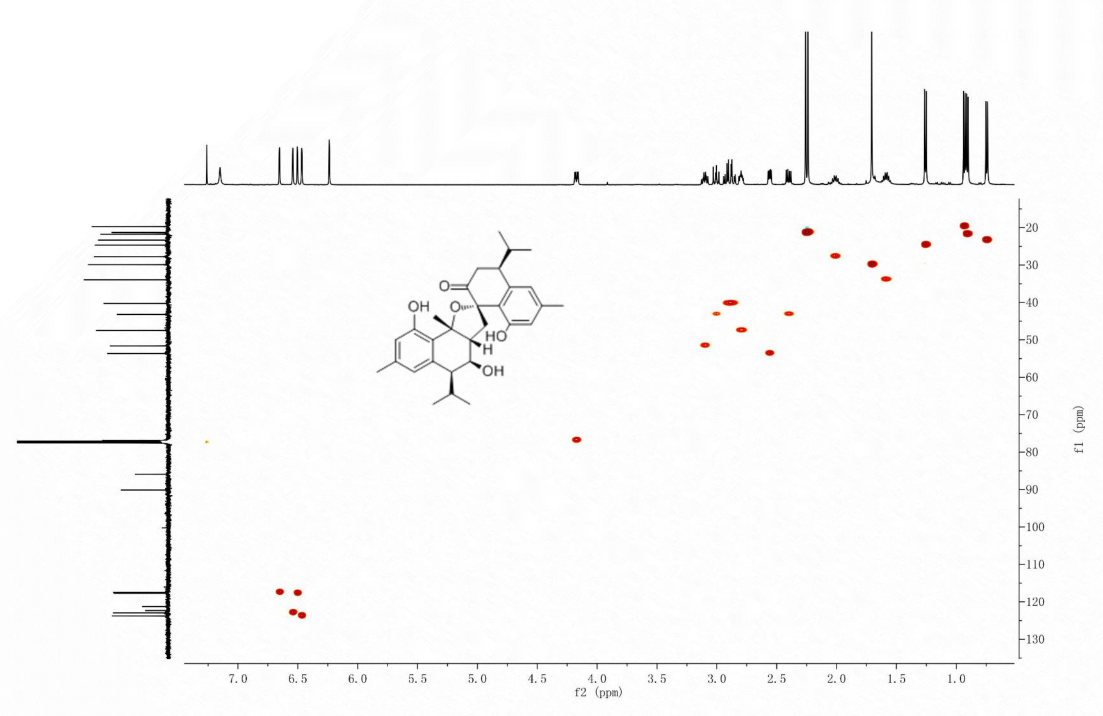

Figure S15. HSQC spectrum of involucratusin B (**2**) in $CDCl_3$

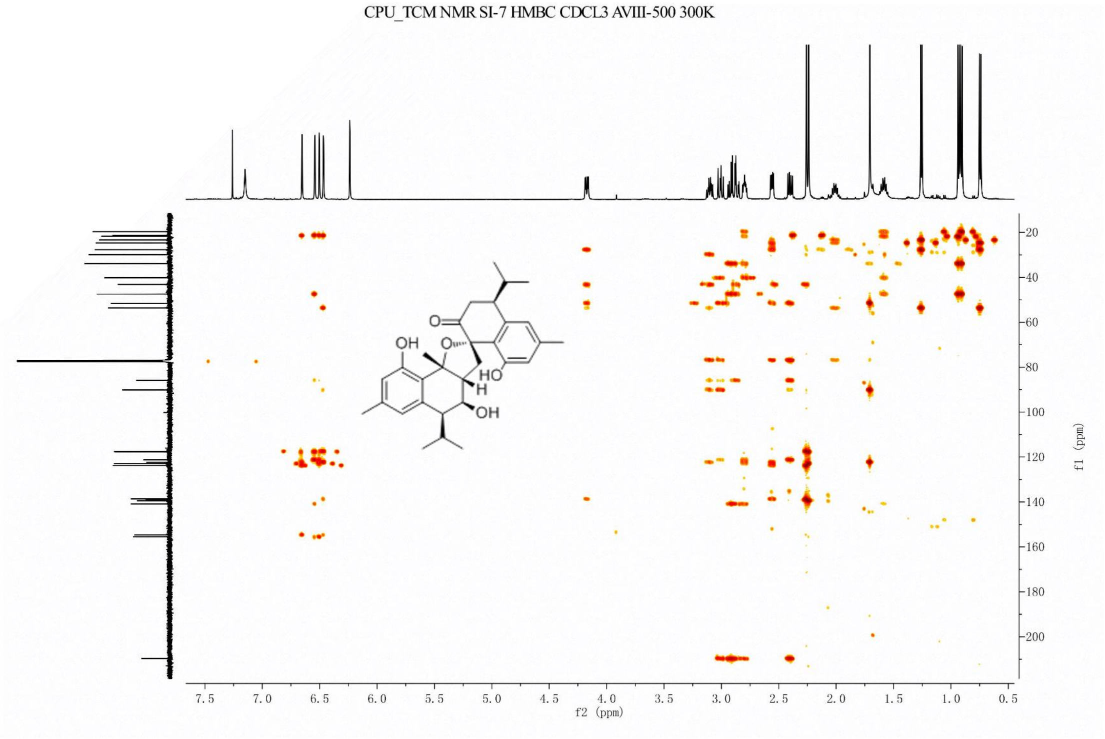

Figure S16. HMBC spectrum of involucratusin B (**2**) in $CDCl_3$

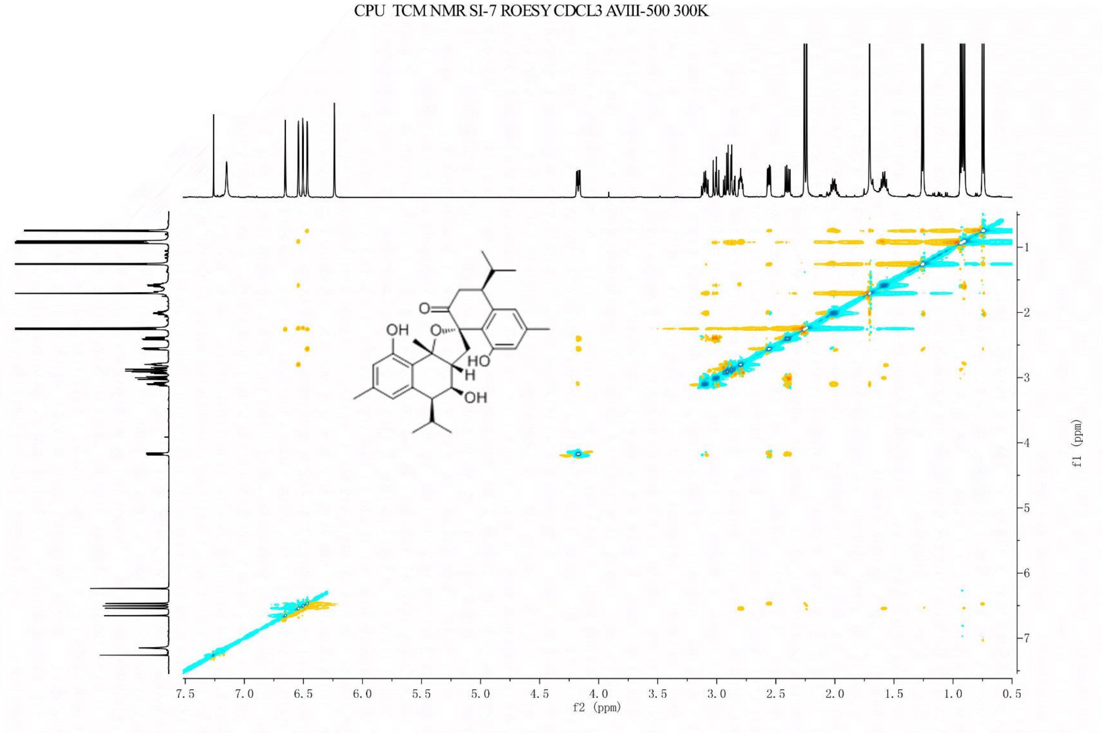

Figure S17. ROESY spectrum of involucratusin B (**2**) in $CDCl_3$

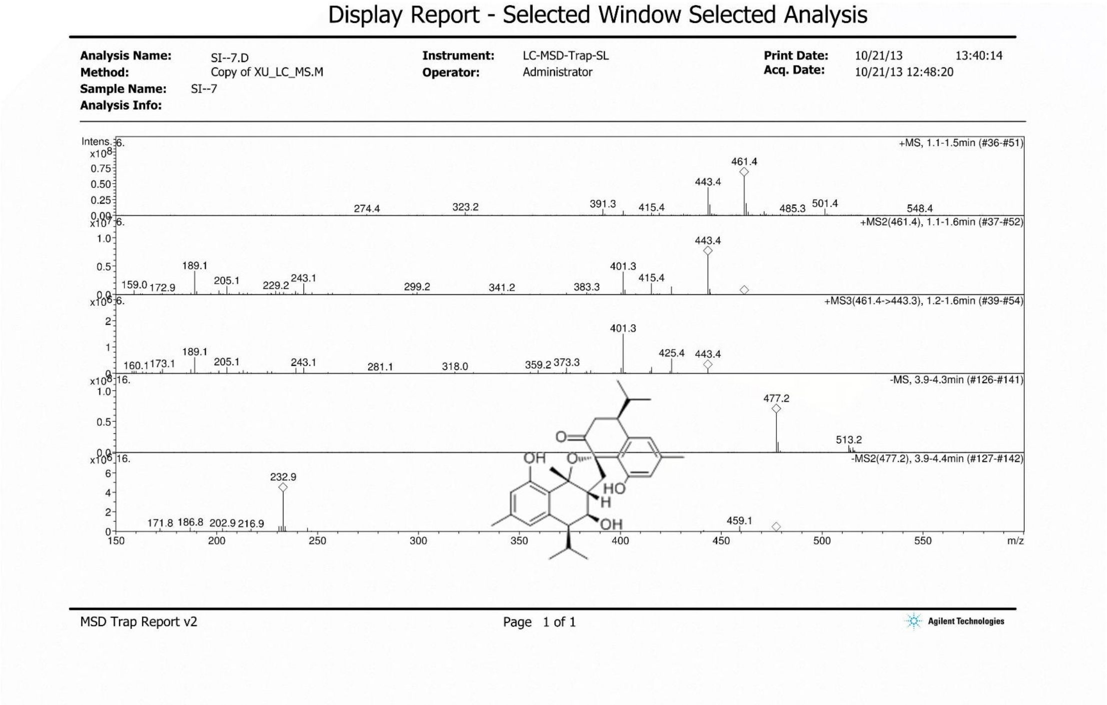

Figure S18. ESIMS spectrum of involucratusin B (**2**) in CH_3OH

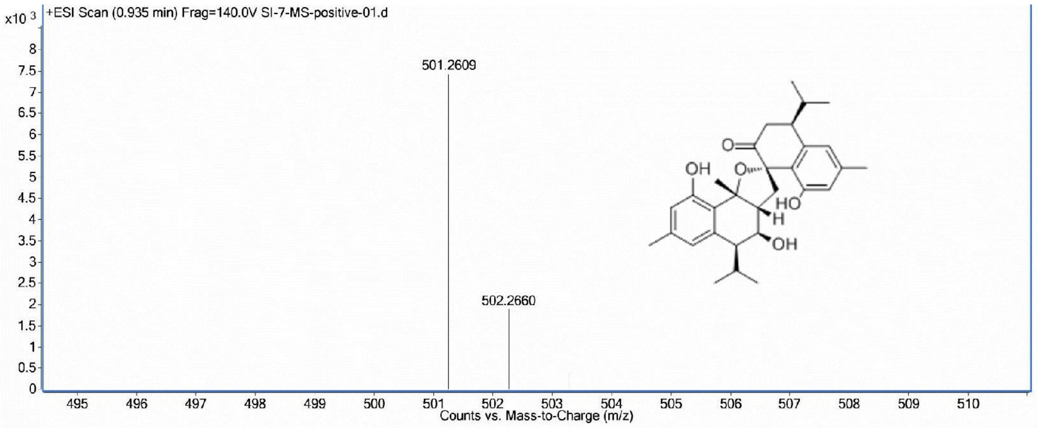

Elemental Composition Calculator

**Target m/z:**	501.2609	**Result type:**	Positive ions	**Species:**	[M+Na]$^{-}$
**Elements:**		C (0-80); H (0-120); O (0-30); N(0-10); Na (0-5); S (0-5)			
**Ion Formula**		**Calculated m/z**		**PPM Error**	
C30H38NaO5		501.2611		0.41	

Agilent Technologies

Figure S19. HRESIMS spectrum of involucratusin B (**2**) in CH_3OH

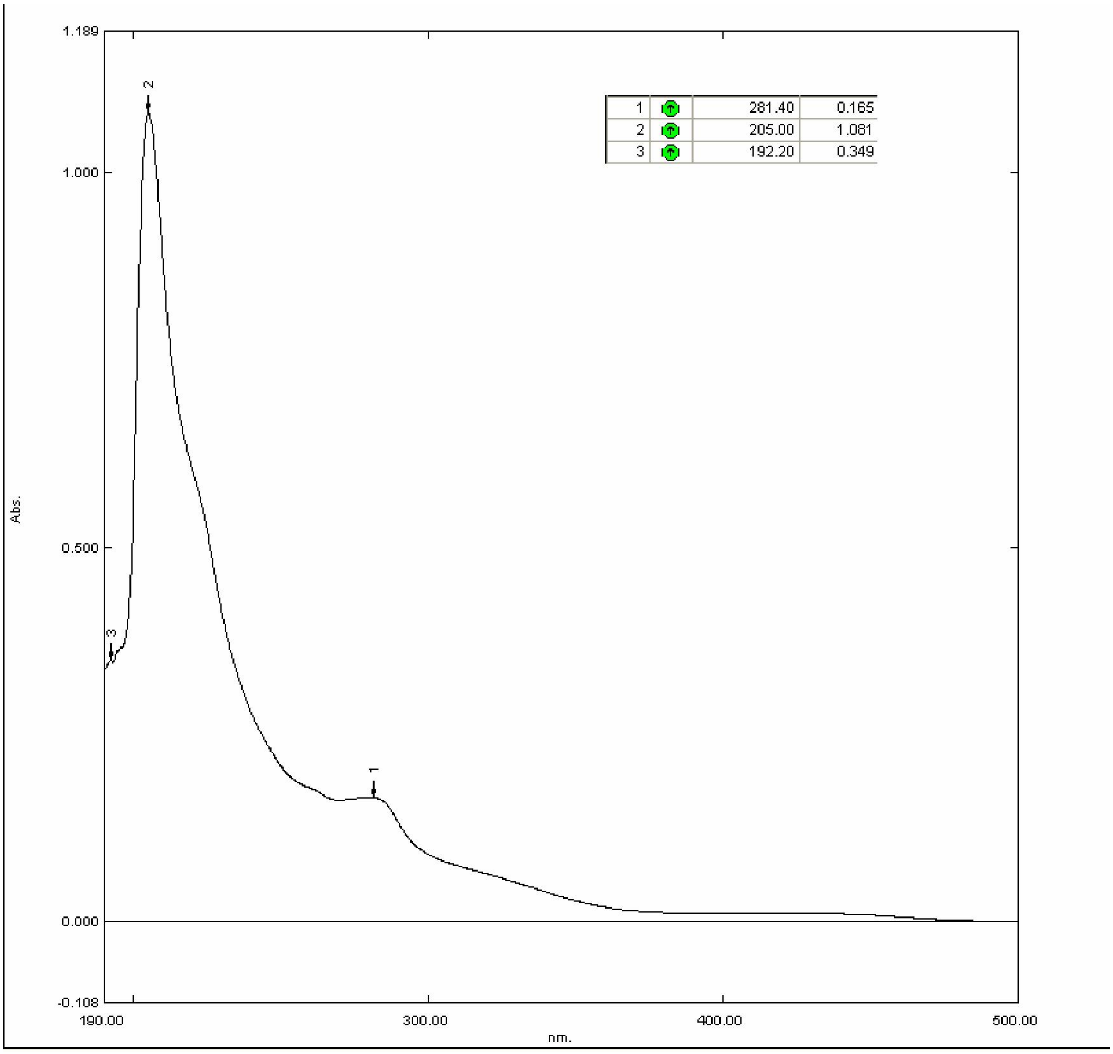

Figure S20. UV spectrum of involucratusin B (**2**) in CH_3OH

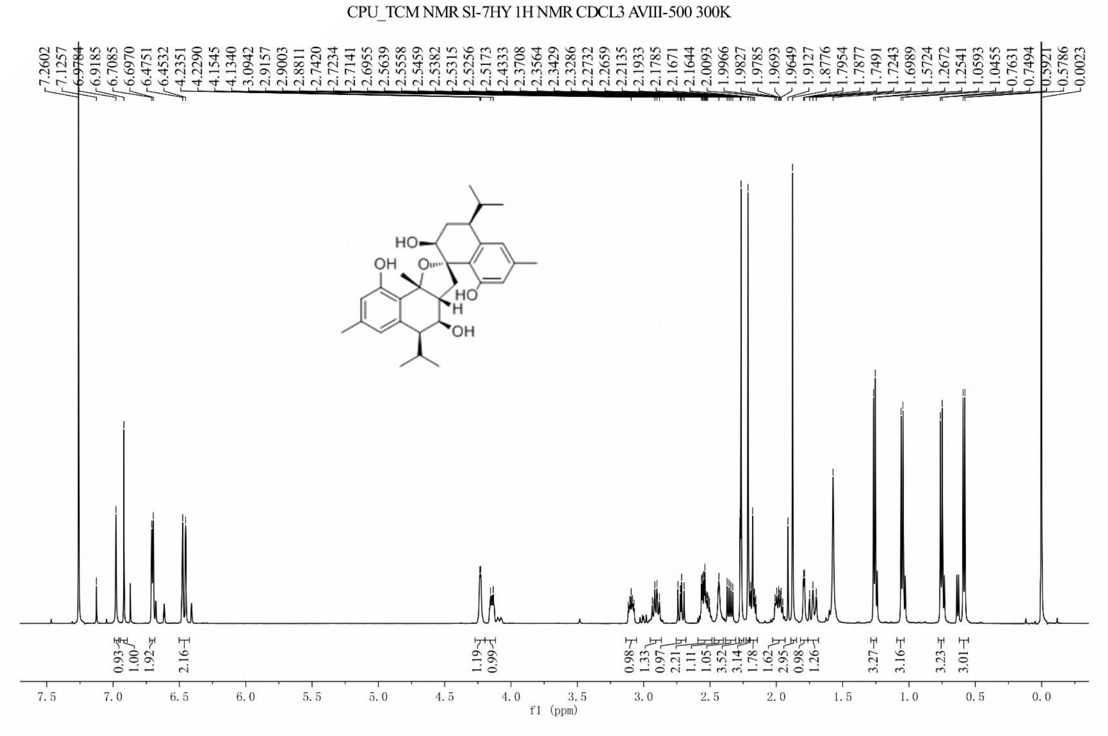

Figure S21. ^{1}H NMR spectrum of compound **2a** in $CDCl_3$

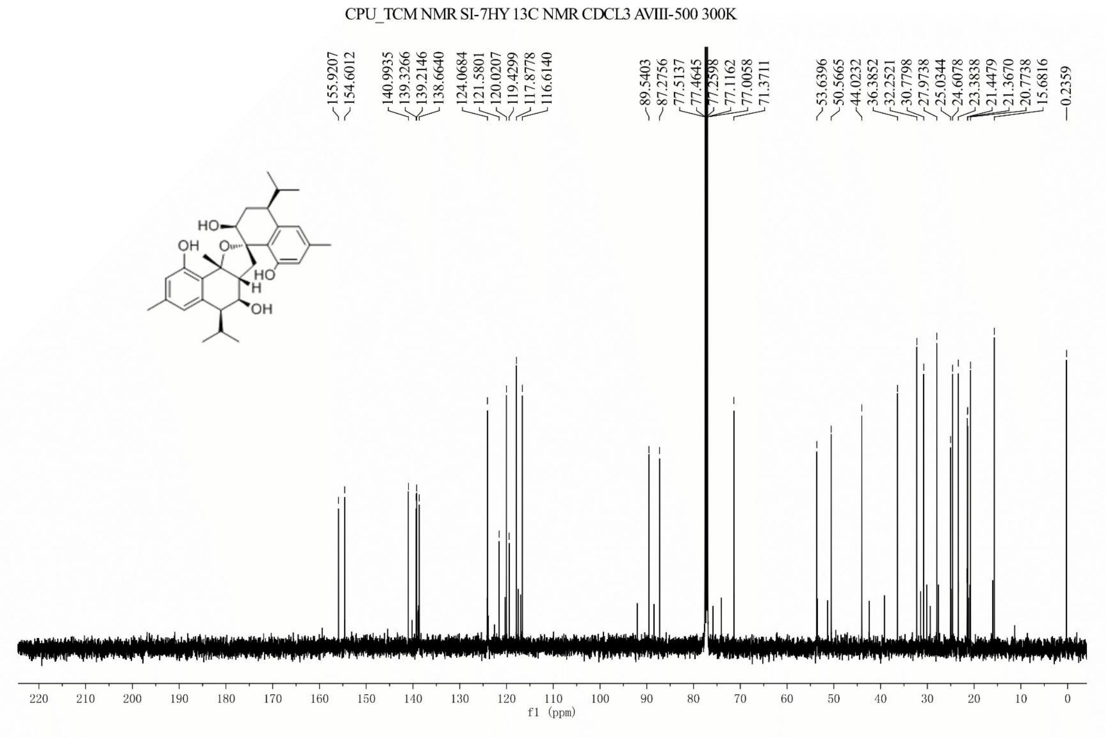

Figure S22. ^{13}C NMR spectrum of compound **2a** in $CDCl_3$

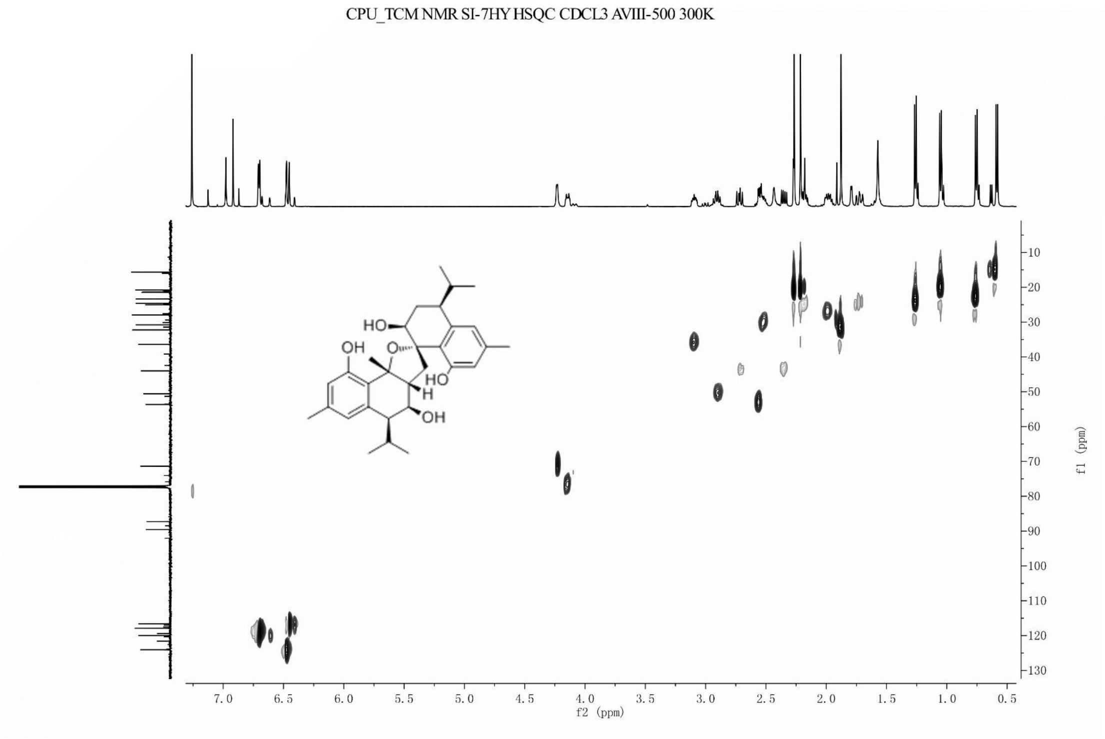

Figure S23. HSQC spectrum of compound **2a** in $CDCl_3$

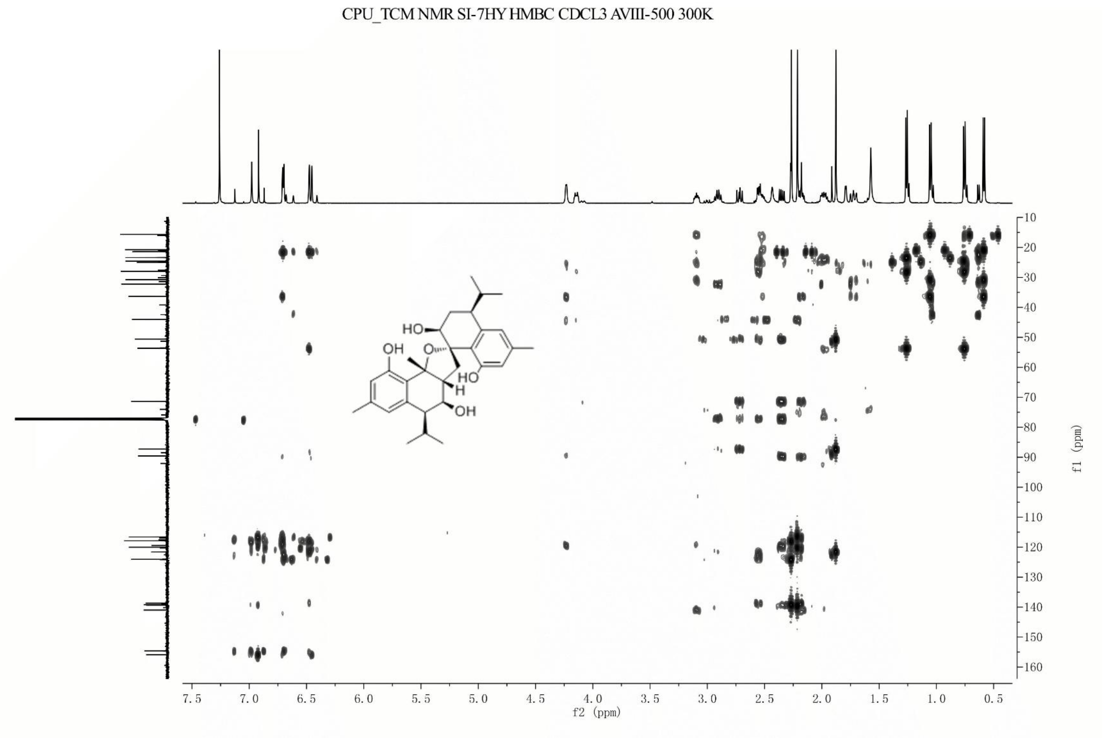

Figure S24. HMBC spectrum of compound **2a** in $CDCl_3$

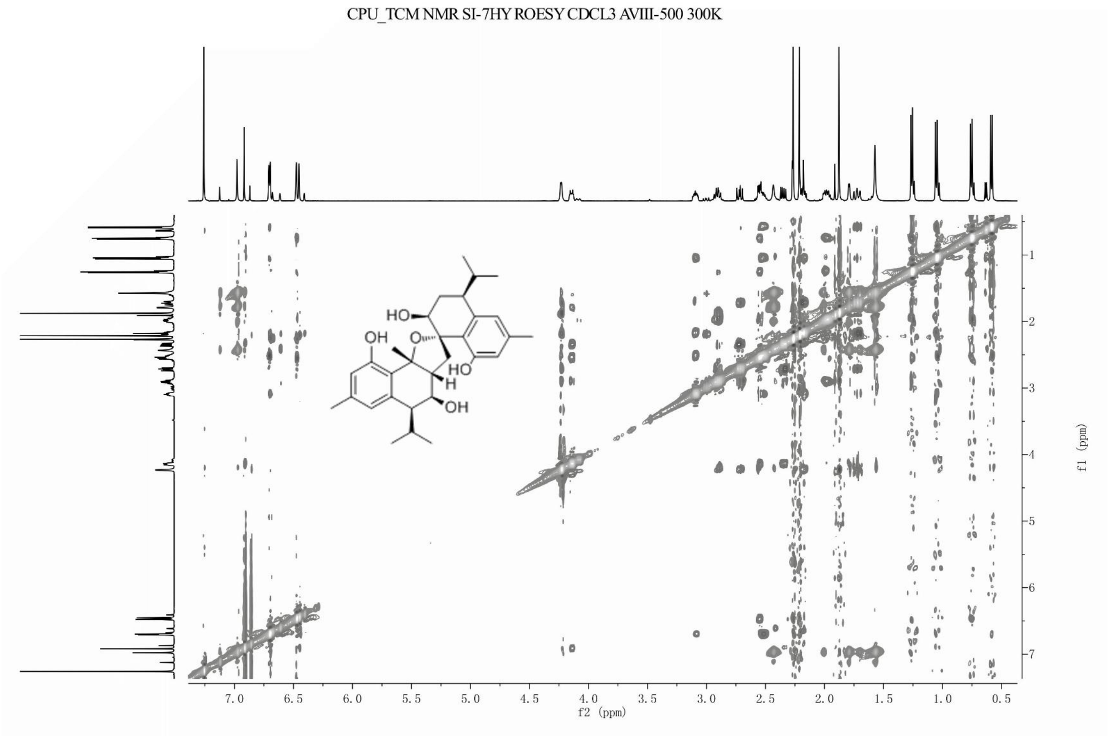

Figure S25. ROESY spectrum of compound **2a** in $CDCl_3$

Display Report - Selected Window Selected Analysis

Analysis Name: SI--7HY.d
Method: XU_MS.M
Sample Name: Sample
Analysis Info:

Instrument: amaZon SL
Operator: bruker

Print Date: 2014-07-02 10:59:21 AM
Acq. Date: 2014-07-02 10:53:38 AM

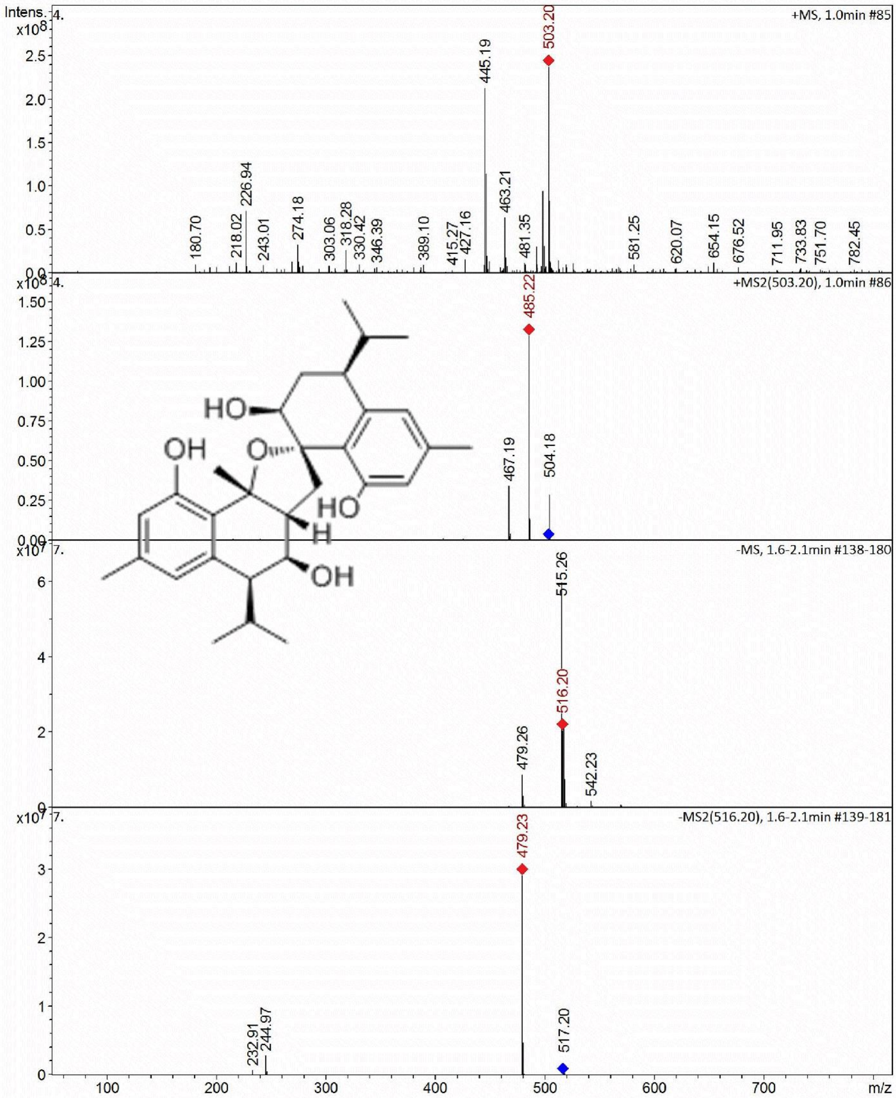

MSD Trap Report v2 Page 1 of 1

Figure S26. ESIMS spectrum of compound **2a** in CH_3OH

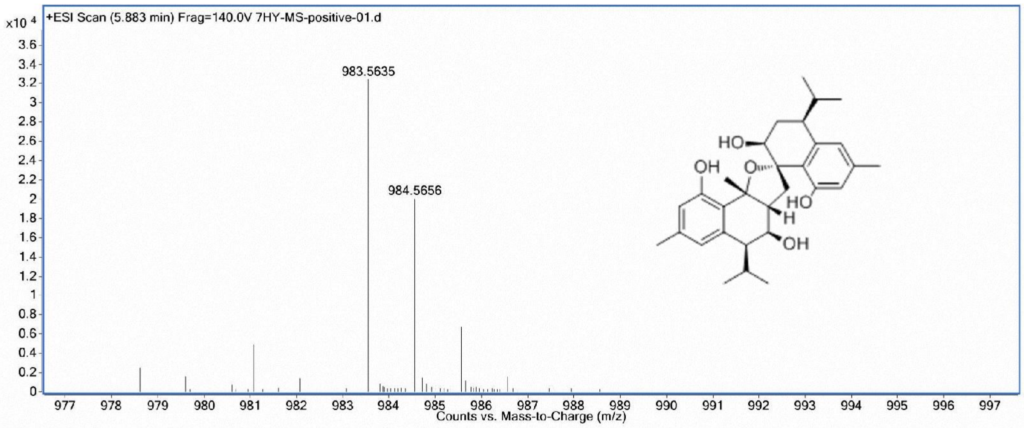

Elemental Composition Calculator

**Target m/z:**	983.5635	**Result type:**	Positive ions	**Species:**	$[2M+Na]^+$
**Elements:**		C (0-80); H (0-120); O (0-30); N(0-10); Na (0-5)			
**Ion Formula**		**Calcalated m/z**		**PPM Error**	
C60H80NaO10		983.5644		0.88	

Agilent Technologies

Figure S27. HRESIMS spectrum of compound **2a** in CH_3OH

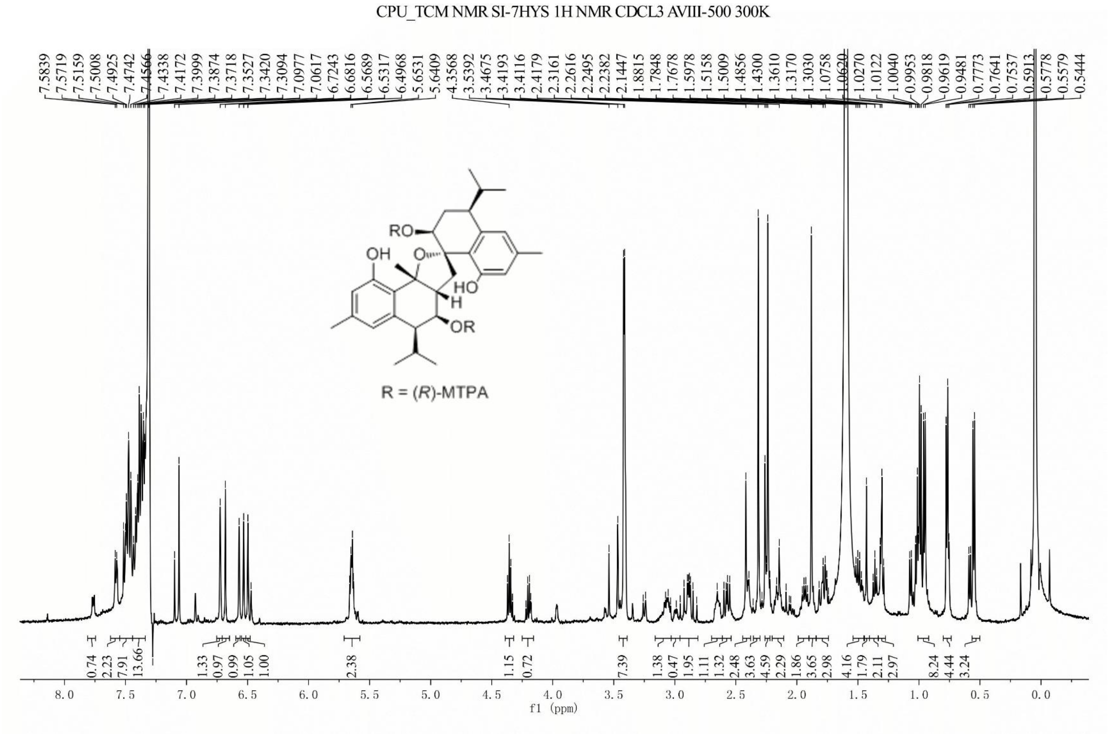

Figure S28. ^{1}H NMR spectrum of compound **2aa** in $CDCl_3$

Display Report - Selected Window Selected Analysis

Analysis Name: SI--7HYS.d **Instrument:** amaZon SL **Print Date:** 2014-07-23 9:56:42 AM
Method: XU_MS.M **Operator:** bruker **Acq. Date:** 2014-07-23 9:52:42 AM
Sample Name: Sample
Analysis Info:

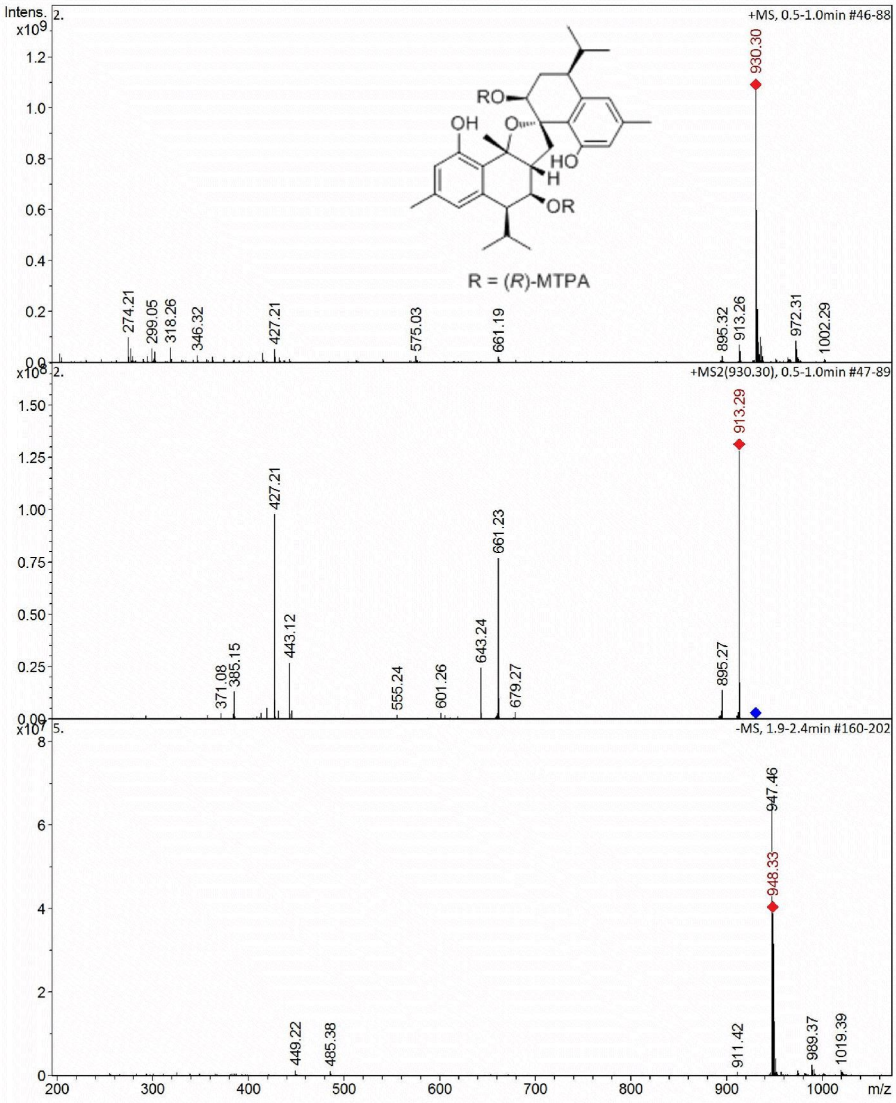

MSD Trap Report v2 Page 1 of 1

Figure S29. ESIMS spectrum of compound **2aa** in CH_3OH

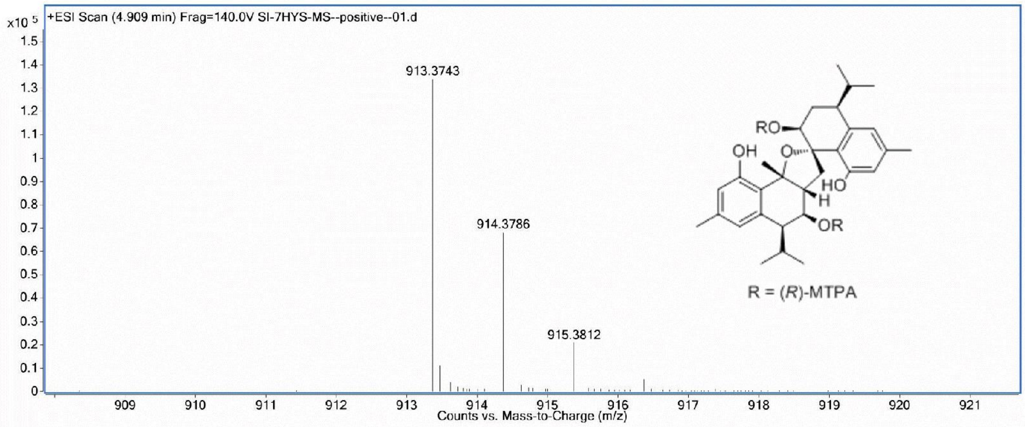

Elemental Composition Calculator

Target m/z:	913.3743	Result type:	Positive ions	Species:	[M+H]$^+$
**Elements:**		C (0-80); H (0-120); O (0-30); F(0-10); Na (0-5)			
**Ion Formula**		**Calcalated m/z**		**PPM Error**	
C50H55F6O9		913.3745		0.16	

Agilent Technologies

Figure S30. ESIMS spectrum of compound **2aa** in CH_3OH

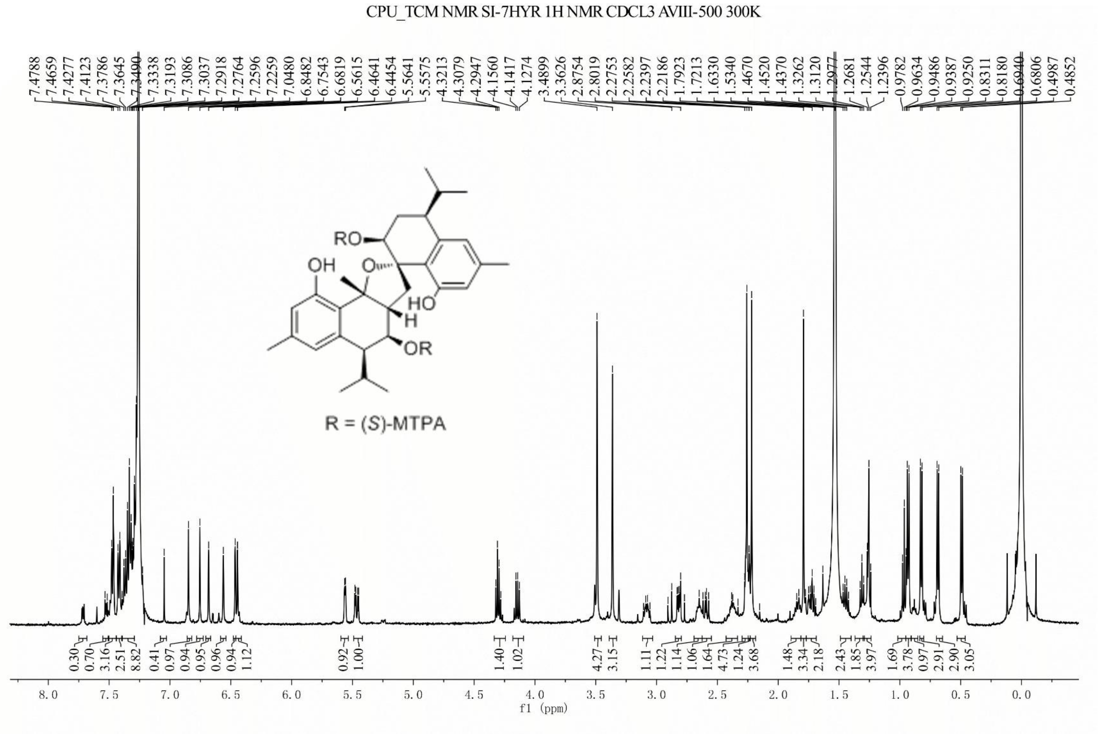

Figure S31. ^{1}H NMR spectrum of compound **2ab** in $CDCl_3$

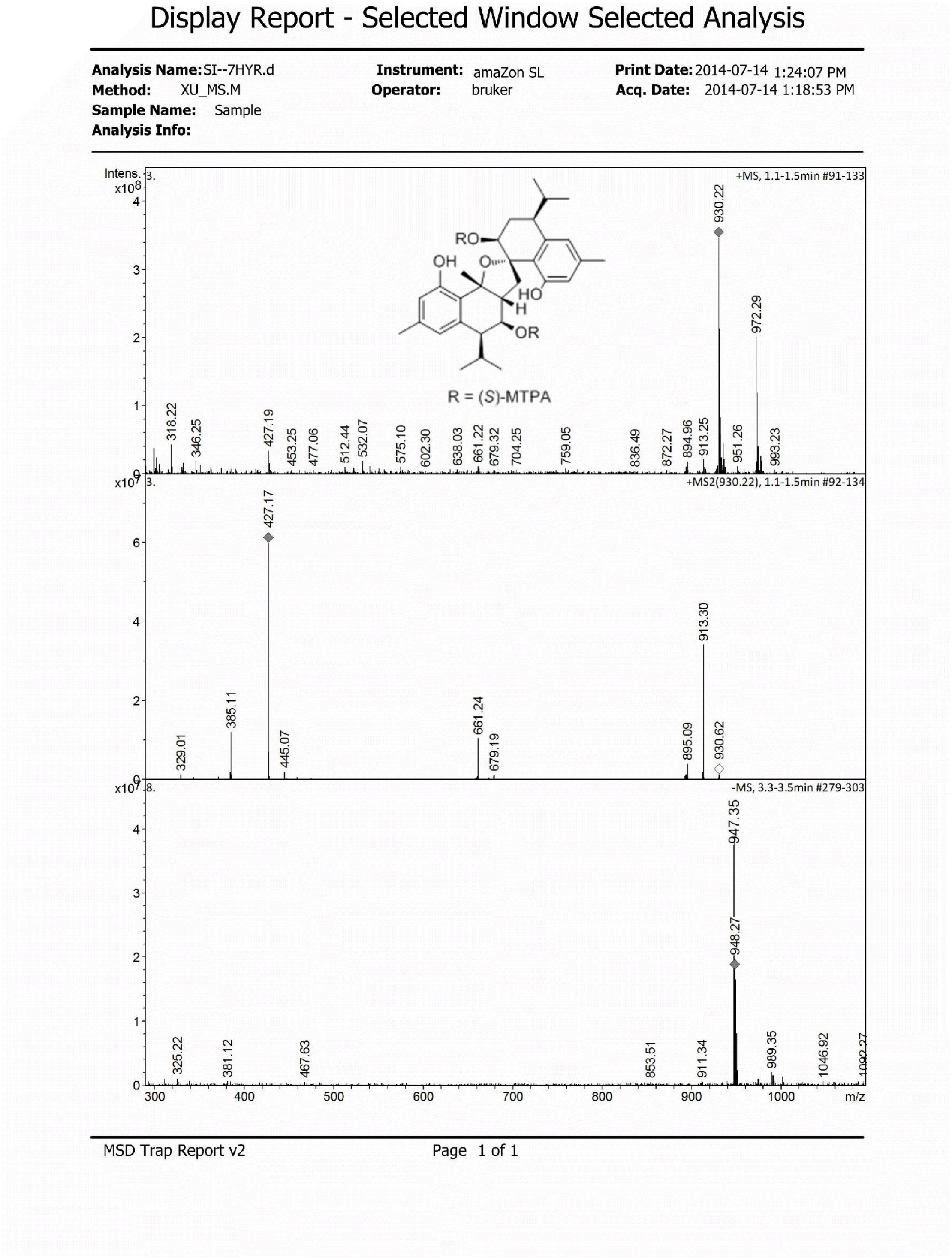

Figure S32. ESIMS spectrum of compound **2ab** in CH_3OH

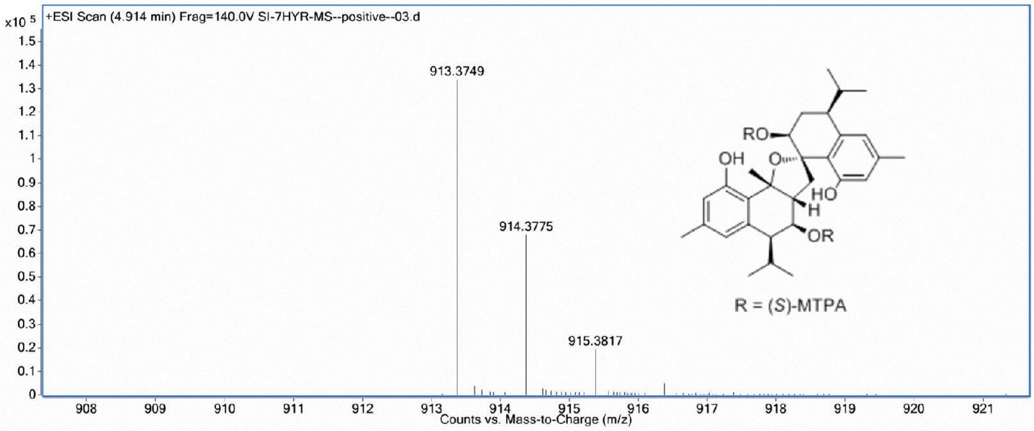

Elemental Composition Calculator

**Target m/z:**	913.3749	**Result type:**	Positive ions	**Species:**	$[M+H]^+$
**Elements:**		C (0-80); H (0-120); O (0-30); F(0-10); Na (0-5)			
**Ion Formula**		**Calcalated m/z**		**PPM Error**	
C50H55F6O9		913.3745		-0.47	

Agilent Technologies

Figure S33. ESIMS spectrum of compound **2ab** in CH_3OH

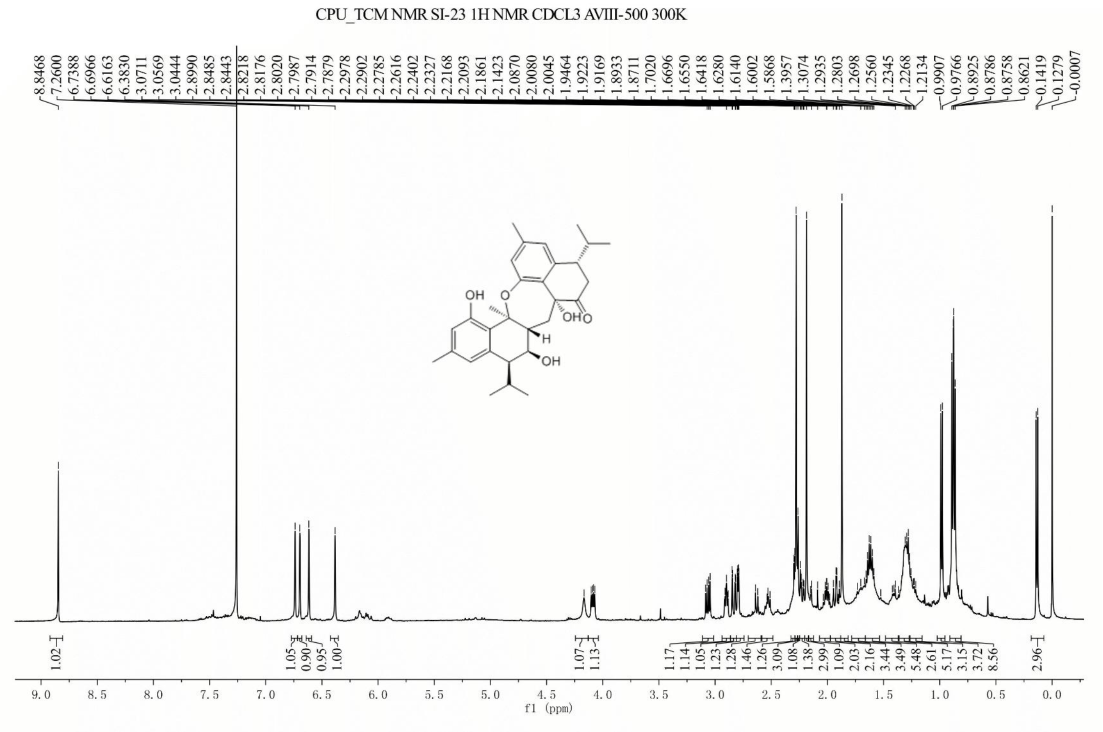

Figure S34. ^{1}H NMR spectrum of involucratusin C (**3**) in $CDCl_3$

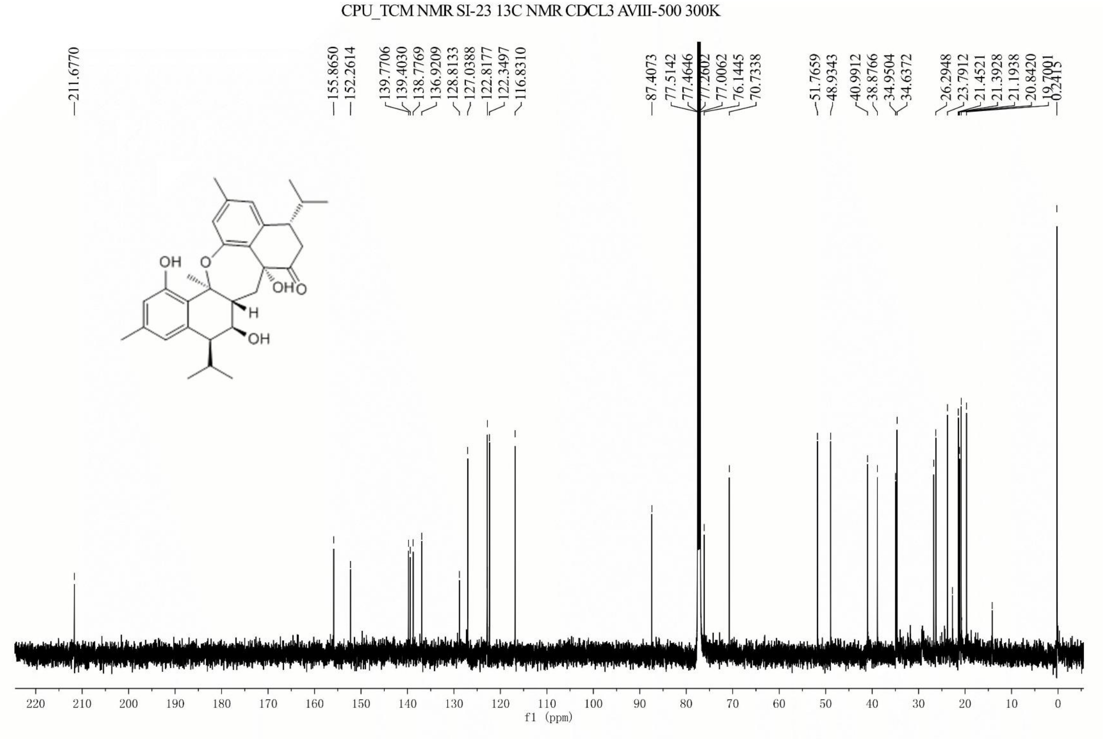

Figure S35. ^{13}C NMR spectrum of involucratusin C (**3**) in $CDCl_3$

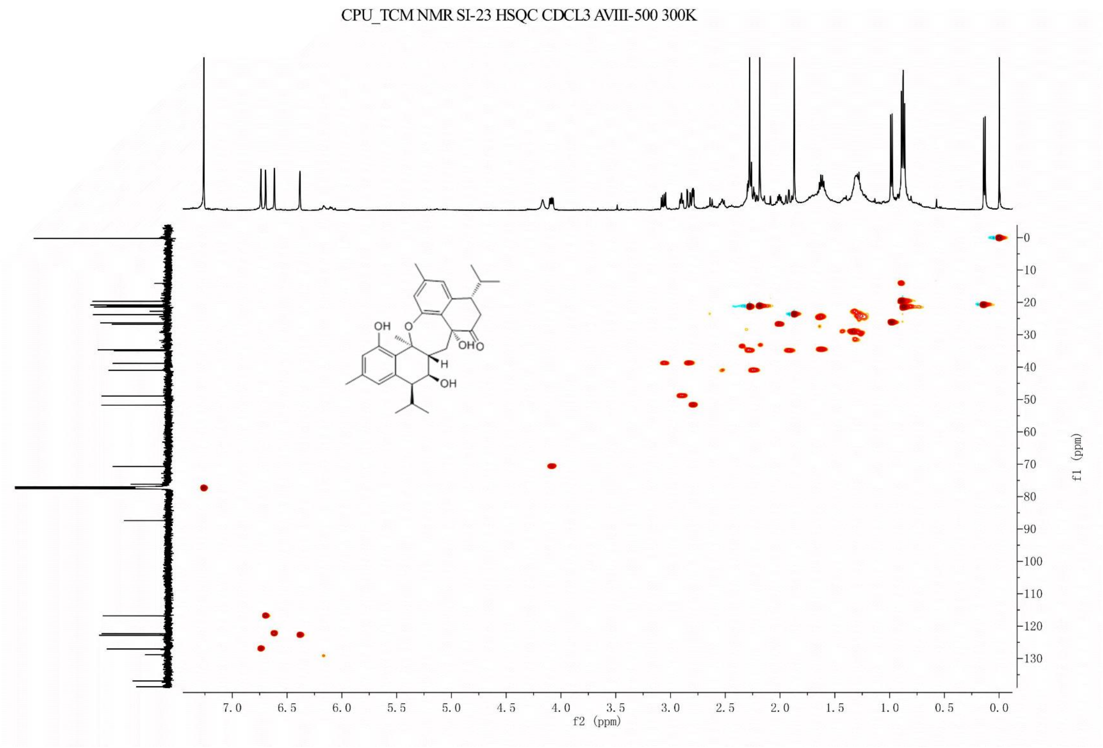

Figure S36. HSQC spectrum of involucratusin C (**3**) in $CDCl_3$

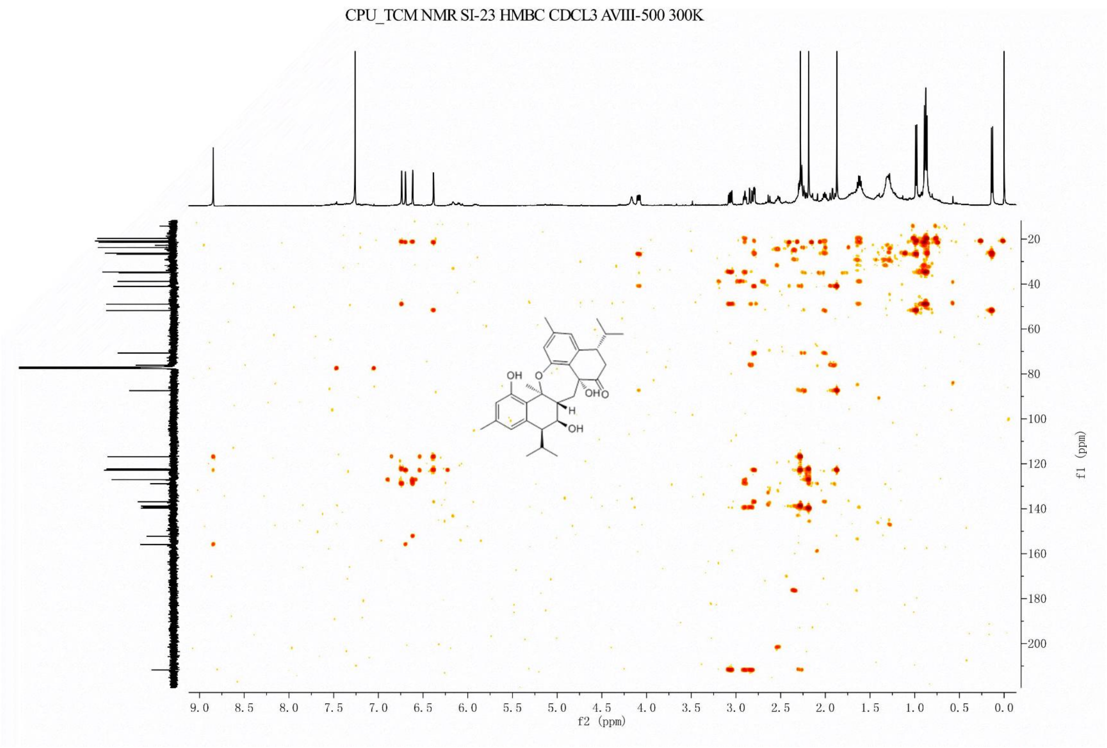

Figure S37. HMBC spectrum of involucratusin C (**3**) in $CDCl_3$

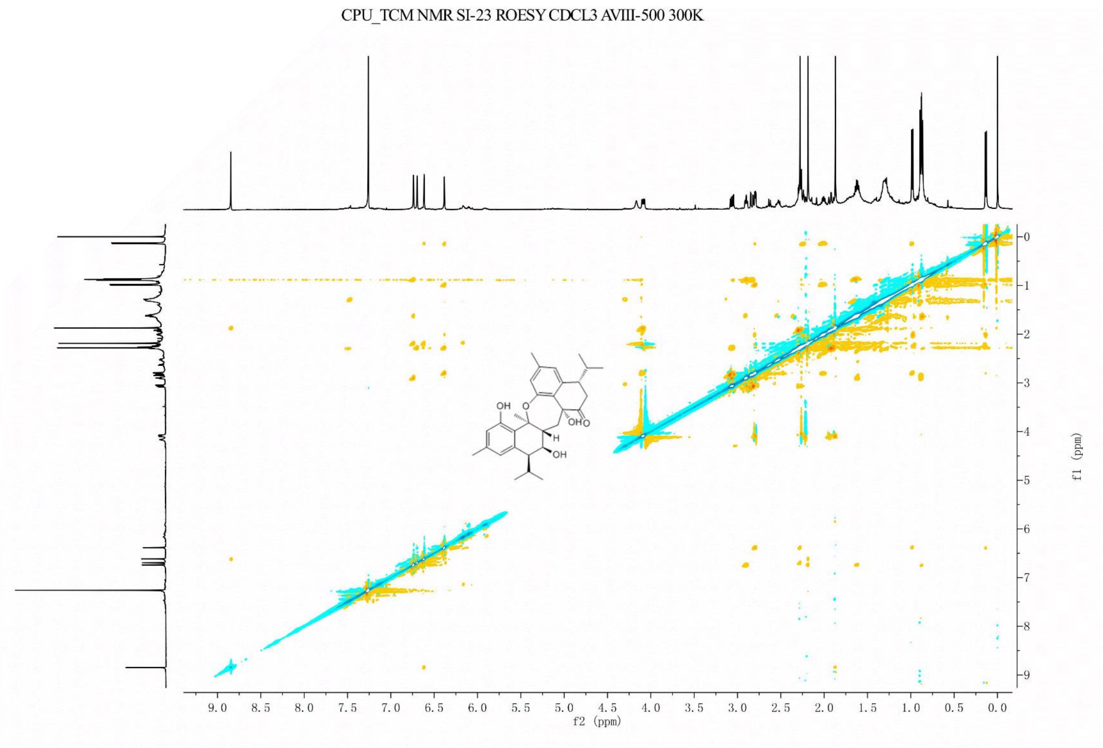

Figure S38. ROESY spectrum of involucratusin C (**3**) in $CDCl_3$

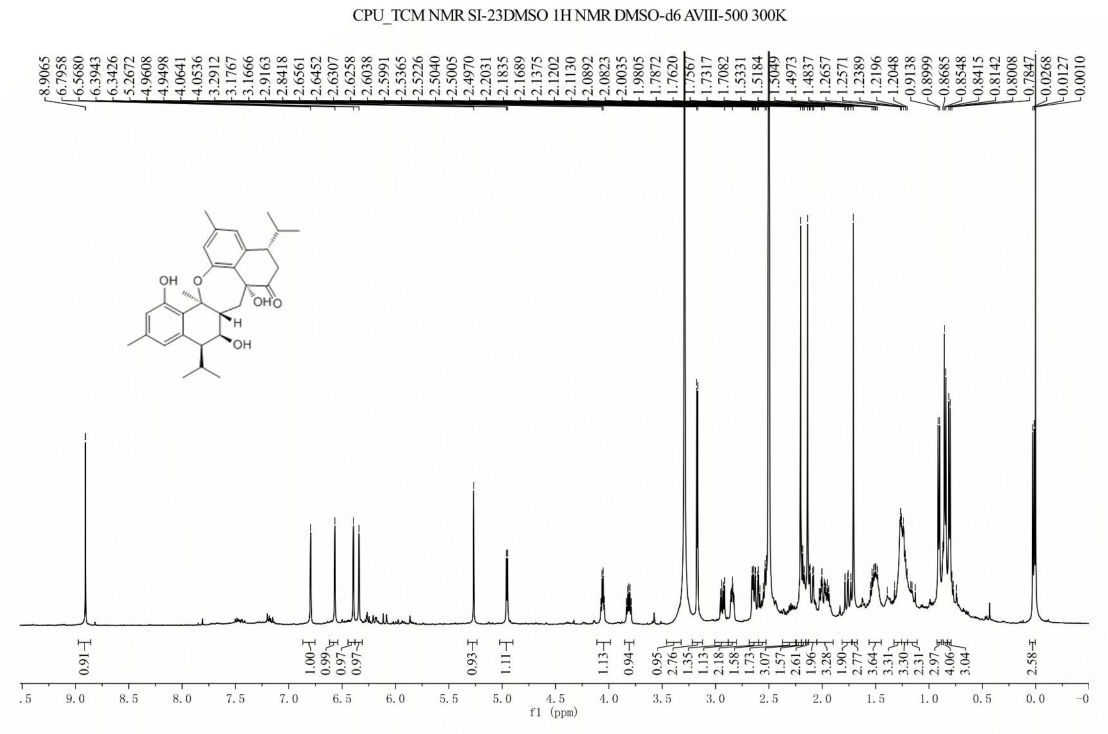

Figure S39. ^{1}H NMR spectrum of involucratusin C (**3**) in DMSO-d_6

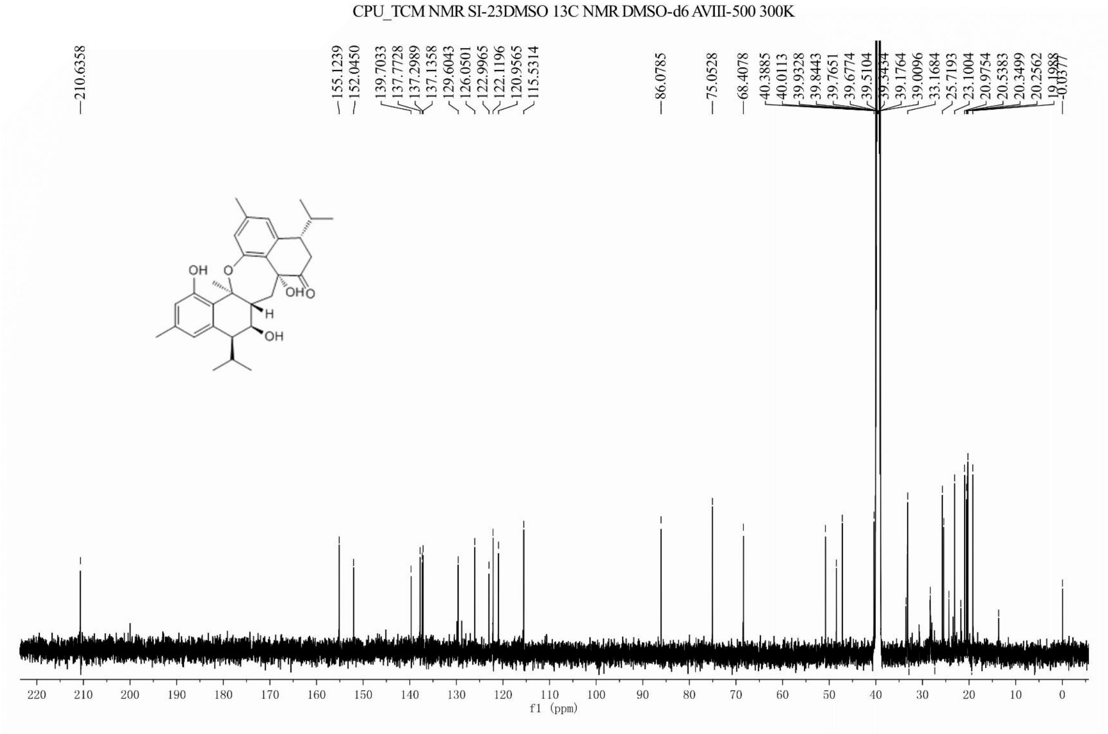

Figure S40. ^{13}C NMR spectrum of involucratusin C (**3**) in DMSO-d_6

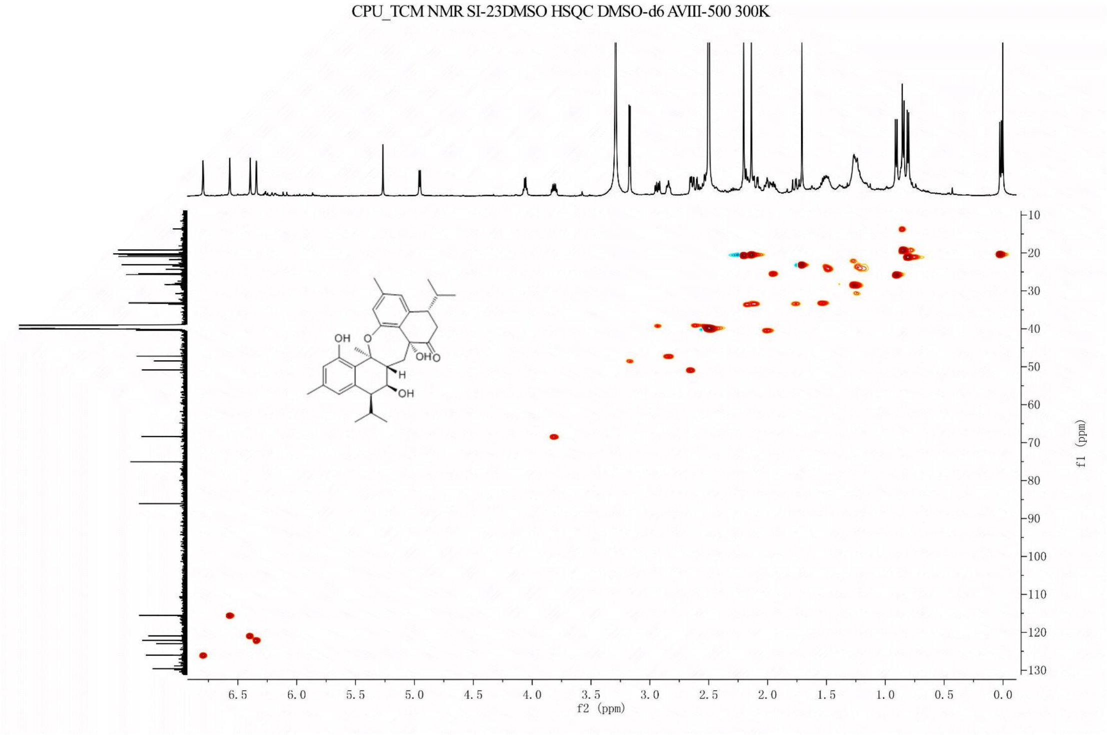

Figure S41. HSQC spectrum of involucratusin C (**3**) in DMSO-d_6

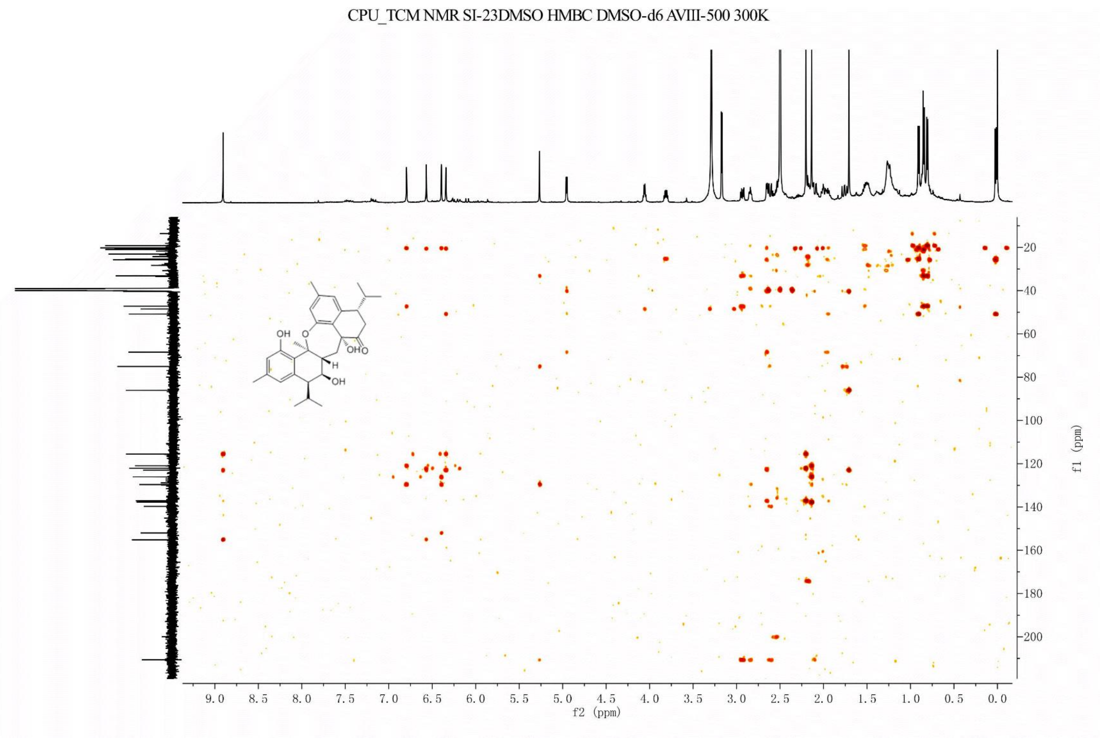

Figure S42. HMBC spectrum of involucratusin C (**3**) in DMSO-d_6

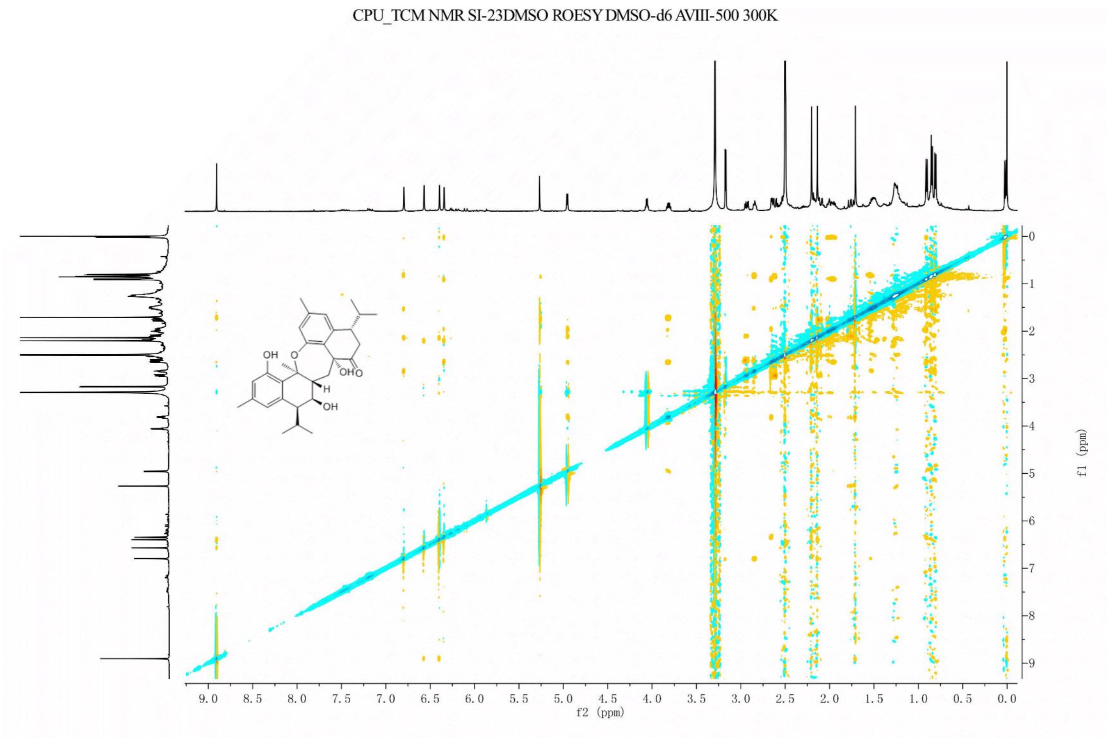

Figure S43. ROESY spectrum of involucratusin C (**3**) in DMSO-d_6

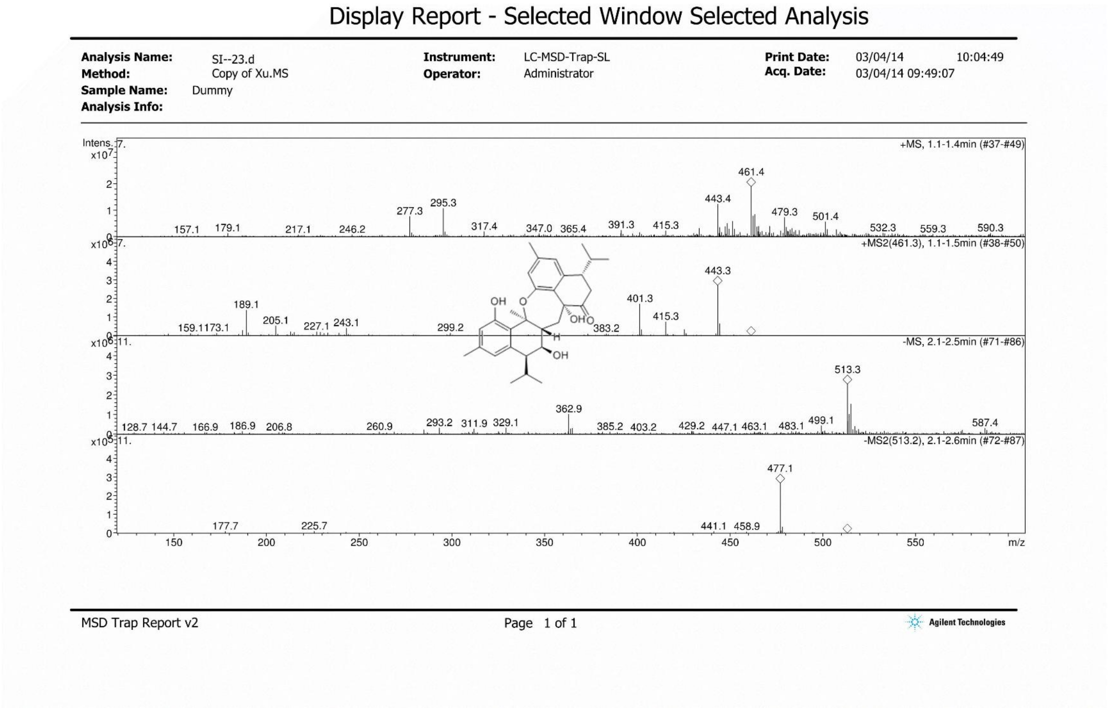

Figure S44. ESIMS spectrum of involucratusin C (**3**) in CH_3OH

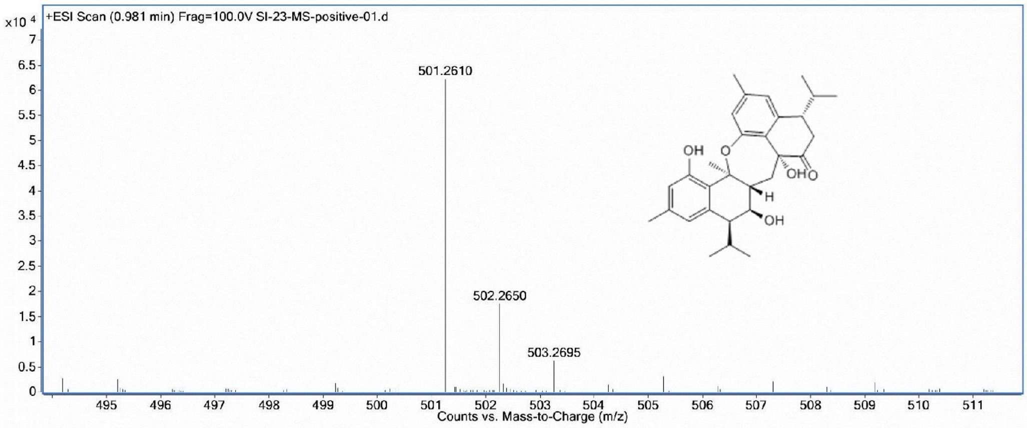

Elemental Composition Calculator

Target m/z:	501.2610	Result type:	Positive ions	Species:	[M+Na]$^{-}$
**Elements:**		C (0-80); H (0-120); O (0-30); N(0-10); Na (0-5); S (0-5)			
**Ion Formula**		**Calculated m/z**		**PPM Error**	
C30H38NaO5		501.2611		0.30	

Agilent Technologies

Figure S45. HRESIMS spectrum of involucratusin C (**3**) in CH_3OH

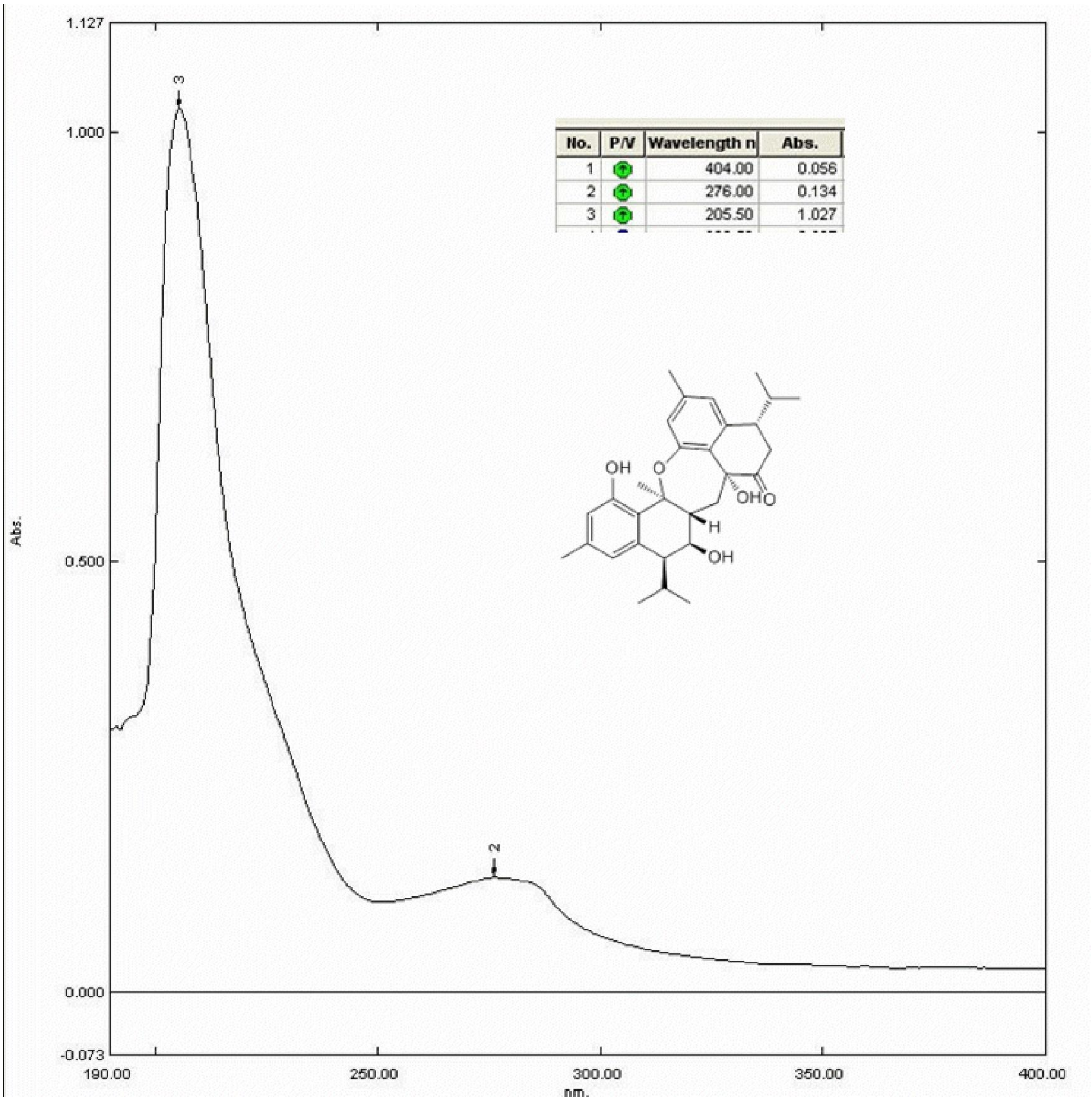

Figure S46. UV spectrum of involucratusin C (**3**) in CH_3OH

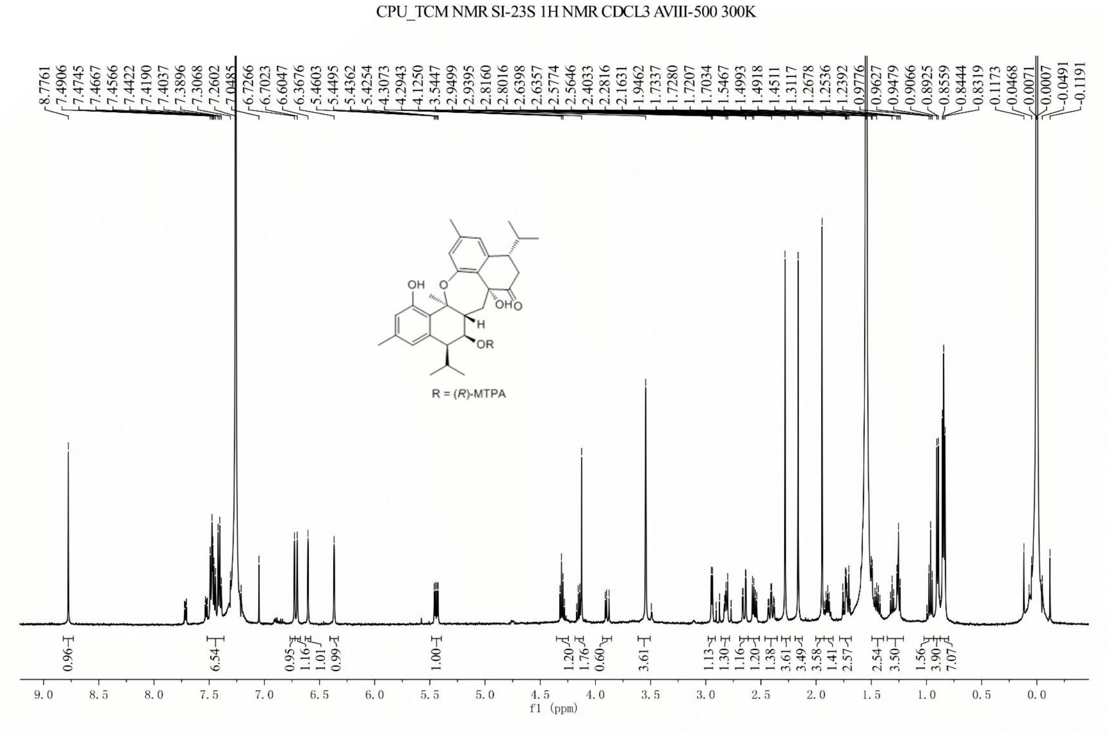

Figure S47. ^{1}H NMR spectrum of compound **3a** in $CDCl_3$

Display Report - Selected Window Selected Analysis

Analysis Name: SI--23S.d **Instrument:** amaZon SL **Print Date:** 2014-07-23 10:08:20 AM
Method: XU_MS.M **Operator:** bruker **Acq. Date:** 2014-07-23 10:04:46 AM
Sample Name: Sample
Analysis Info:

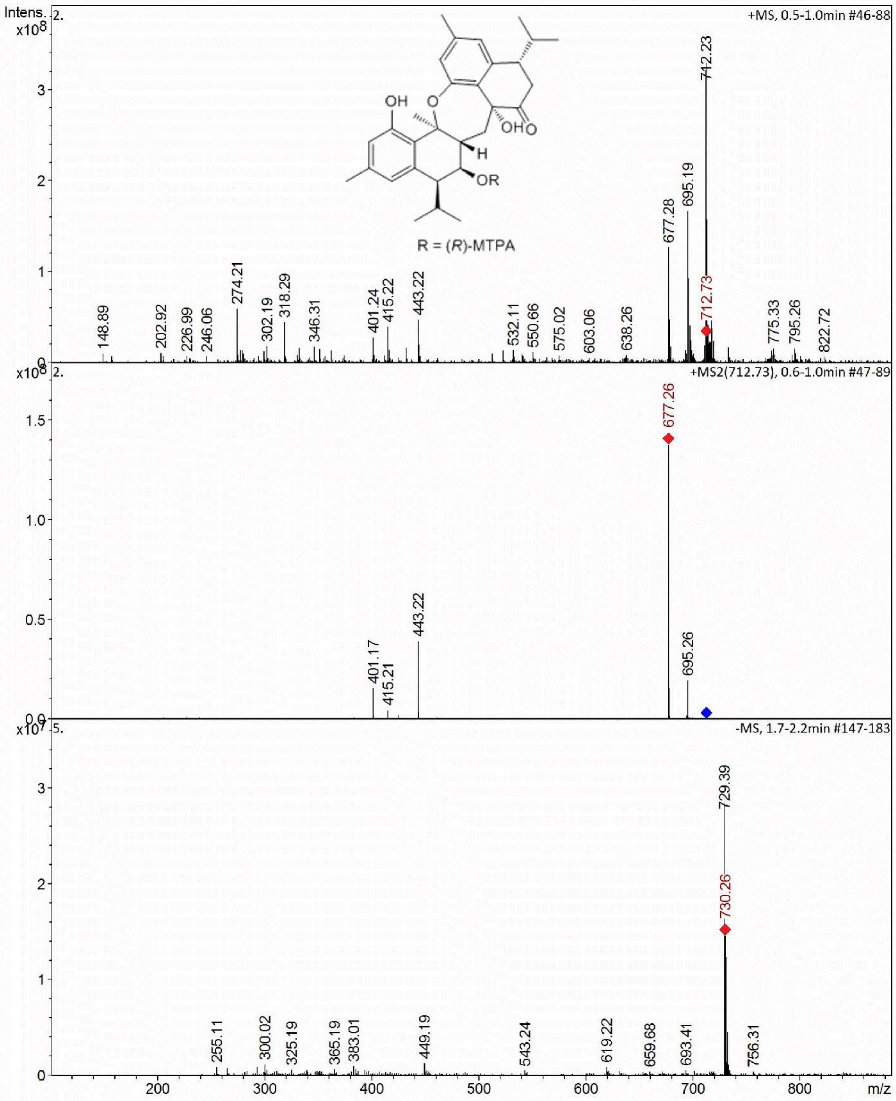

MSD Trap Report v2 Page 1 of 1

Figure S48. ESIMS spectrum of compound **3a** in CH_3OH

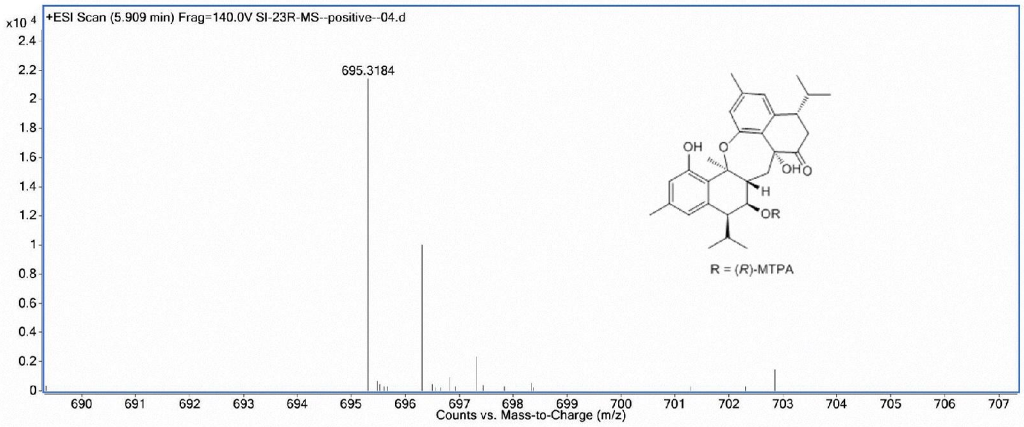

Elemental Composition Calculator

Target m/z:	695.3184	Result type:	Positive ions	Species:	$[M+H]^+$
**Elements:**		C (0-80); H (0-120); O (0-30); N(0-10); F (0-5)			
**Ion Formula**		**Calcalated m/z**		**PPM Error**	
C40H46F3O7		695.319		0.86	

Agilent Technologies

Figure S49. HRESIMS spectrum of compound **3a** in CH_3OH

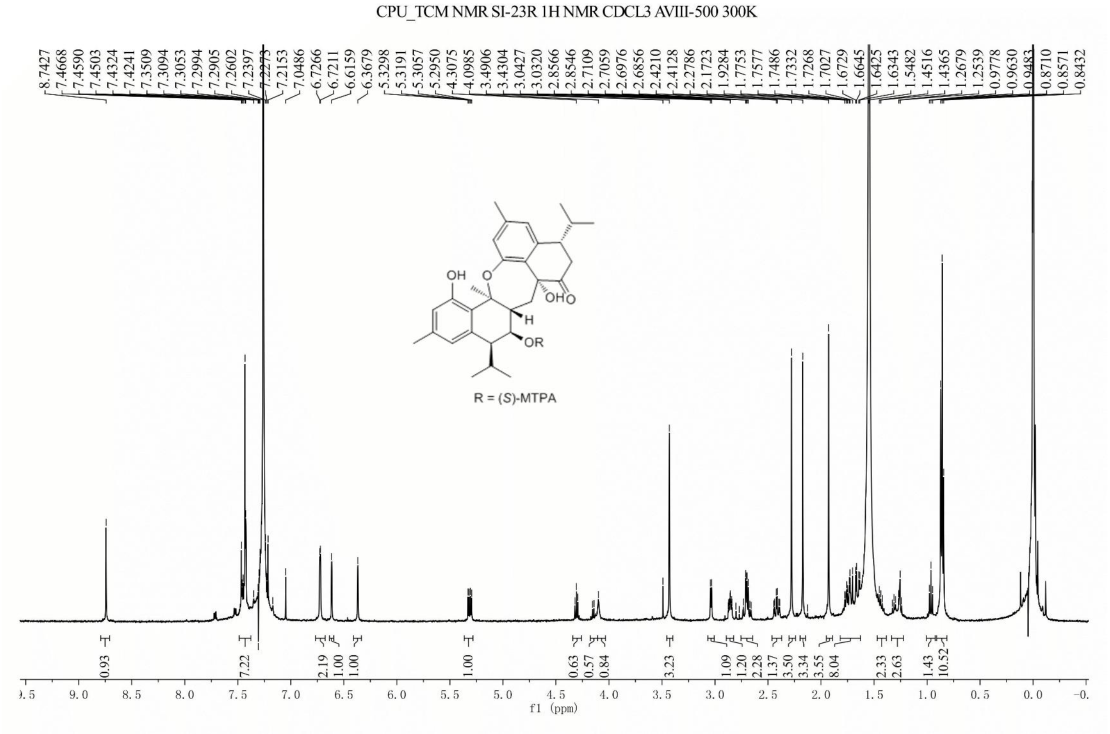

Figure S50. ^{1}H NMR spectrum of compound **3b** in $CDCl_3$

Display Report - Selected Window Selected Analysis

Analysis Name: SI--23R.d **Instrument:** amaZon SL **Print Date:** 2014-07-23 10:32:36 AM
Method: XU_MS.M **Operator:** bruker **Acq. Date:** 2014-07-23 10:27:58 AM
Sample Name: Sample
Analysis Info:

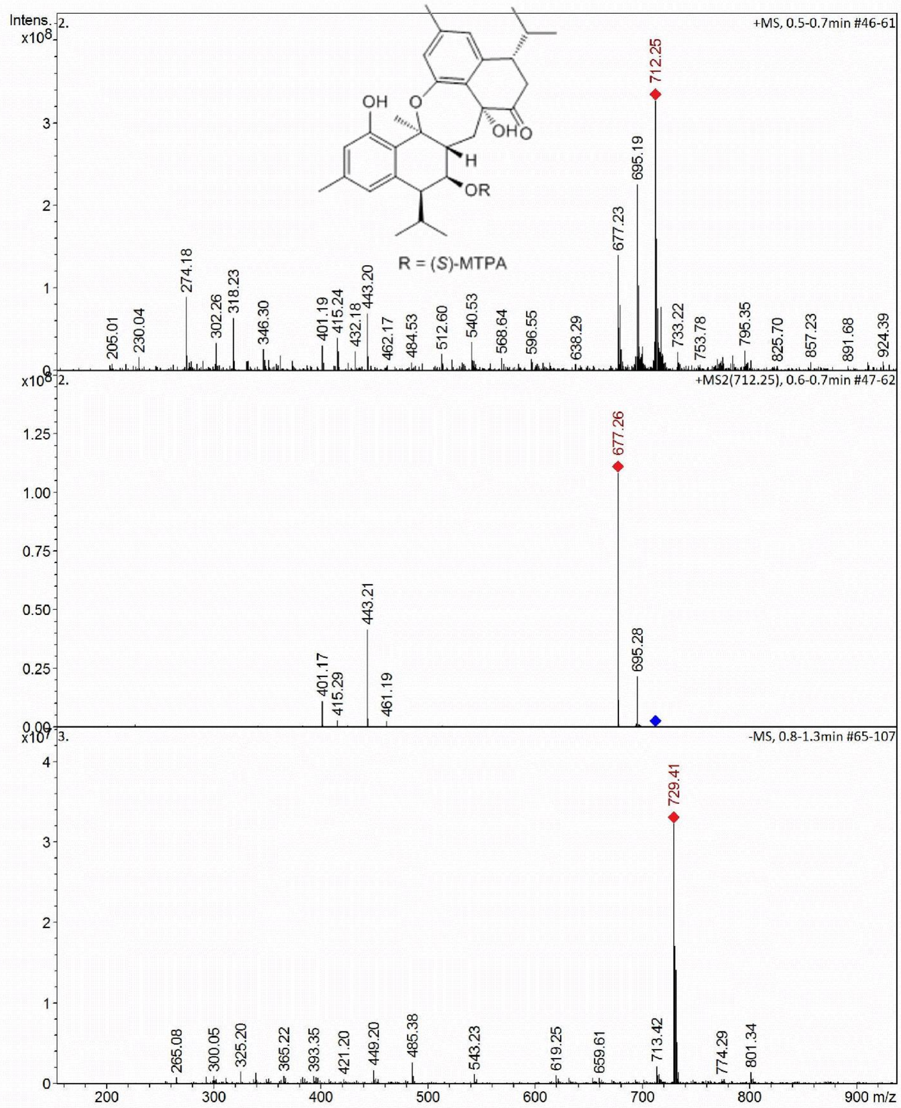

MSD Trap Report v2 Page 1 of 1

Figure S51. ESIMS spectrum of compound **3b** in CH_3OH

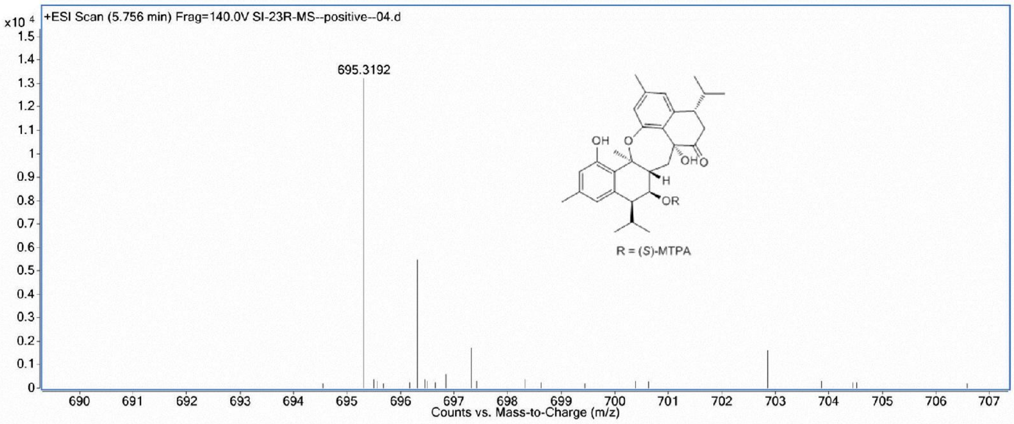

Elemental Composition Calculator

**Target m/z:**	695.3192	**Result type:**	Positive ions	**Species:**	$[M+H]^+$
**Elements:**		C (0-80); H (0-120); O (0-30); N(0-10); Na (0-5)			
**Ion Formula**		**Calcalated m/z**		**PPM Error**	
C40H46F3O7		695.319		-0.34	

Agilent Technologies

Figure S52. HRESIMS spectrum of compound **3b** in CH_3OH

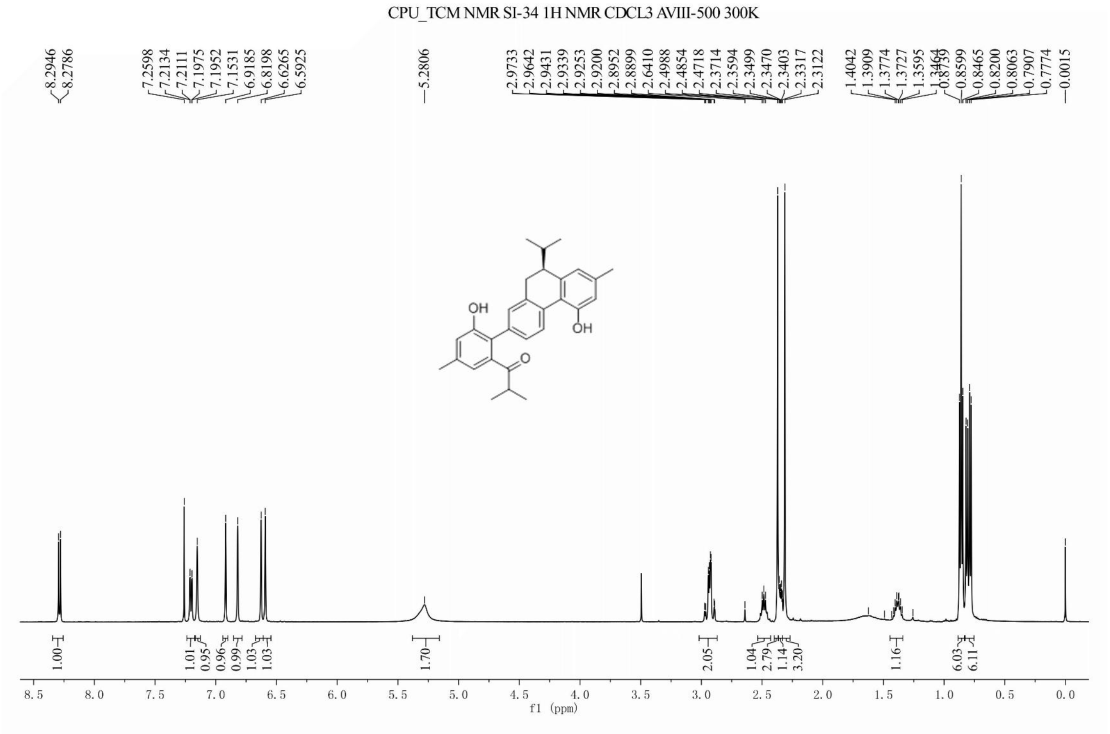

Figure S53. ^{1}H NMR spectrum of involucratusin D (**4**) in $CDCl_3$

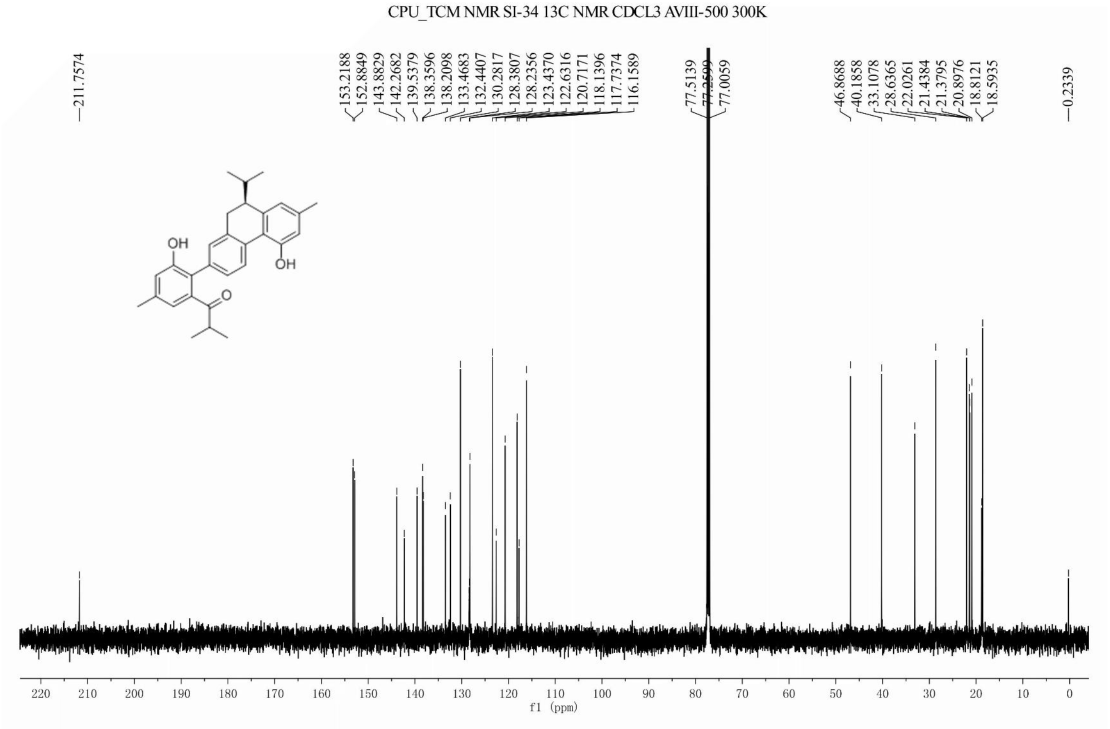

Figure S54. ^{13}C NMR spectrum of involucratusin D (**4**) in $CDCl_3$

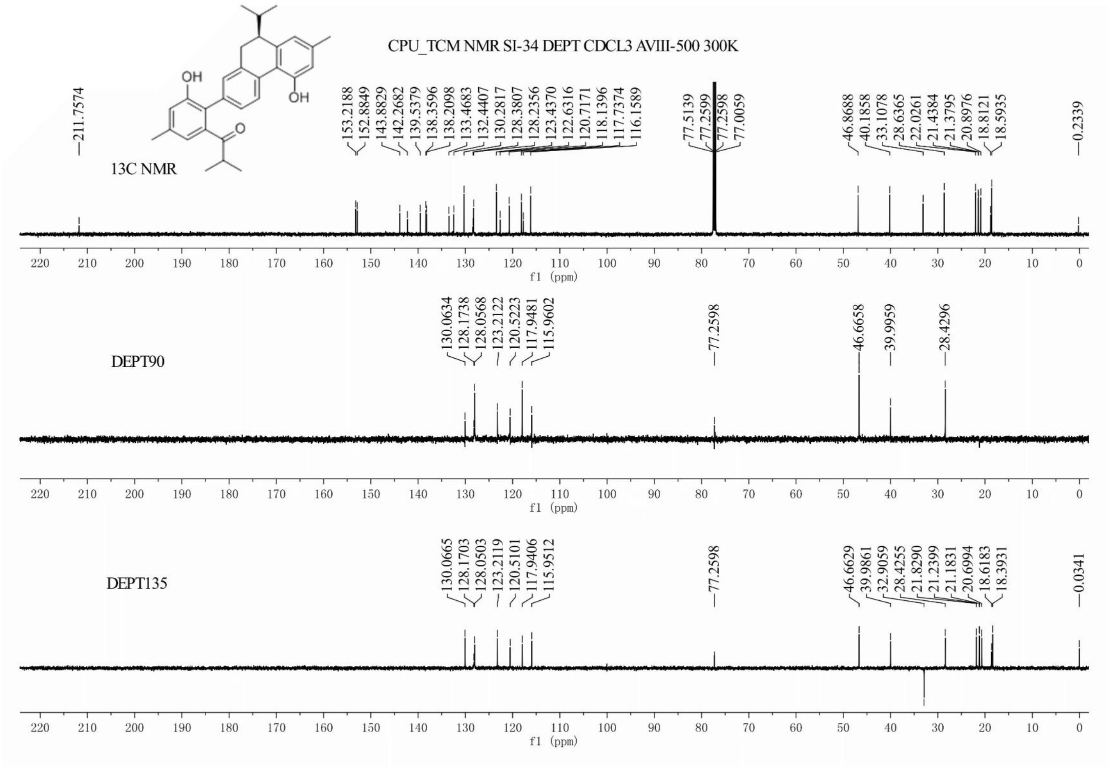

Figure S55. DEPT spectrum of involucratusin D (**4**) in $CDCl_3$

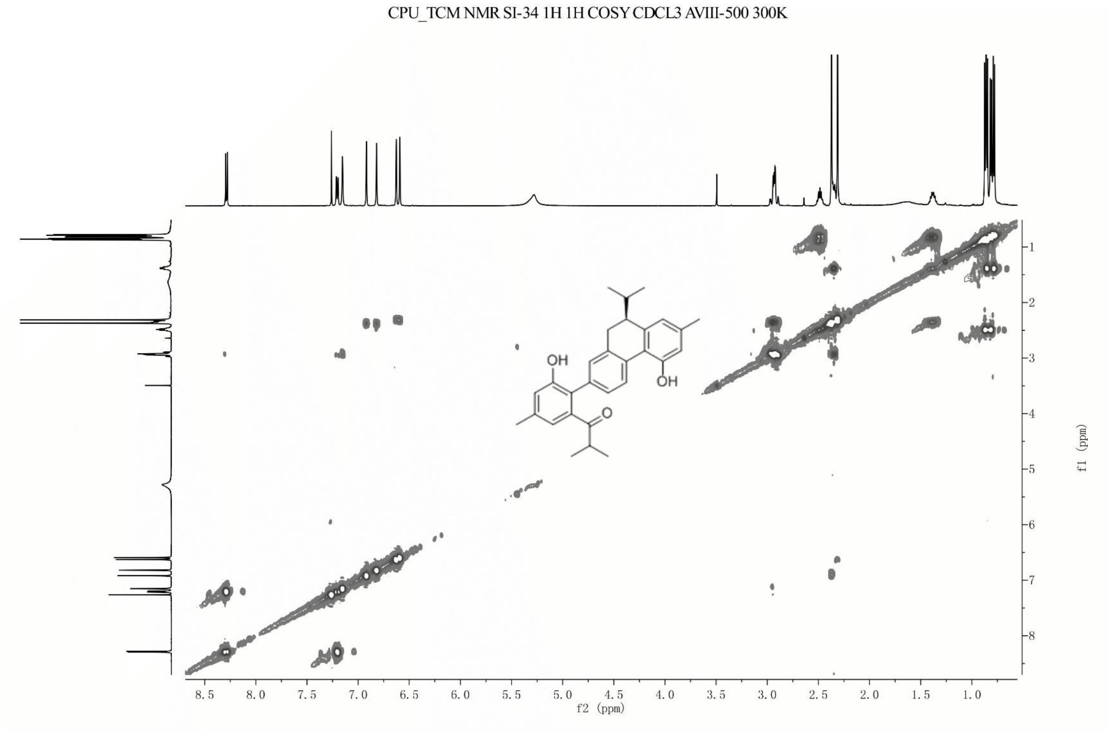

Figure S56. COSY spectrum of involucratusin D (**4**) in $CDCl_3$

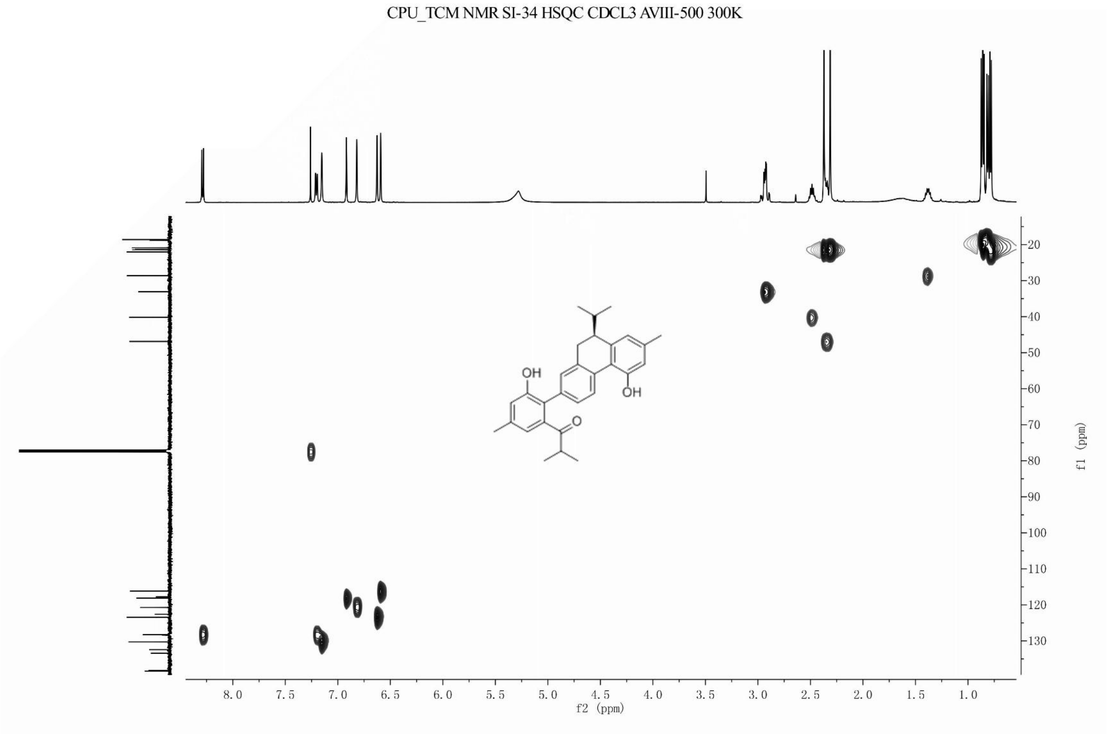

Figure S57. HSQC spectrum of involucratusin D (**4**) in $CDCl_3$

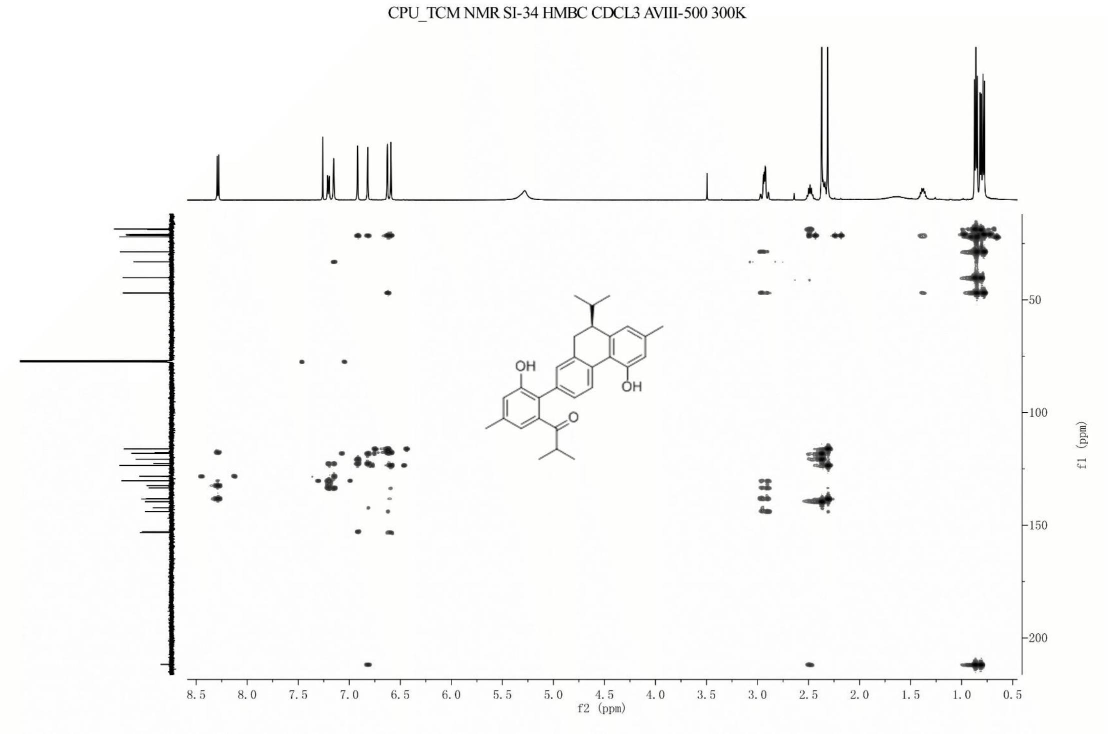

Figure S58. HMBC spectrum of involucratusin D (**4**) in $CDCl_3$

CPU_TCM NMR SI-34 ROESY CDCL3 AVIII-500 300K

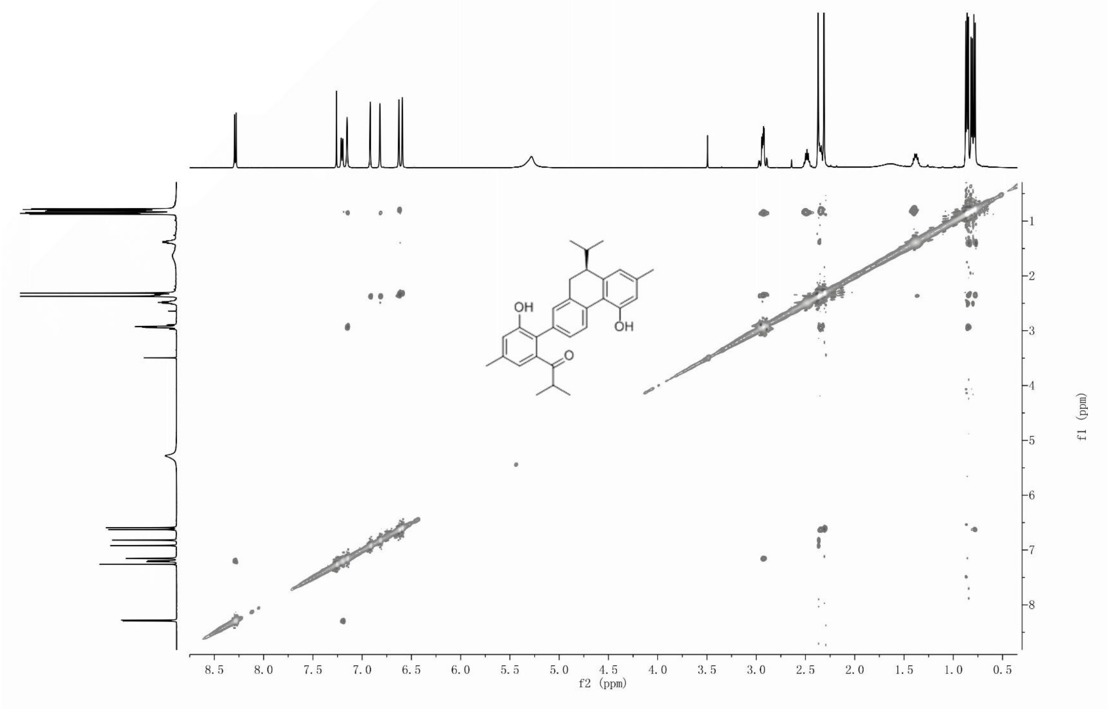

Figure S59. ROESY spectrum of involucratusin D (**4**) in $CDCl_3$

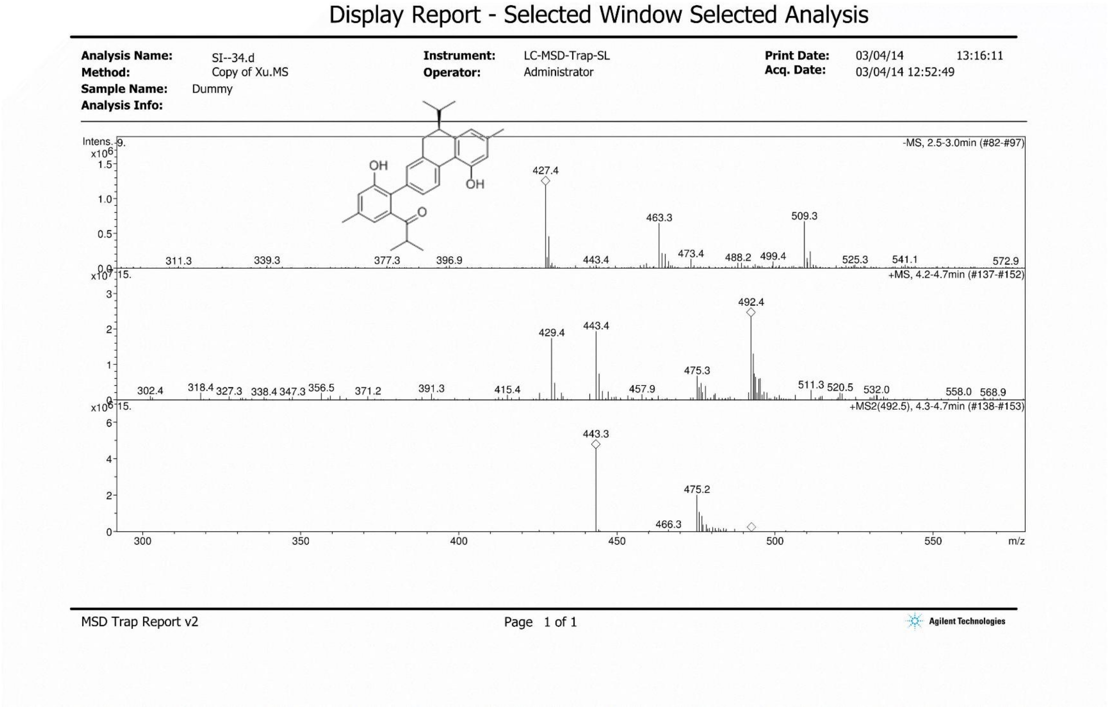

Figure S60. ESIMS spectrum of involucratusin D (**4**) in CH_3OH

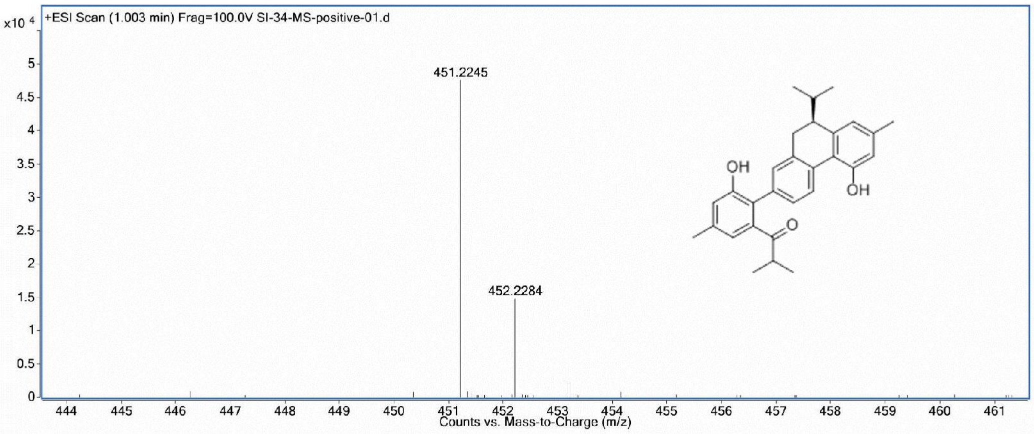

Elemental Composition Calculator

Target m/z:	451.2245	Result type:	Positive ions	Species:	$[M+Na]^+$
Elements:		C (0-80); H (0-120); O (0-30); N(0-10); Na (0-5); S (0-5)			
Ion Formula		Calculated m/z		PPM Error	
C29H32NaO3		451.2244		-0.25	

Agilent Technologies

Figure S61. HRESIMS spectrum of involucratusin D (**4**) in CH_3OH

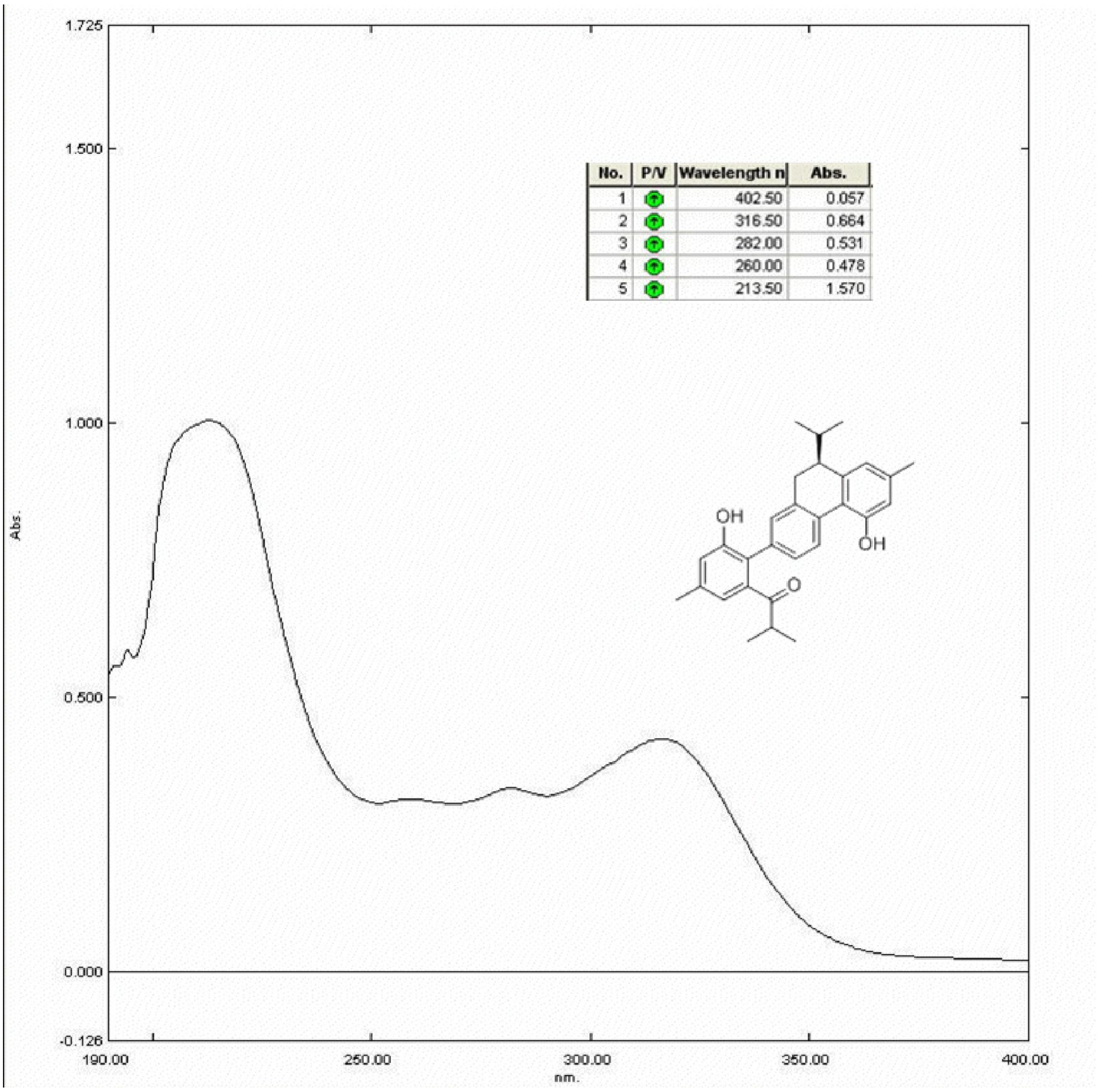

Figure S62. UV spectrum of involucratusin D (**4**) in CH_3OH

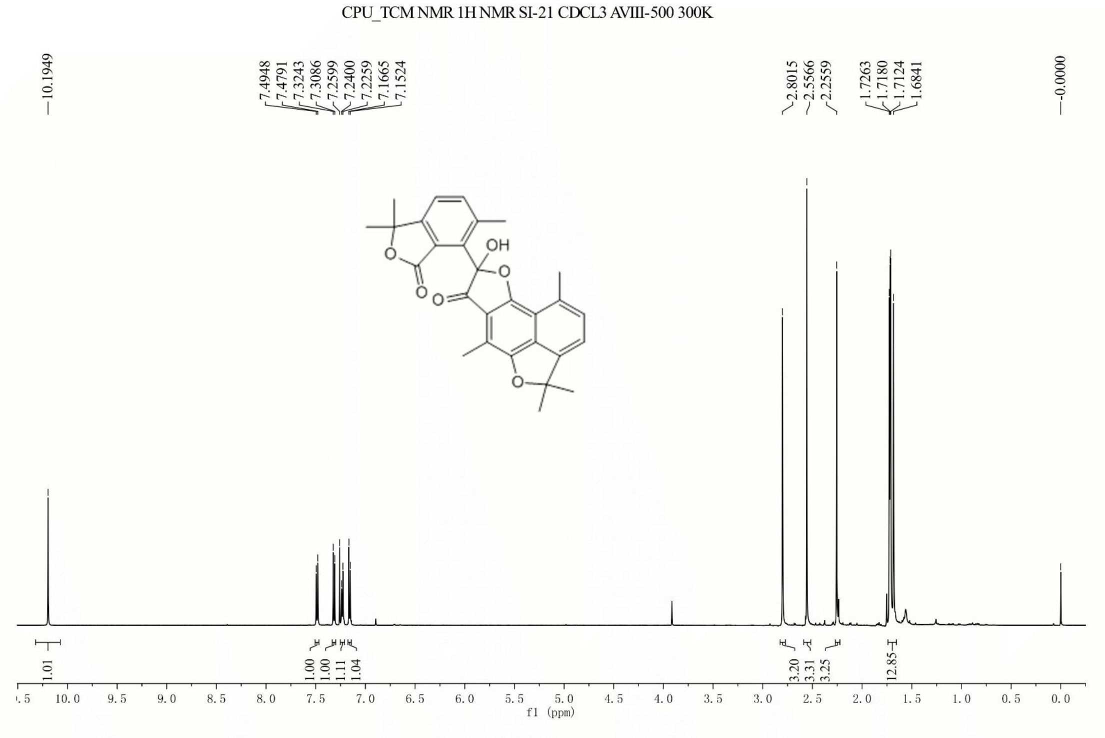

Figure S63. ^{1}H NMR spectrum of involucratusin E (**5**) in $CDCl_3$

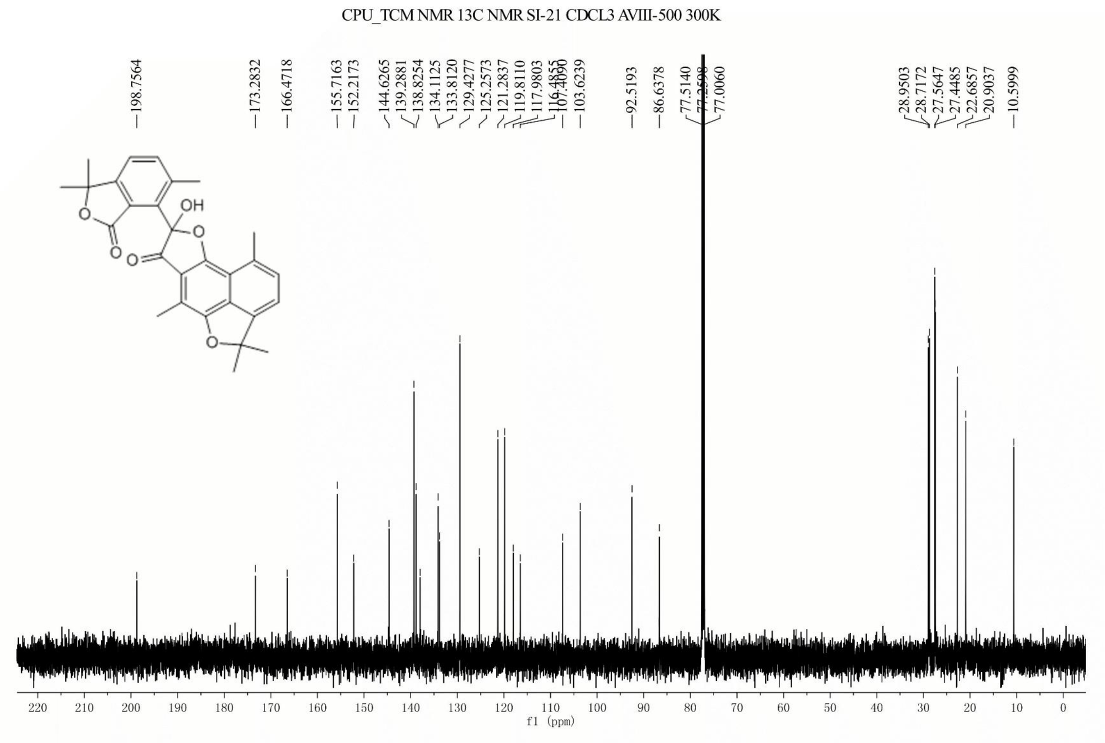

Figure S64. ^{13}C NMR spectrum of involucratusin E (**5**) in $CDCl_3$

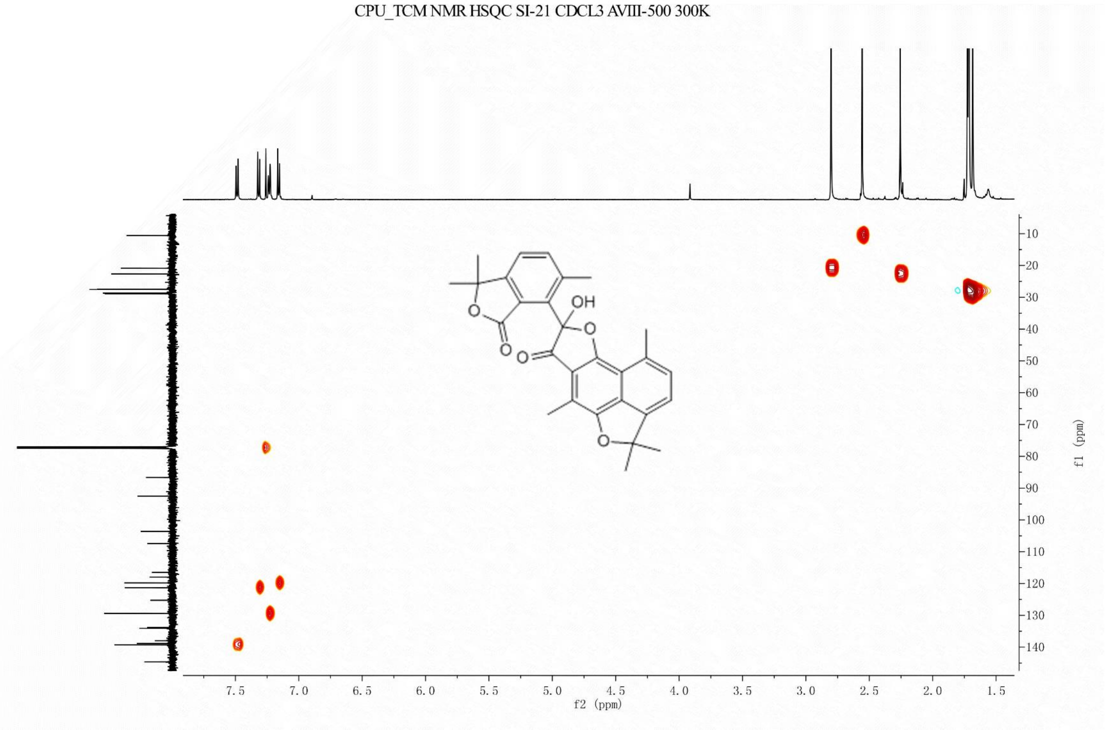

Figure S65. HSQC spectrum of involucratusin E (**5**) in $CDCl_3$

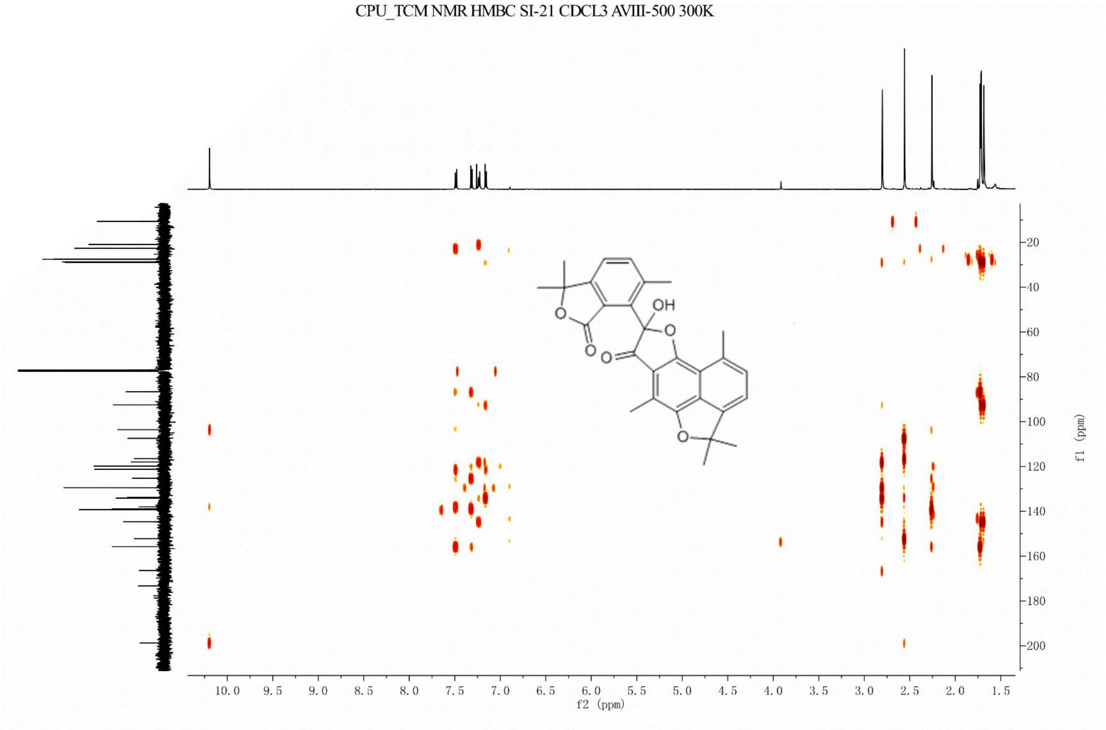

Figure S66. HMBC spectrum of involucratusin E (**5**) in $CDCl_3$

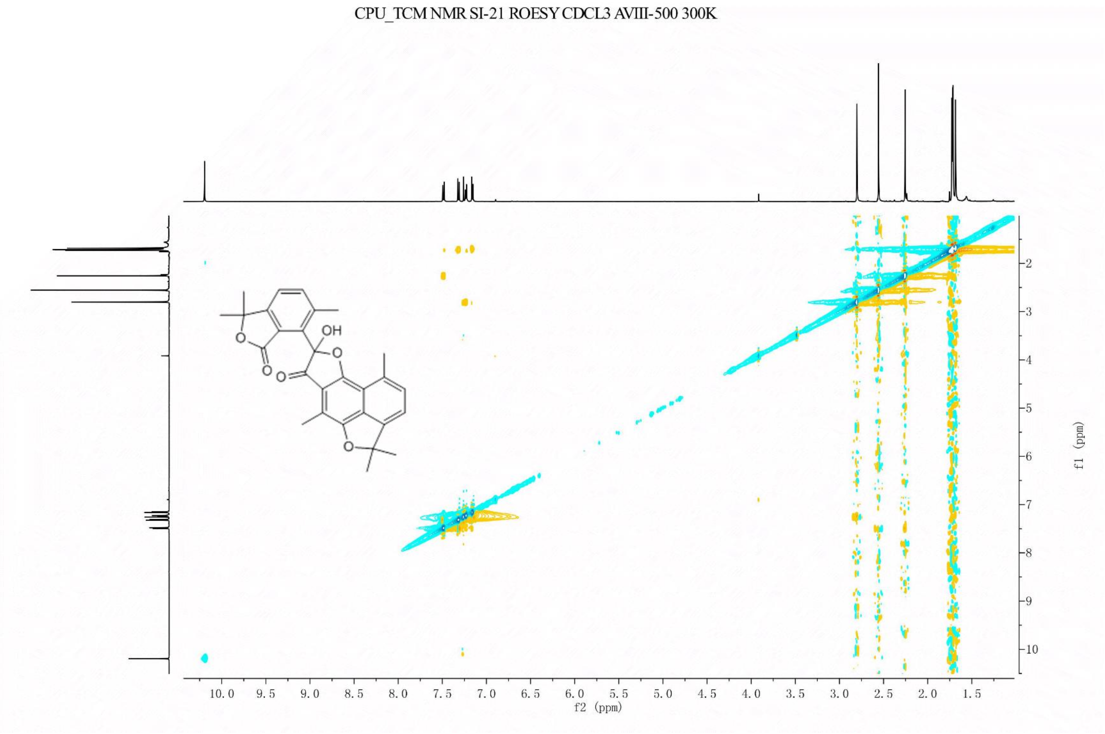

Figure S67. ROESY spectrum of involucratusin E (**5**) in $CDCl_3$

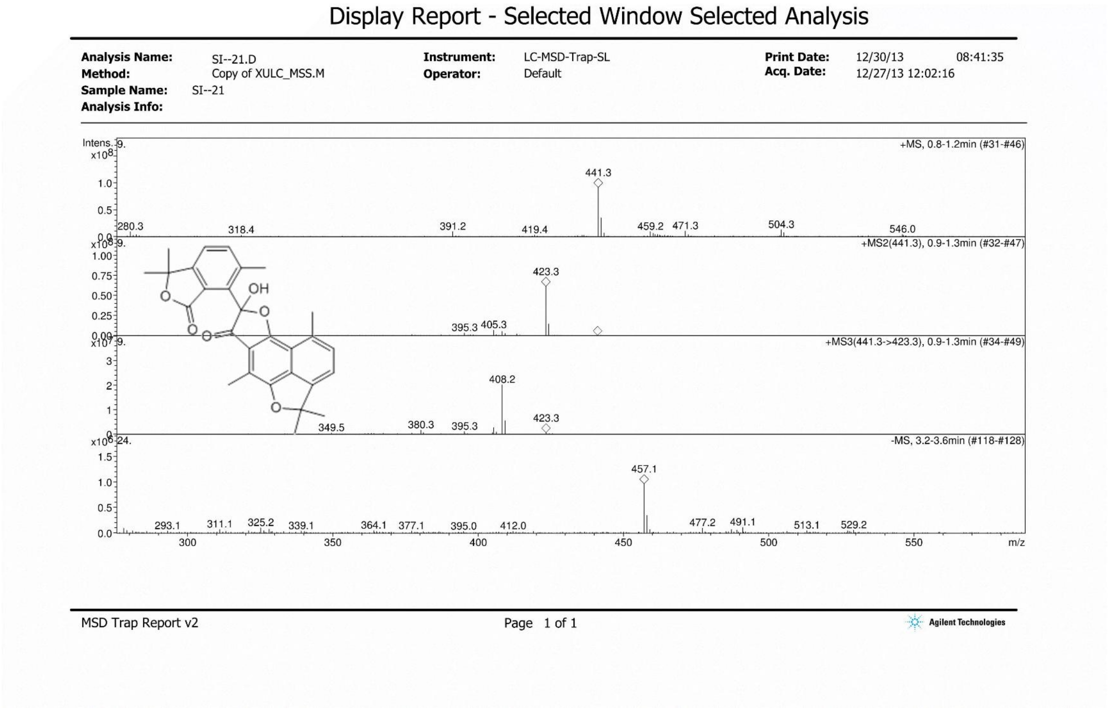

Figure S68. ESIMS spectrum of involucratusin E (**5**) in CH_3OH

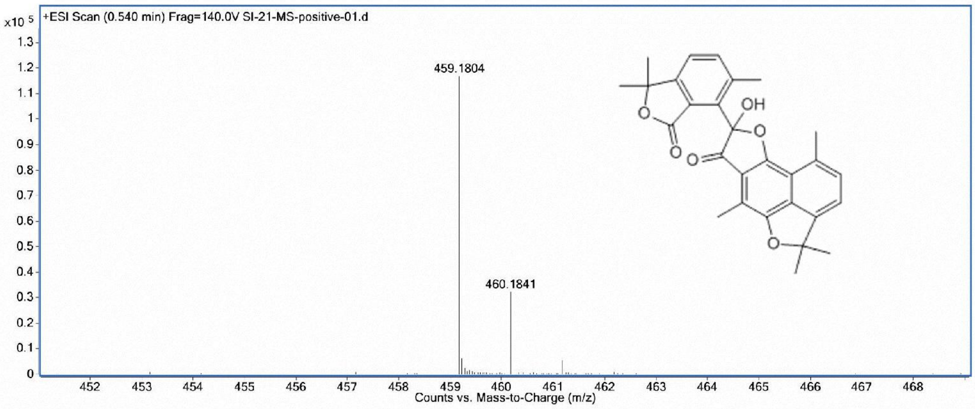

Elemental Composition Calculator

**Target m/z:**	459.1804	**Result type:**	Positive ions	**Species:**	[M+H]$^{-}$
**Elements:**		C (0-80); H (0-120); O (0-30); N(0-10); Na (0-5); S (0-5)			
**Ion Formula**		**Calculated m/z**		**PPM Error**	
C28H27O6		459.1802		-0.36	

Agilent Technologies

Figure S69. HRESIMS spectrum of involucratusin E (**5**) in CH_3OH

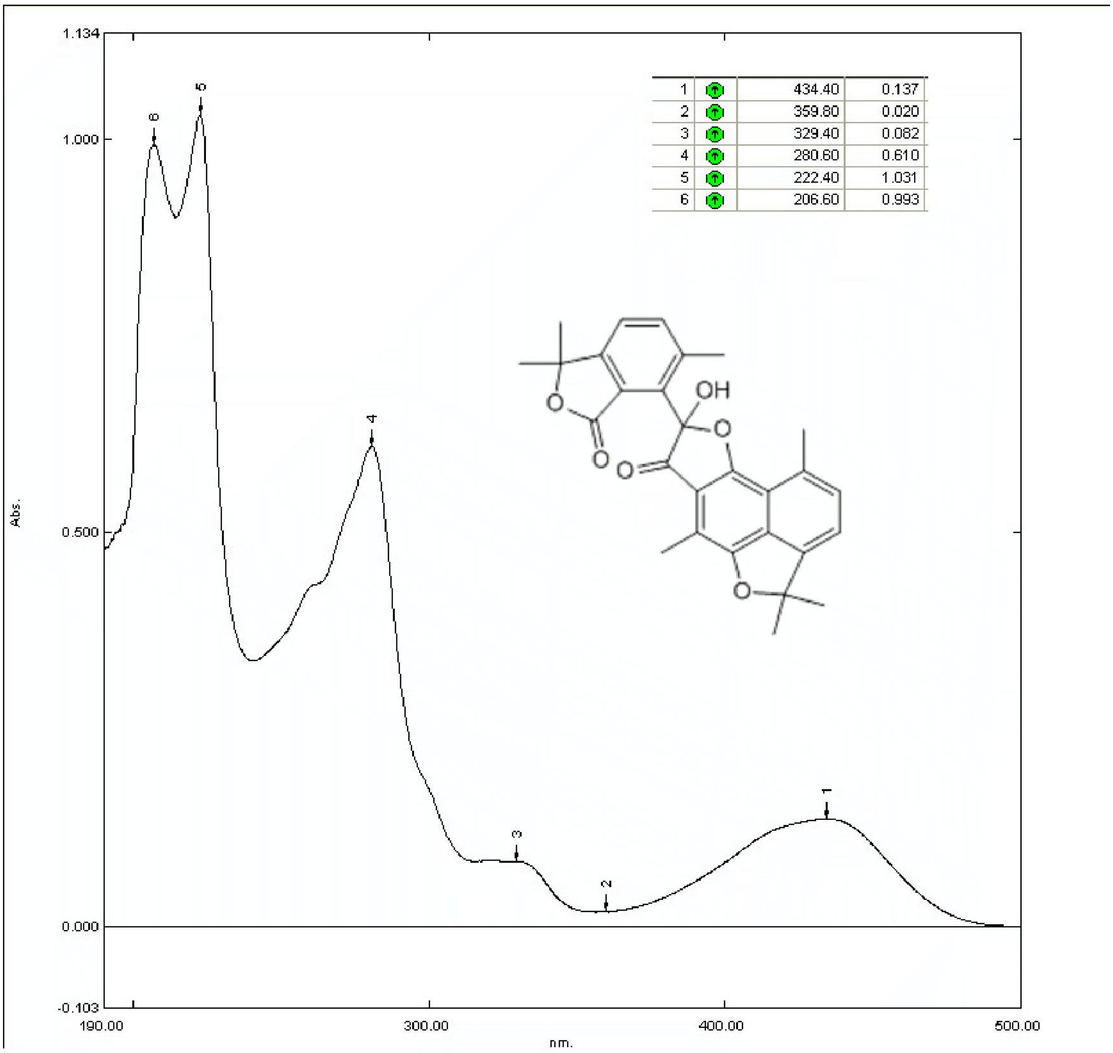

Figure S70. UV spectrum of involucratusin E (**5**) in CH_3OH

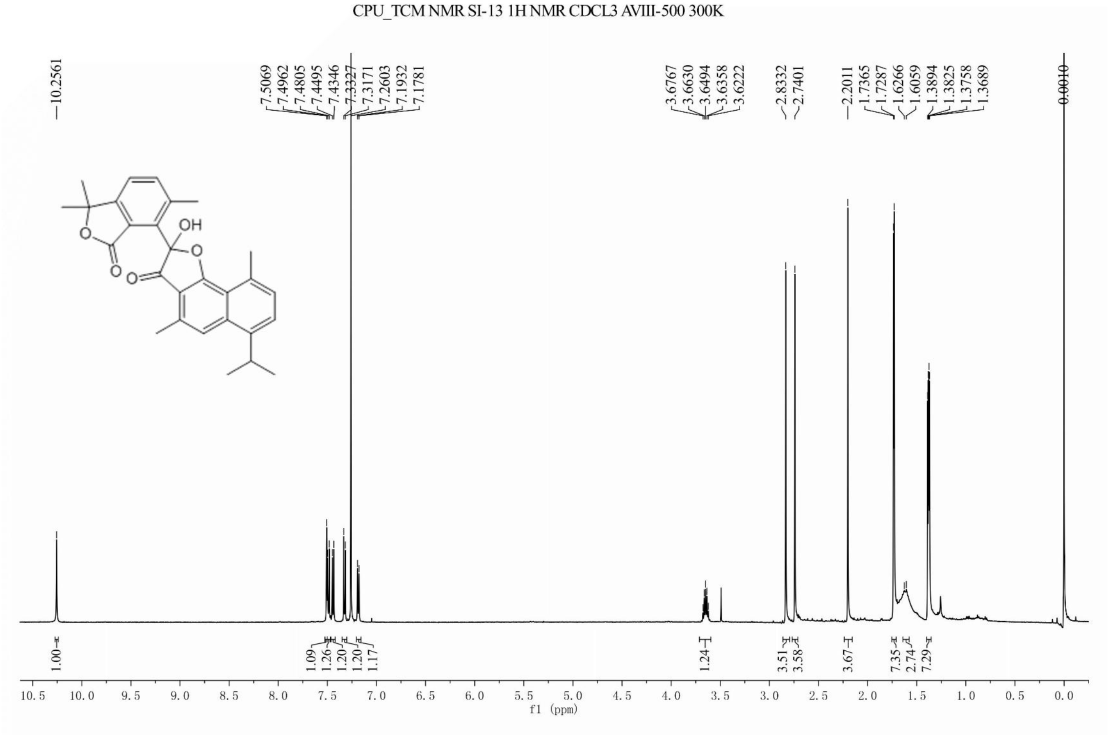

Figure S71. ^{1}H NMR spectrum of involucratusin F (**6**) in $CDCl_3$

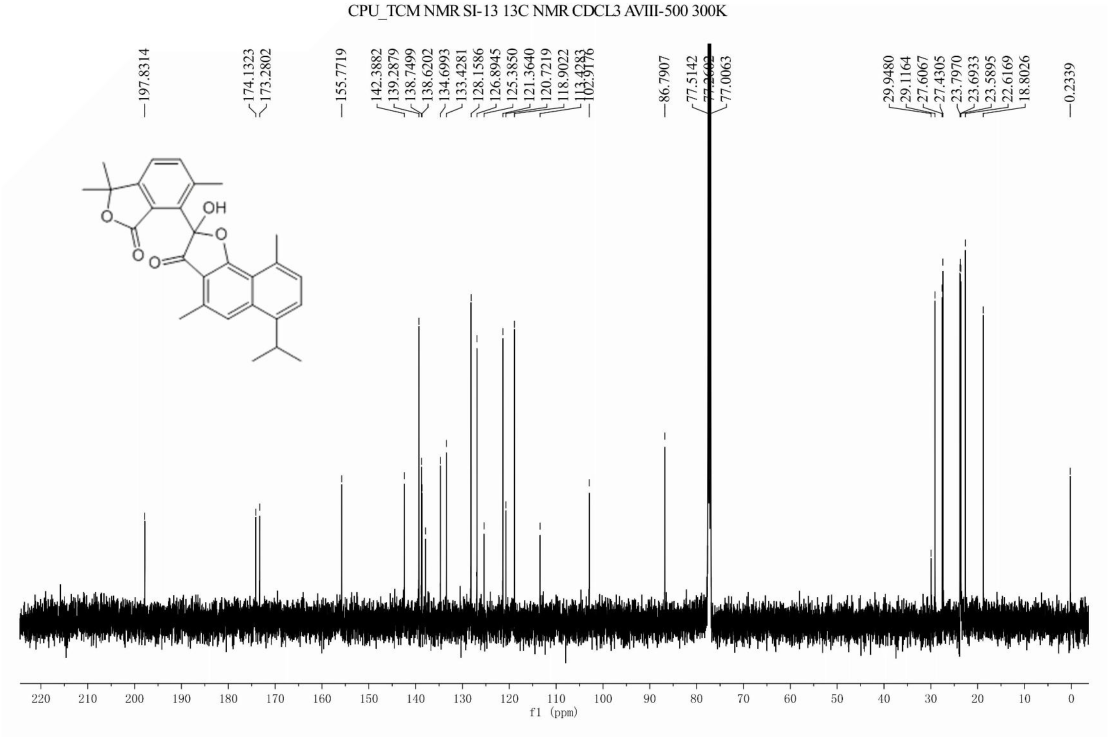

Figure S72. ^{13}C NMR spectrum of involucratusin F (**6**) in $CDCl_3$

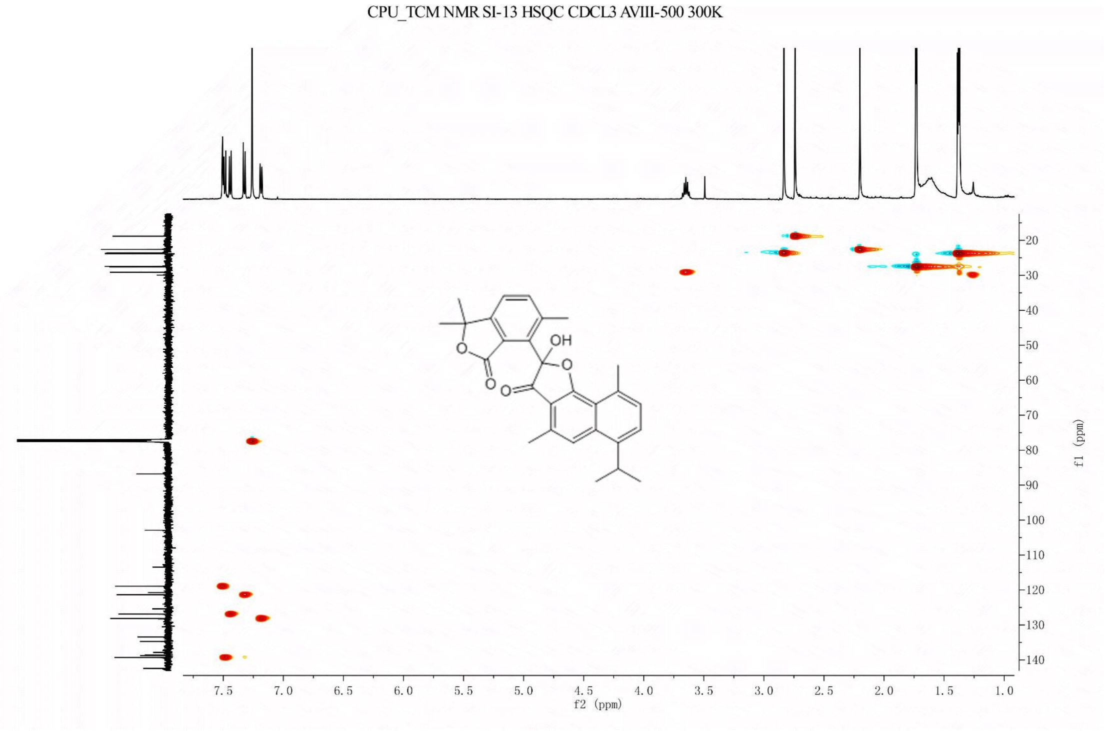

Figure S73. HSQC spectrum of involucratusin F (**6**) in $CDCl_3$

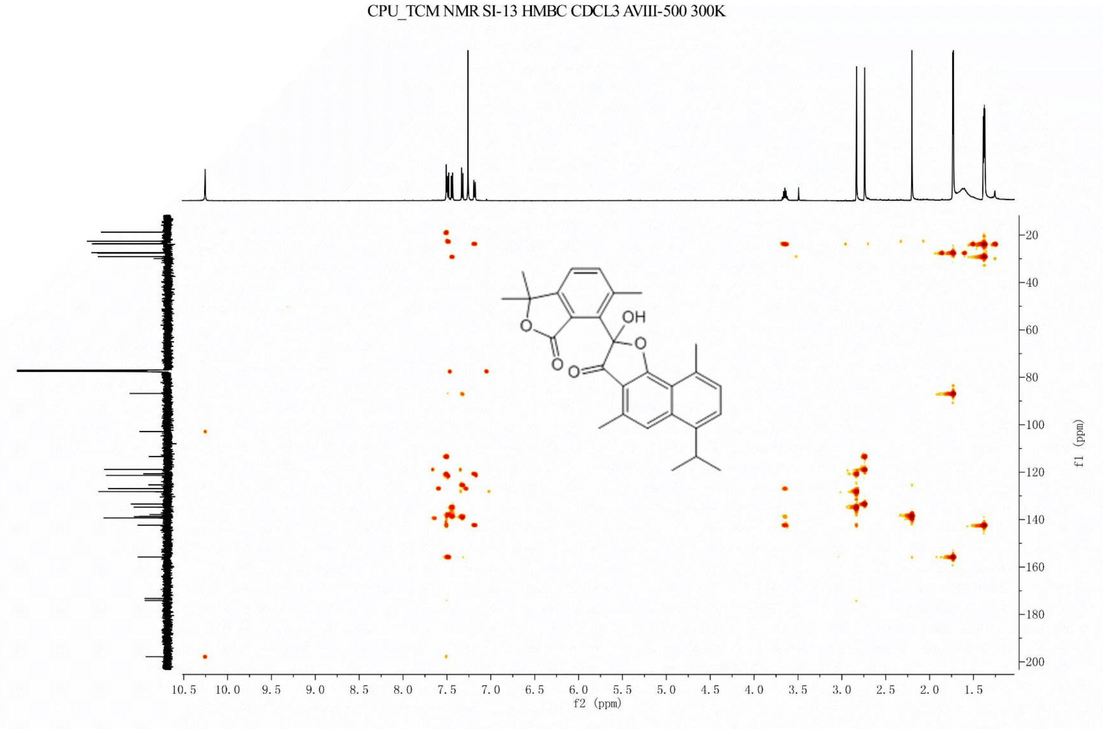

Figure S74. HMBC spectrum of involucratusin F (**6**) in $CDCl_3$

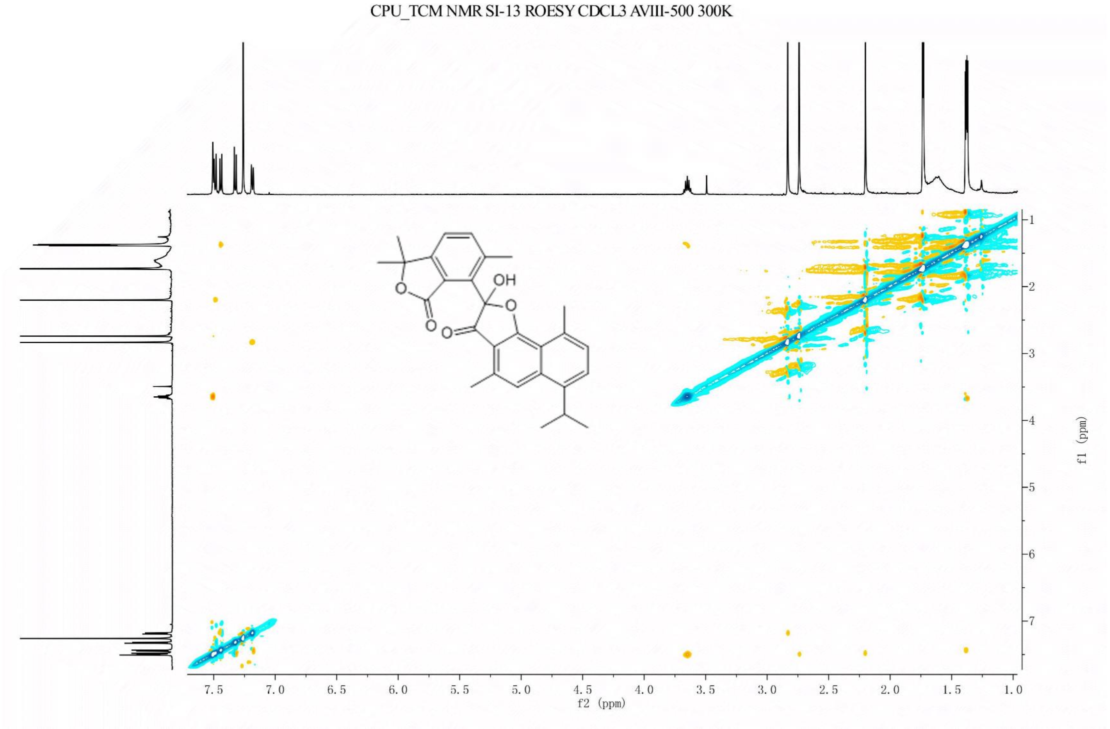

Figure S75. ROESY spectrum of involucratusin F (**6**) in $CDCl_3$

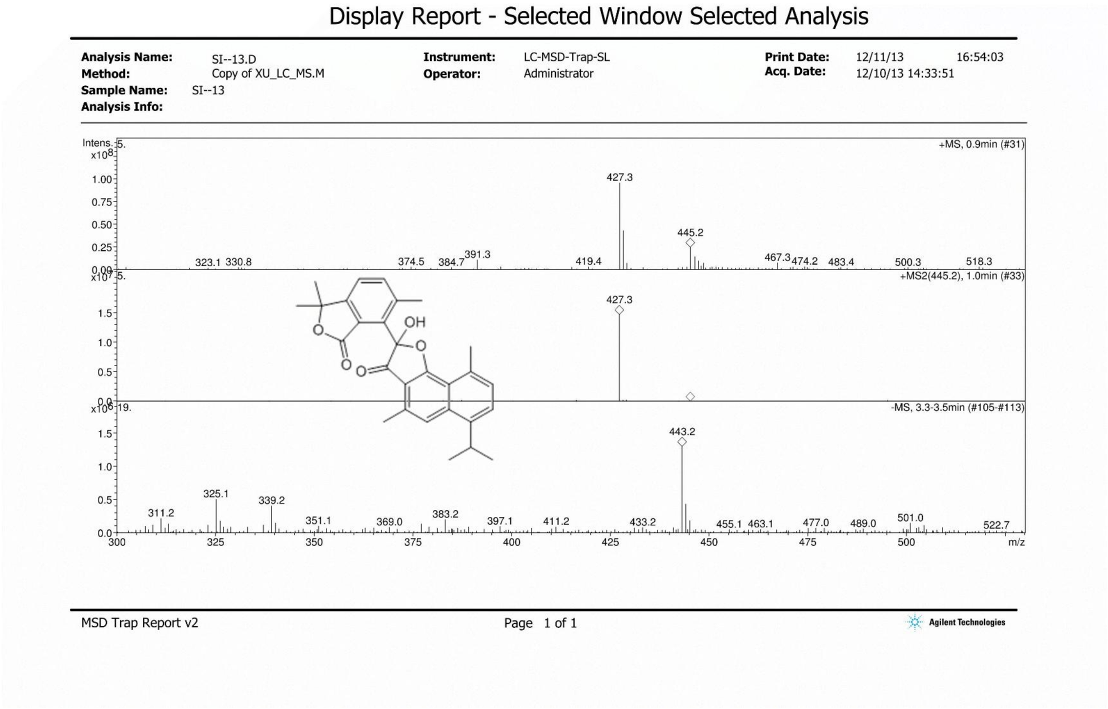

Figure S76. ESIMS spectrum of involucratusin F (**6**) in CH_3OH

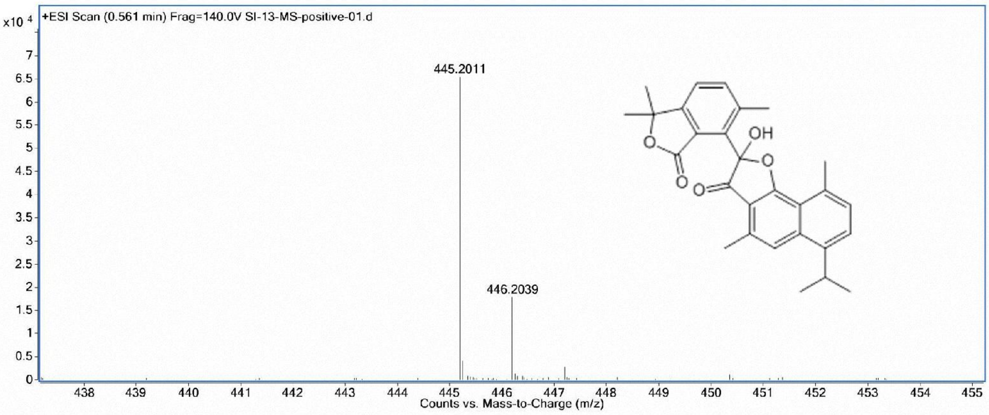

Elemental Composition Calculator

Target m/z:	445.2011	Result type:	Positive ions	Species:	$[M+H]^+$
**Elements:**		C (0-80); H (0-120); O (0-30); N(0-10); Na (0-5); S (0-5)			
**Ion Formula**		**Calculated m/z**		**PPM Error**	
C28H29O5		445.2010		-0.26	

Agilent Technologies

Figure S77. HRESIMS spectrum of involucratusin F (**6**) in CH_3OH

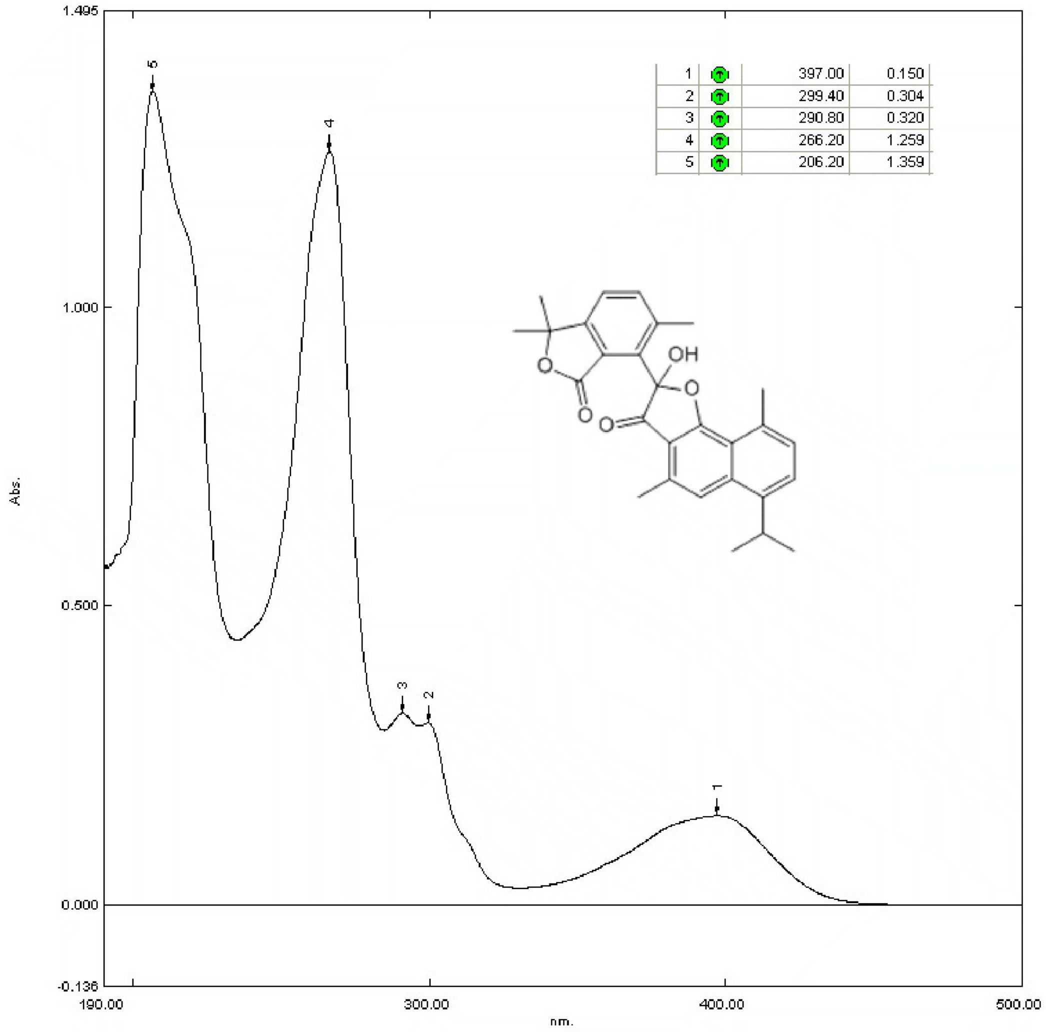

Figure S78. UV spectrum of involucratusin F (**6**) in CH_3OH

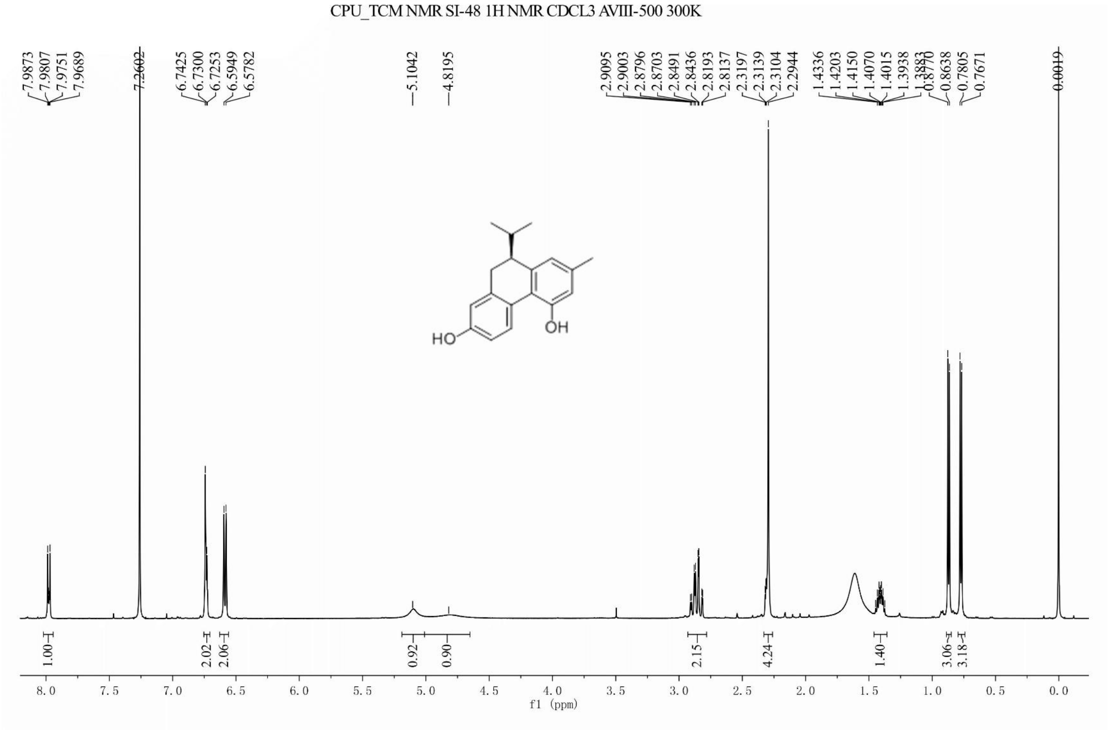

Figure S79. ^{1}H NMR spectrum of involucratusin G (**7**) in $CDCl_3$

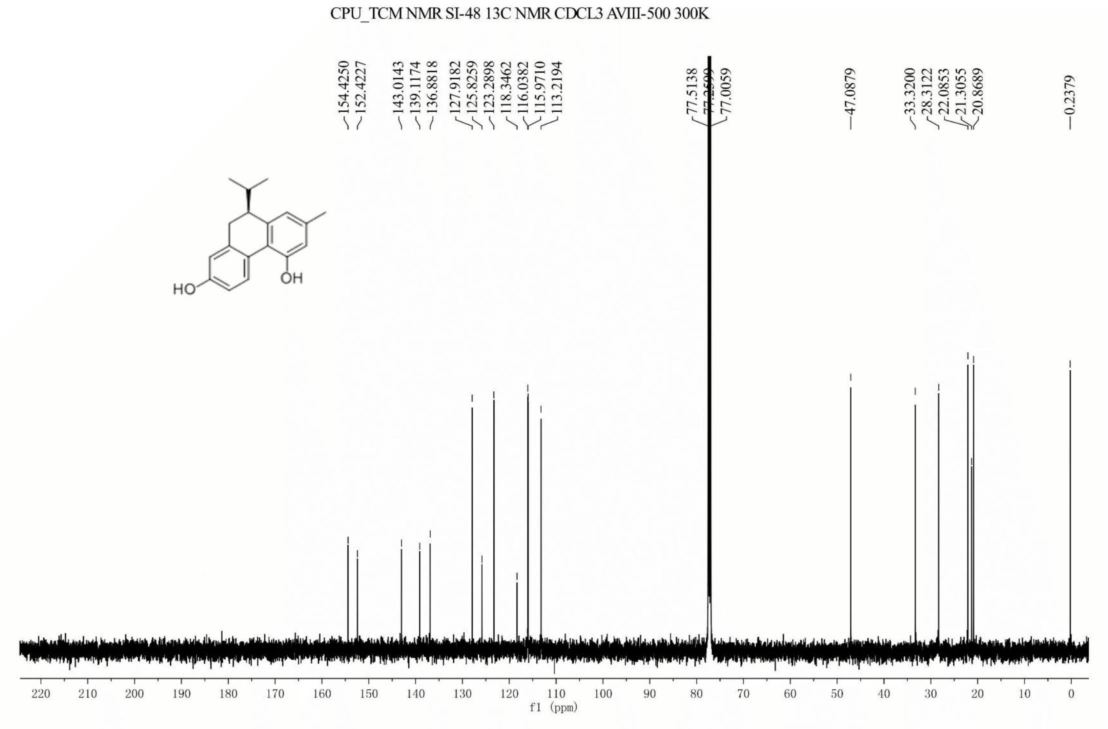

Figure S80. ^{13}C NMR spectrum of involucratusin G (**7**) in $CDCl_3$

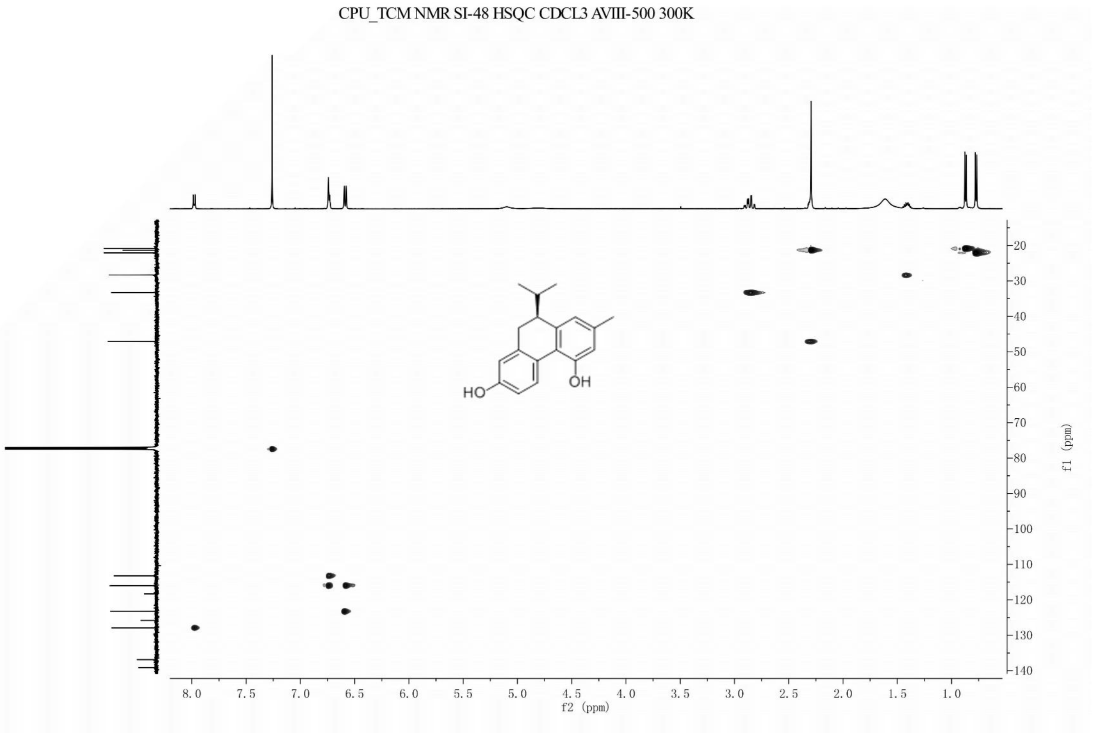

Figure S81. HSQC spectrum of involucratusin G (**7**) in $CDCl_3$

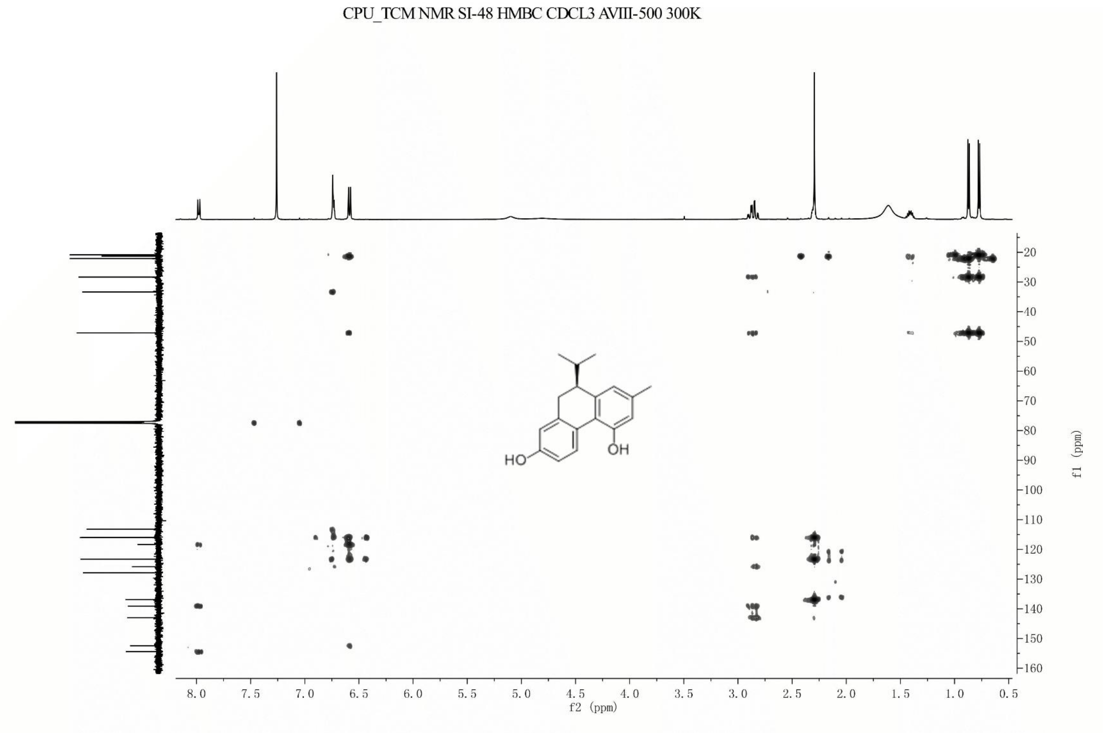

Figure S82. HMBC spectrum of involucratusin G (**7**) in $CDCl_3$

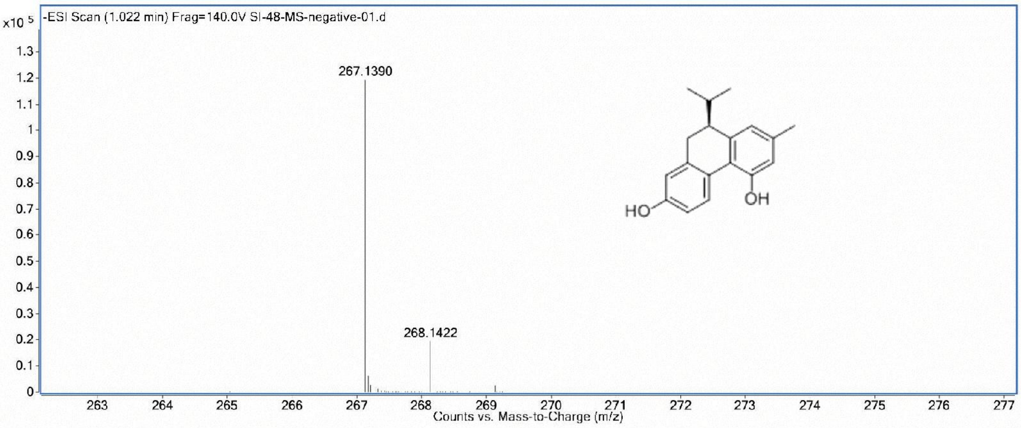

Elemental Composition Calculator

**Target m/z:**	267.1390	**Result type:**	Negative ions	**Species:**	$[M-H]^-$
**Elements:**		C (0-80); H (0-120); O (0-30); N(0-10); Cl (0-5)			
**Ion Formula**		**Calcalated m/z**		**PPM Error**	
C18H19O2		267.1391		0.05	

Agilent Technologies

Figure S83. HRESIMS spectrum of involucratusin G (**7**) in CH_3OH

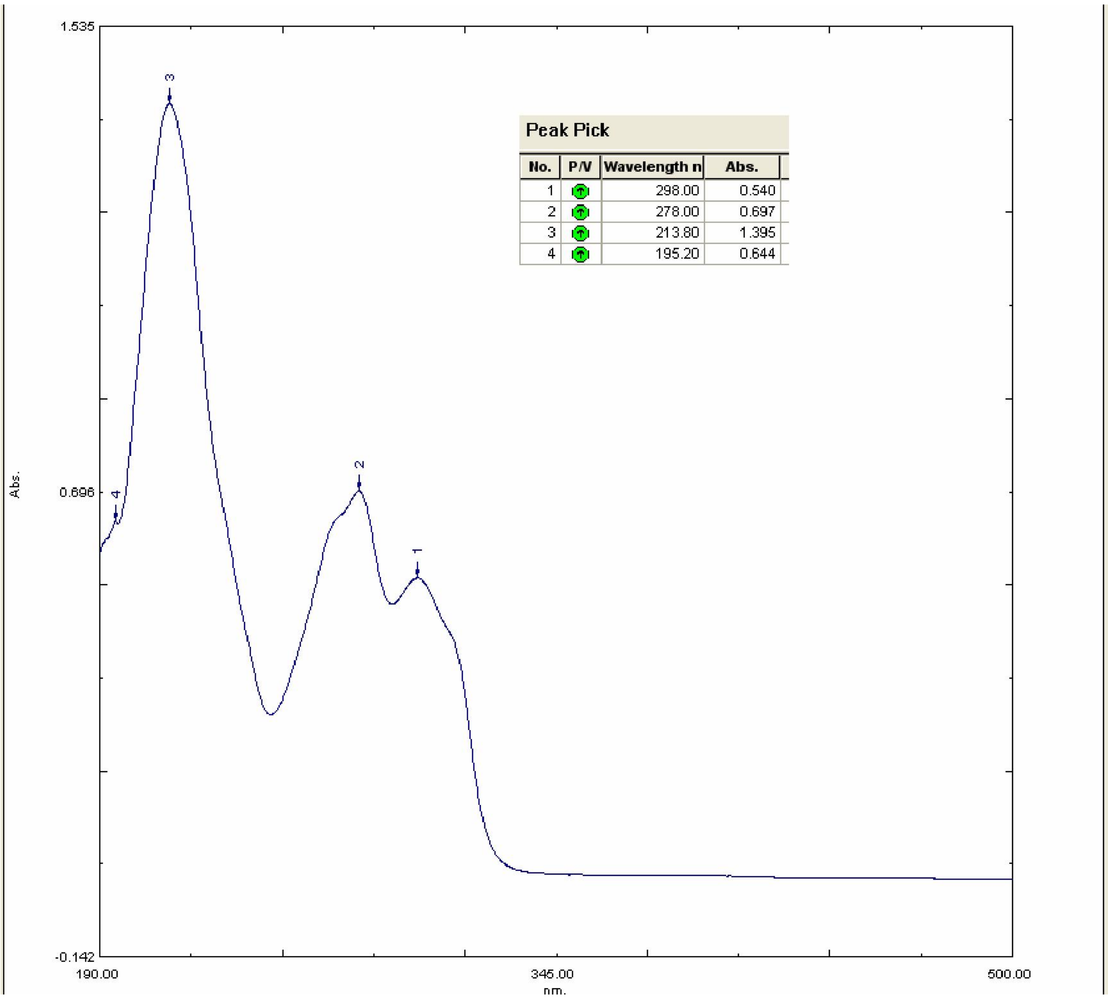

Figure S84. UV spectrum of involucratusin G (**7**) in CH_3OH

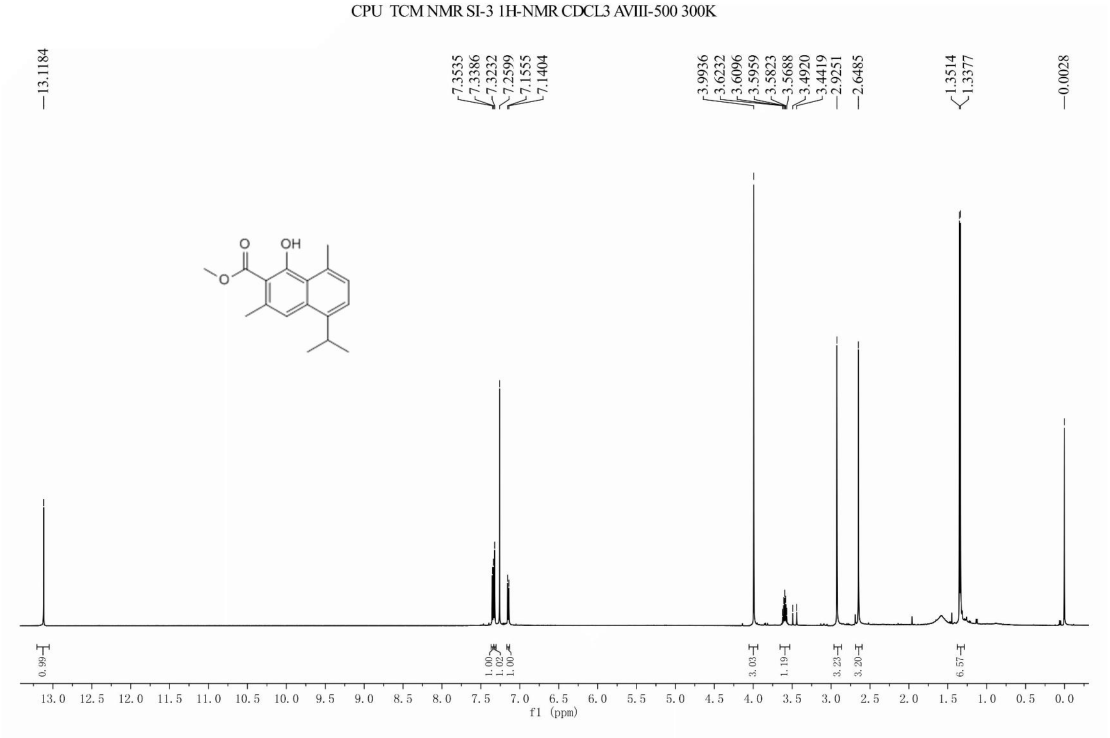

Figure S85. ^{1}H NMR spectrum of involucratusin H (**8**) in $CDCl_3$

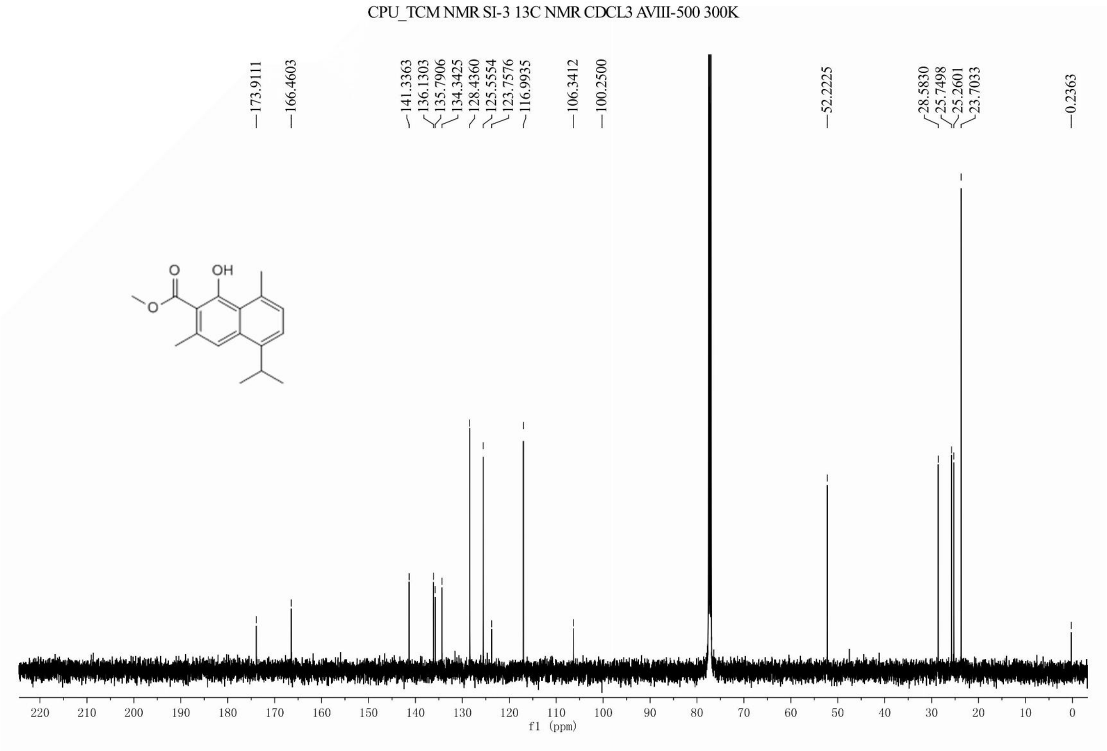

Figure S86. ^{13}C NMR spectrum of involucratusin H (**8**) in $CDCl_3$

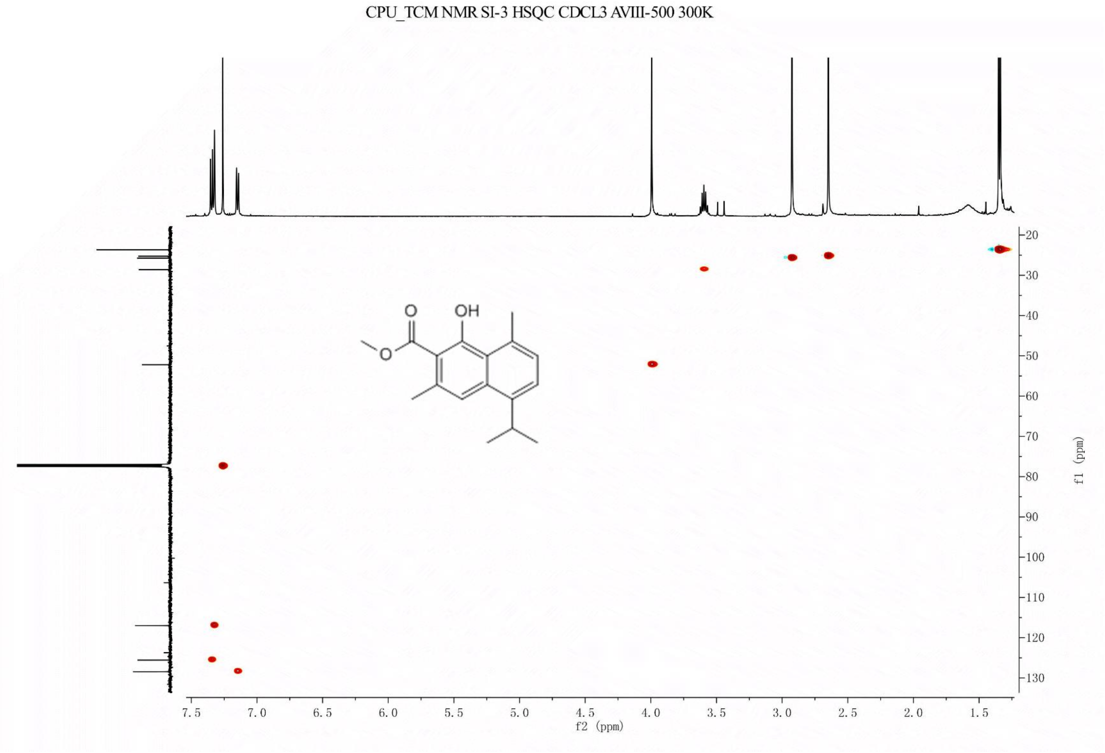

Figure S87. HSQC spectrum of involucratusin H (**8**) in $CDCl_3$

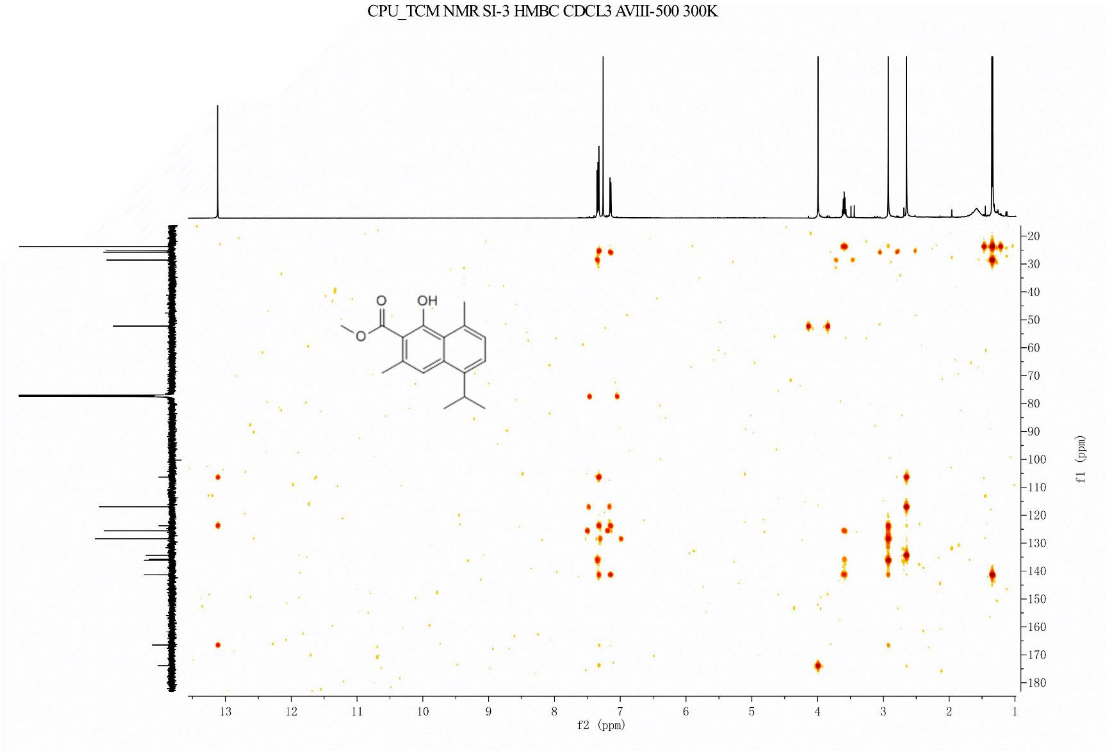

Figure S88. HSQC spectrum of involucratusin H (**8**) in $CDCl_3$

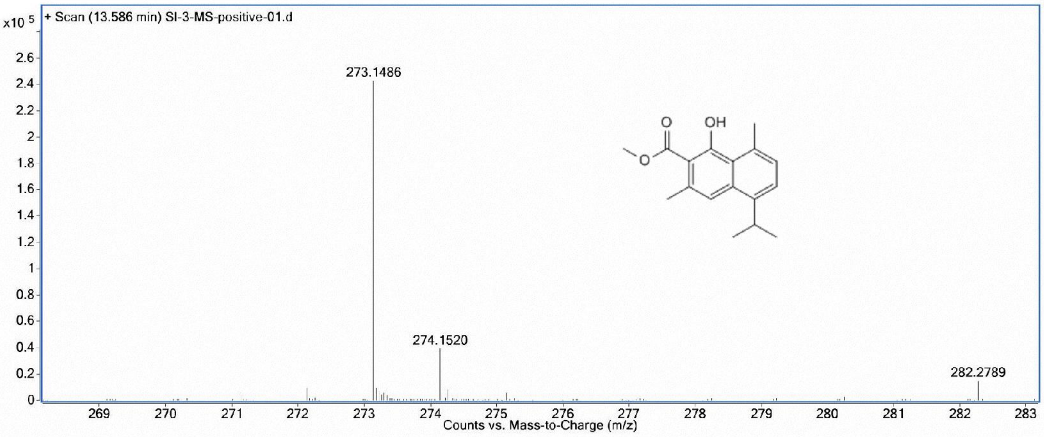

Elemental Composition Calculator

**Target m/z:**	273.1486	**Result type:**	Positive ions	**Species:**	$[M+H]^+$
**Elements:**		C (0-80); H (0-120); O (0-30); N(0-10); Na (0-5)			
**Ion Formula**		**Calcalated m/z**		**PPM Error**	
C17H21O3		273.1485		-0.32	

Agilent Technologies

Figure S89. HRESIMS spectrum of involucratusin H (**8**) in CH_3OH

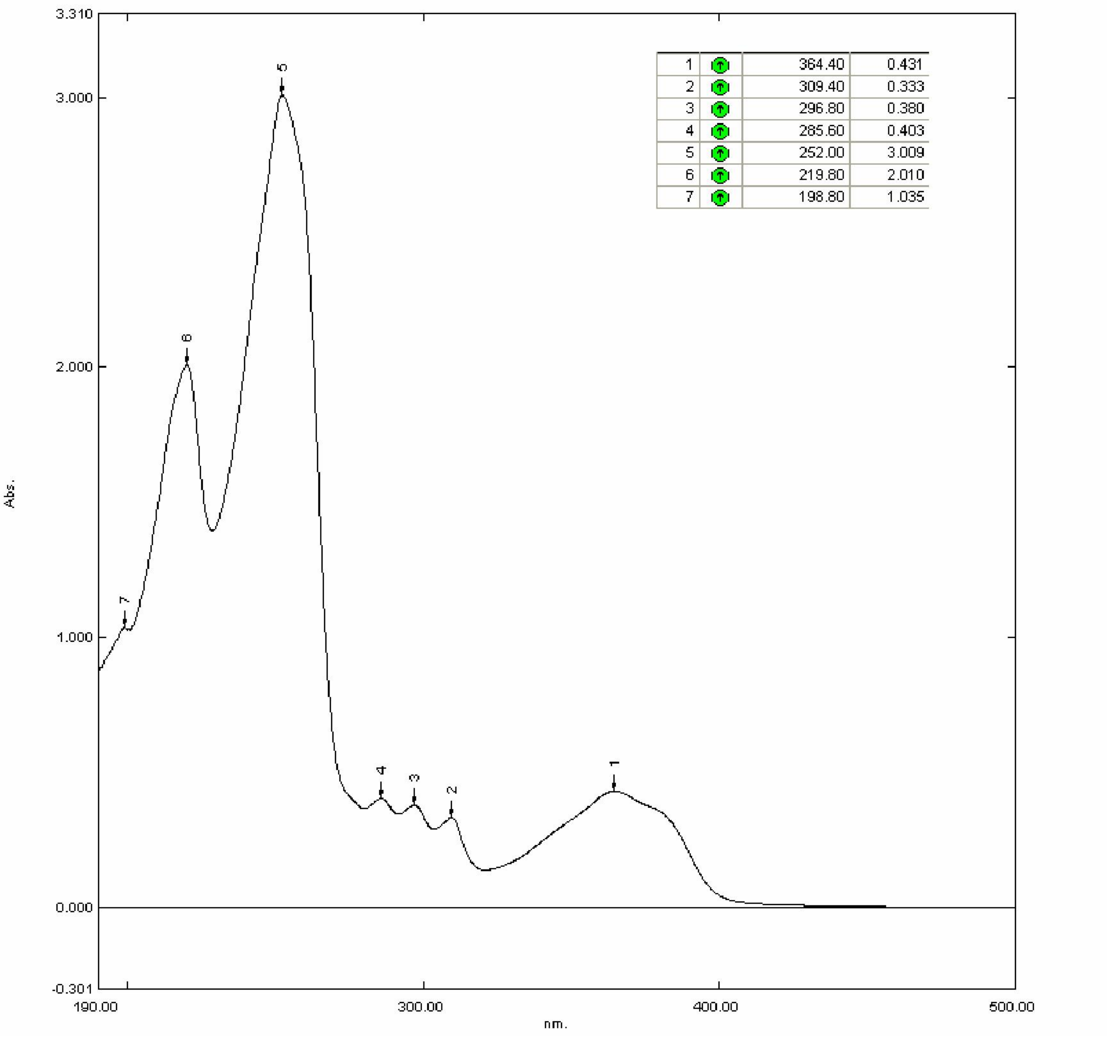

Figure S90. UV spectrum of involucratusin H (**8**) in CH_3OH
